# A cracking-assisted transfer printing technology for high-resolution quantum dot light-emitting diode displays

**DOI:** 10.1038/s41928-026-01670-9

**Published:** 2026-07-17

**Authors:** Jeong-Wan Jo, Yoonwoo Kim, Sanghyo Lee, Jiajie Yang, Yaron Bernstein, Giovanni Francesco Cotella, Feng Zhao, Quan Lyu, Thomas E. Davies, Faris Abualnaja, Greg Chu, Hannah J. Joyce, Stephan Hofmann, Jack A. Alexander-Webber, Bo Hou, Sung-Min Jung, Gehan A. J. Amaratunga, Jong Min Kim

**Affiliations:** 1 Electrical Engineering Division, Department of Engineering, University of Cambridge, Cambridge, UK; 2 Department of Electronics Engineering, Incheon National University, Incheon, Republic of Korea; 3 Department of Materials and Science, Kumoh National Institute of Technology, Gumi, Republic of Korea; 4 Huawei Technologies Research and Development, Ipswich, UK; 5 Huawei Technologies, Shenzhen, China; 6 Huawei Technologies Research and Development, Cambridge, UK; 7 School of Chemistry, Cardiff University, Cardiff, UK; 8 Department of Electrical Engineering, King Fahd University of Petroleum and Minerals, Dhahran, Saudi Arabia; 9 Department of Materials, Loughborough University, Loughborough, UK; 10 School of Physics and Astronomy, Cardiff University, Cardiff, UK; 11 AllFocal Optics, St John’s Innovation Centre, Cambridge, UK; 12 Institute of Critical Materials in Integrated Circuits, Shenzhen Polytechnic University, Shenzhen, China

**Keywords:** Displays, Electrical and electronic engineering, Inorganic LEDs, Quantum dots, Design, synthesis and processing

## Abstract

Inorganic colloidal quantum dot light-emitting diodes could be used to build next-generation electroluminescent displays due to their colour properties and electrical stability. However, to create high-resolution and large-area displays, a pixel integration method is required, which can deposit quantum dot arrays on an active-matrix backplane and maintain uniformity and precision, without colour cross-contamination. Here we report a cracking-assisted transfer printing technology that can be used to pattern high-resolution full-colour pixel arrays over large areas. The technology uses a controlled cracking process to fracture interparticle cohesive bonds between quantum dots. This facilitates subsequent pick-up and transfer to a thin-film transistor backplane with high precision. With the technology, we achieve pixels down to a size of 600 nm with electroluminescent emission and uniform pixelization over areas up to 4 inches. We create a cadmium-free full-colour active-matrix display with a resolution of 341 pixels per inch, as well as a blue active-matrix display with a flexible form factor. Furthermore, the cracking-assisted transfer printing can improve electroluminescence performance—with higher maximum luminance and operational lifetime than other quantum dot patterning techniques—through precise nano-interface control and high quantum dot packing density.

## Abstract (web summary)

Quantum dot pixel arrays can be transfer printed over areas up to 4 inches, and with pixel sizes down to 600 nm, using a cracking step to fracture the cohesive bonds between dots before pick-up and transfer.

## Main

Inorganic colloidal quantum dot light-emitting diodes (QD-LEDs) are promising for next-generation displays due to their broad colour controllability, colour purity, optical efficiency and electrical stability^1–7^. The materials and process technology of electroluminescent (EL) QD-LEDs have already been extensively studied, up to the integration of full-colour display systems^8–14^. QD-LEDs operating in the EL mode are of particular interest in augmented reality and virtual reality applications due to their high-resolution and wide colour gamut^7,15^.

However, to achieve a large-area and high-resolution full-colour display based on QD-LED technology, millions of red, green and blue (RGB) pixels must be integrated on an active-matrix (AM) display backplane^16–19^. Key challenges in the pixelization process include avoiding cross-contamination between RGB pixels during pixelization, controlling stable nano-level interfaces within multilayered EL device architectures and ensuring uniform pixel formation on AM backplanes with non-planar geometries^10,13,20–22^. It will also be preferable if non-toxic quantum dot (QD) materials were used^16–19,23–33^. For example, cadmium (Cd)-based QDs achieve high device performance, but their use raises concerns regarding occupational safety, end-of-life disposal and regulatory compliance. Consequently, it is essential to develop high-performance Cd-free QD materials that exhibit similar device performance during pixel scaling and integration.

Several patterning technologies have been used to integrate QDs onto AM displays, including spin coating (SC)^9,12,16,18,30,31,34^, inkjet printing^8,17,19,26–28^ and transfer printing (TP)^6,10,14,22,35–38^. However, SC can lead to cross-contamination for RGB pixels and colour degradation^13^, and inkjet printing has interfacial instability in nano-layered architectures and restricted droplet size, resulting in a short lifetime and limitations in processing pixels smaller than a few tens of micrometres^7,15,33^. Several full-colour Cd-free AM EL QD displays have been created on low-temperature polysilicon (LTPS) or metal oxide thin-film transistor (TFT) backplanes using photolithography and inkjet printing, but it remains uncertain whether the luminance, external quantum efficiency (EQE) and lifetime are sufficient to support QD-LEDs in large-area and ultrahigh-resolution displays^16–19^. TP exhibits several advantages for QD patterning, including avoiding the use of wet chemicals and forming high-resolution pixels without organic or QD residues. There are two main TP techniques: conventional, using micro-bump stamps^6^ and intaglio, using flat polydimethylsiloxane (PDMS) stamps^35,38^. Conventional TP supports large-area patterning but has difficulties achieving high-resolution dot patterns^6,35^. By contrast, intaglio TP offers high-resolution pixel patterning and design flexibility but is limited to smaller processing areas^35,38^.

Here we report a cracking-assisted transfer printing (CATP) technology for large-area and high-resolution QD patterning on an AM backplane. By exploiting nano-cracks formed using a cracking stamp to break the interparticle QD bonds^39–43^, QD pixels can be transfer printed in various patterns with high uniformity and precise control over large areas. We achieve pixel resolutions ranging from micrometres to 600 nm, as well as pixelization over areas up to 4 inches. We integrate RGB pixels onto AM backplanes with various surface topologies on a 1.5-µm-deep bank structure without cross-contamination between pixels. We fabricated a Cd-free full-colour AM EL QD display with 320 × 360 pixels on a 1.41-inch diagonal LTPS backplane (resolution of 341 pixels per inch (ppi)) with a colour gamut of 124% for standard RGB (sRGB) and 92% for Digital Cinema Initiatives—Protocol 3 (DCI-P3). We also demonstrate a pixelated Cd-free QD-LED with a flexible form factor.

We also show that the CATP method can improve the film photoluminescence quantum yield (PLQY) and minimize local current leakage through nano-interface control and high QD packing density^6,20–22,38^, enabling improved performance and longer lifetimes compared with other QD patterning techniques. EL QD-LED devices processed by CATP compare favourably with other patterned Cd-free QD-LEDs, achieving maximum luminances of 40,150 cd m^−2^ (red), 22,210 cd m^−2^ (green) and 15,360 cd m^−2^ (blue) (Table 1). The red QD-LED device exhibits a *T*_95_ lifetime of 254,897 h at 100 cd m^−2^ (Supplementary Table 1). Our CATP technology offers a viable method for achieving displays with both high-resolution patterning and large-area processability (Supplementary Fig. 1).

**Table 1:** Comparison of research progress of patterned Cd-free QD-LEDs in terms of maximum luminance, peak EQE and minimum pixel size

Year	Patterning method	Maximum luminance (cd m^−2^)	EQE (%)	Lifetime (h)	Minimum pixel size (μm)	Reference
2022	Inkjet printing	91 (B)	0.15 (B)	–	65	^28^
2022	Inkjet printing	100 (R) 1,134 (G) 202 (B)	0.02 (R) 0.14 (G) 0.12 (B)	*T*_50_ at 52 cd m^−2^, 0.37 h (R) *T*_50_ at 151 cd m^−2^, 0.17 h (G) and *T*_50_ at 107 cd m^−2^, 0.04 h (B)	60	^26^
2022	Inkjet printing	2,500 (R) 3,700 (G) 400 (B)	0.7 (G)	*T*_78_ at 1,397 cd m^−2^, 0.27 h (G)	30	^27^
2022	Photolithography	1,700 (R) 5,000 (G) 1,200 (B)	14 (R) 8.3 (G) 9.5 (B)	*T*_90_ at 1,000 cd m^−2^, 156 h (G)	1	^30^
2023	Photolithography	1,600 (R)	0.51 (R)	–	1	^31^
2023	Photolithography	2,800 (R)	1.72 (R)	–	7	^32^
2024	Inkjet printing	103 (G)	–	–	20	^29^
This work	TP	40,150 (R) 22,210 (G) 15,360 (B)	4.54 (R) 1.63 (G) 3.7 (B)	*T*_90_ at 1,000 cd m^−2^, 1.43 h (R) *T*_90_ at 1,000 cd m^−2^, 0.7 h (G) *T*_90_ at 1,000 cd m^−2^, 0.9 h (B)	0.6	

Devices with different colours—red, green, blue and white—are labelled as (R), (G), (B) and (W), respectively.

## CATP for high-resolution QD patterning

**Fig. 1:**
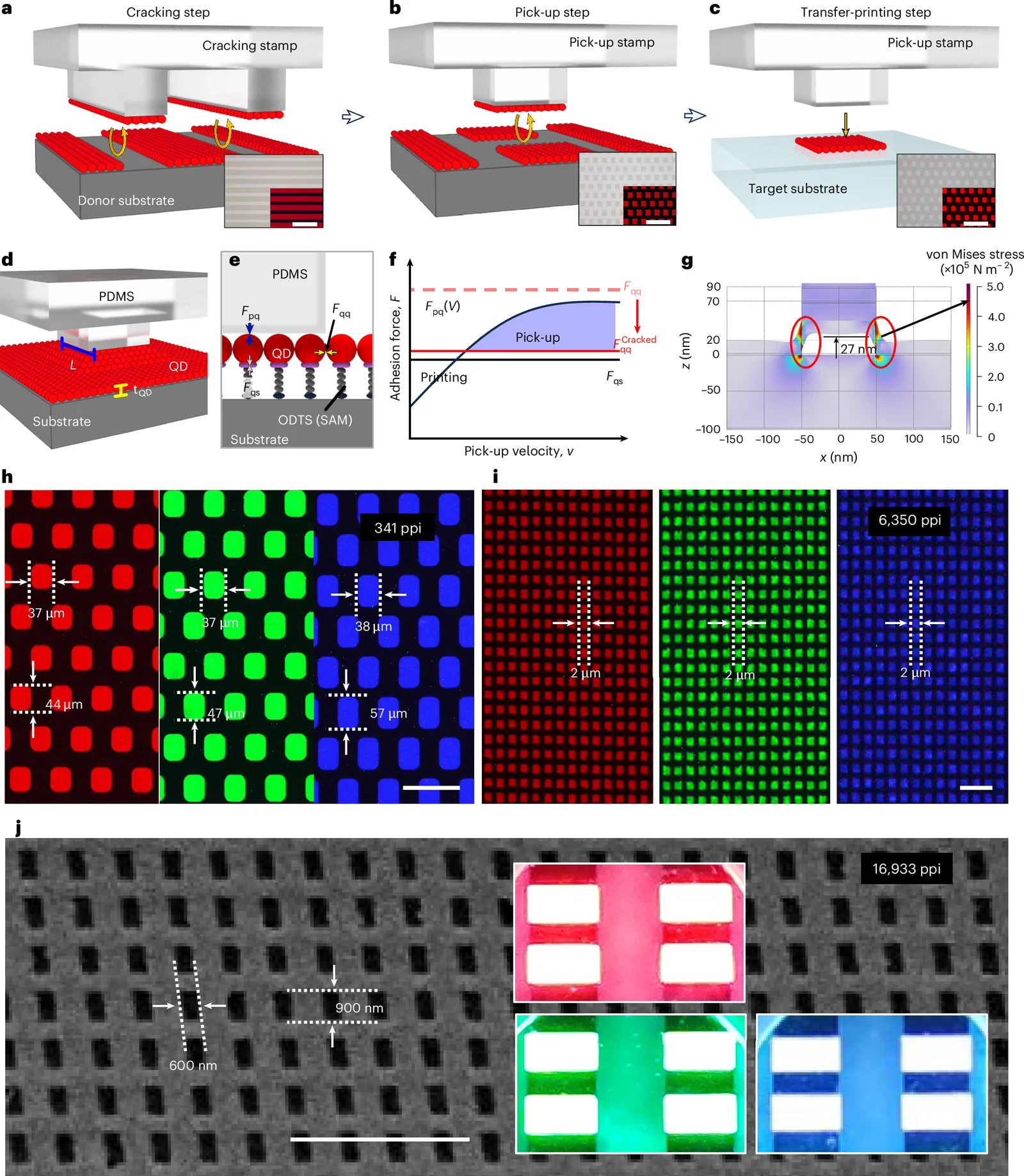
CATP technique for high-resolution QD patterning. **a**–**c**, Key process steps of CATP for QD pixel patterning by a micro-bump PDMS stamp, with optical and PL images of the patterned QD layer on the donor and target substrate (inset). Scale bars, 200 µm. **d**, Schematic of the kinetically controlled TP process involving three layers: micro-bump PDMS stamp, QD layer and substrate. **e**, Molecular-level forces acting on the QD layer during the pick-up process. **f**, Various adhesion forces as a function of pick-up velocity for QD patterning in the CATP process. The intersection of the horizontal line with the monotonically increasing curve represents the critical pick-up velocity for CATP. The red (dot), red (solid) and black horizontal lines correspond to the forces of *F*_qq_ (QD–QD cohesion), *F*_qq_^Cracked^ (cracked QD–QD cohesion) and *F*_qs_ (QD/SAM–substrate interface), respectively. The blue-shaded area indicates the region in which successful pick-up of the QD layer is achievable, as CATP substantially reduces the lateral cohesive force of the QDs. **g**, Simulated von Mises stress in the QD layer for the CATP. **h**,**i**, PL images of 341-ppi dot patterns (**h**) and 6,350-ppi square patterns (**i**) for Cd-free RGB QD pixels by CATP. Scale bars, 100 µm (**h**); 10 µm (**i**). **j**, Scanning electron microscopy image of 16,933-ppi nanoscale QD patterns. Scale bar, 5 µm. Inset: EL images of the QD-LED devices with nanopatterned Cd-free RGB QD pixels.

Figure 1 illustrates the key process steps of CATP for high-resolution QD pixel patterning, with optical and photoluminescence (PL) images of the patterned QD layer on the donor and target substrates. First, the donor substrate is treated with octadecyltrichlorosilane (ODTS) to form a self-assembled monolayer (SAM), and QD films are deposited by the SC of a QD solution onto the substrate (Supplementary Methods and Supplementary Figs. 2 and 3). Then, the CATP process begins by defining the stripe-pattern geometry with a specified resolution during a controlled cracking step, utilizing a cracking stamp to remove QDs from the donor substrate with a high peeling speed of the cracking stamp, where the sufficiently large area of the stripe pattern on the cracking stamp enables effective cracking (Fig. 1a). This cracking step removes the interparticle cohesive forces, thereby facilitating the pick-up of the desired pixel pattern in the subsequent pick-up step. Next, the pick-up stamp with square micro-bumps is used to lift the QD pixels from the prepatterned stripe during the pick-up step with a high peeling speed (Fig. 1b). Finally, the QD pixels are transferred to the target location on the TFT backplane during the TP step with a low peeling speed (Fig. 1c and Supplementary Fig. 4). The fracture mechanics among QDs are precisely controlled through the nano-cracking mechanics of CATP to enable high-resolution QD TP, as explained in more detail below.

In the CATP process, the cracking step first defines partial fracture boundaries by selectively removing the lateral QD–QD cohesive network over a relatively large stripe-patterned region. Because the larger contact area enables efficient fracture initiation at the predefined pixel edges, the remaining QD layer after cracking contains residual edges along the desired pixel boundaries. During the subsequent pick-up step, the micro-bump stamp concentrates tensile stress specifically at the residual edges, enabling localized fracture propagation only along the designed pixel edges. Consequently, unlike conventional TP in which uncontrolled cohesive fracture may occur across the QD film, CATP converts stochastic cracking into a spatially confined fracture process. This two-step fracture-engineering mechanism enables reliable ultrahigh-resolution QD pixelization with high edge fidelity and minimal internal film damage.

The kinetically controlled TP of QDs, which exploits the velocity-dependent adhesion effect of a viscoelastic stamp, is governed by fracture mechanics. This process involves three layers (PDMS, QD and donor substrate) and two interfaces (PDMS/QD and QD/donor substrate), where *L* is the side length of a square PDMS stamp bump (length of the desired QD pixel) and *t*_QD_ is the thickness of the QD layer (Fig. 1d). Nanoscale fracture mechanics in the pick-up step involves three distinct molecular-level forces (Fig. 1e): (1) the pick-up velocity-dependent adhesion force between the PDMS and the QD layer (*F*_pq_); (2) the adhesion force between the QD layer and the ODTS-SAM-treated substrate (*F*_qs_); and (3) the lateral interparticle van der Waals cohesive force at the QD–QD bond (*F*_qq_). Each force at the interface is defined as *F* = *G* × *w*, where *G* is the energy release rate and *w* is the out-of-plane directional width with respect to crack propagation^44^. Supplementary Fig. 5a illustrates the separation of the PDMS/QD and QD/QD interfaces during the pick-up process, driven by the competition among these interfacial forces when the PDMS stamp is subjected to a pulling force perpendicular to the PDMS/QD interface. During peeling, two competing processes occur simultaneously: peeling at the PDMS–QD interface and shear-driven separation at the lateral QD–QD edge. Since *G* at identical velocity conditions is a constant determined by the interfacial properties of the material, *F*_pq_ and *F*_qs_ are proportional to the width of the desired QD pixel (*w* = *L*). In particular, *F*_qq_ is proportional to the out-of-plane width, which is the thickness of the QD layer (*w* = *t*_QD_).

In conventional TP for QD patterning, the primary focus has been on the competing fracture between *F*_pq_ and *F*_qs_, whereas the role of *F*_qq_ has been largely overlooked (Supplementary Fig. 5b,d)^6,35–38^. However, during the pick-up process of small QD pixels (*L* was less than a few hundred micrometres), *F*_qq_ becomes the dominant force in fracture mechanics, surpassing both *F*_pq_ and *F*_qs_ (Supplementary Fig. 5c,e). As the QD pixel size decreases to a few hundred micrometres or less, the pick-up process governed by *F*_qq_ switches from the ‘pick-up on’ regime (*F*_pq_ > *F*_qq_) to the ‘pick-up off’ regime (*F*_qq_ > *F*_pq_) (Supplementary Fig. 6a)^40,41^. Consequently, the conventional TP process exhibits a very low pick-up yield due to the predominance of *F*_qq_ during the high-resolution QD pixelization process (Supplementary Fig. 6b,c)^35^.

In contrast to the conventional TP process, CATP reduces the lateral cohesive force *F*_qq_ of the QD layer on the donor substrate through the cracking step (Fig. 1f). This reduction in *F*_qq_ facilitates nano-cracking along the interparticle bonds at the remaining contact edge of the desired QD pixel during the pick-up process (Fig. 1a,b). In particular, although the effective force distribution varies with the stamp contact area and pixel size, the dominant fracture-driving mechanism in CATP remains the localized overcoming of the lateral QD–QD cohesive force at the pixel boundaries. This mechanism enables the micro-bump stamp in CATP to achieve high-resolution patterning (Fig. 1c), distinguishing it from conventional TP (Supplementary Figs. 6 and 7).

In conventional TP, internal cracking often arises from non-localized stress and uncontrolled fracture initiation within the film, whereas in CATP, stress is deliberately localized at the predefined pixel boundaries during the cracking step, thereby suppressing random crack nucleation in the film interior. Furthermore, the geometry of the cracked QD layer intensifies stress concentration at the pixel edge, leading to amplified local tensile stress. This increased stress enhances the fracture process and facilitates the efficient propagation of nano-cracking along the QD edges. Because crack initiation is spatially confined to the predefined pixel boundaries, uncontrolled crack propagation within the film interior is effectively suppressed, even during the multistep CATP sequence (Supplementary Fig. 8). As a result, the CATP method enables more effective edge fractures of the designed QD pixels, ensuring the easy pick-up of high-resolution QD patterns during the pick-up process.

To support the nano-cracking mechanism, the von Mises stress distribution at the contact edge of the QD layer during the pick-up process is simulated using a three-dimensional solid mechanics model for both conventional TP and CATP (Fig. 1g, Supplementary Fig. 9 and Supplementary Note 1). In the CATP process, a von Mises stress of approximately 4.5 × 10^5^ N m^−2^ is generated at the contact edge of the QD layer, with a larger gap of 27 nm compared with conventional TP. Additionally, CATP exhibits approximately twice the tensile stress compared with the conventional TP method, as the cracking process eliminates *F*_qq_, exerting stronger stress to fracture the edge of the QD layer. Overall, the simulations confirm that the modulation of *F*_qq_ and nano-cracking mechanics via controlled cracking is a key factor in the success of CATP.

Figure 1h demonstrates the PL images of uniformly patterned Cd-free RGB QD pixel arrays. The red (37 µm × 44 µm, indium phosphide (InP)/zinc sulphide (ZnS) core/shell QDs), green (37 µm × 47 µm, InP/ZnS core/shell QDs) and blue (38 µm × 57 µm, zinc telluride selenide (ZnTeSe)/zinc selenide (ZnSe)/ZnS core/shell QDs) pixels for full-colour displays are successfully patterned by CATP. CATP can pattern QDs with diverse nanomaterials and various surface ligands (Supplementary Fig. 10) across a wide range of film thicknesses (Supplementary Figs. 11 and 12) for various device applications. It is noted that since the force between the QD interlayers is higher than *F*_pq_, no interlayer delamination occurs (Supplementary Note 2 and Supplementary Figs. 4 and 13). In addition, the slightly rounded pixel outlines observed in Fig. 1h originate from intentional geometric design rather than process variability. Introducing a controlled curvature at pixel boundaries alleviates local stress concentration during the cracking and pick-up steps, enabling smoother fracture propagation and stabilizing the definition of the final QD patterns.

Additionally, for high-resolution pixel patterning, 2 μm × 2 μm-sized Cd-free red, green and blue QD pixel arrays (6,350 ppi) are successfully fabricated by CATP (Fig. 1i). For further downsizing pixels to nanometre-scale dimensions, 600 nm × 900 nm rectangular dot QD pixel arrays (16,933 ppi) are successfully patterned by CATP with a ultrahigh-precision master mould (Fig. 1j). The inset shows the first EL image of a 600 nm × 900 nm dot-patterned Cd-free red, green and blue QD-LED device with a luminance of 9,000 cd m^−2^, 5,330 cd m^−2^ and 2,755 cd m^−2^ at 8 V, respectively. The uniformity and reproducibility of the CATP process across the various pattern sizes are quantitatively confirmed by the statistical analysis of relative pixel-area distributions from tens-of-micrometres and micrometre to sub-micrometre QD patterns (Supplementary Figs. 14–16), as detailed in the Supplementary Note 3. In addition to rectangular features, the CATP process can define any pixel geometry, enabling the fabrication of free-form QD pixels tailored to diverse device architectures and application requirements (Supplementary Fig. 17 and Supplementary Note 4). Consequently, high-resolution QD pixels are successfully achieved, demonstrating the process capability down to nanometre-scale EL RGB pixels (Supplementary Note 5 and Methods), which have not been achieved by other technologies such as micro-light-emitting diodes^45,46^ (up to 8,500 ppi) and organic light-emitting diodes (OLEDs)^47,48^ (up to 10,000 ppi).

## Pixelization by CATP in large-area, high-resolution and non-planar surface

**Fig. 2:**
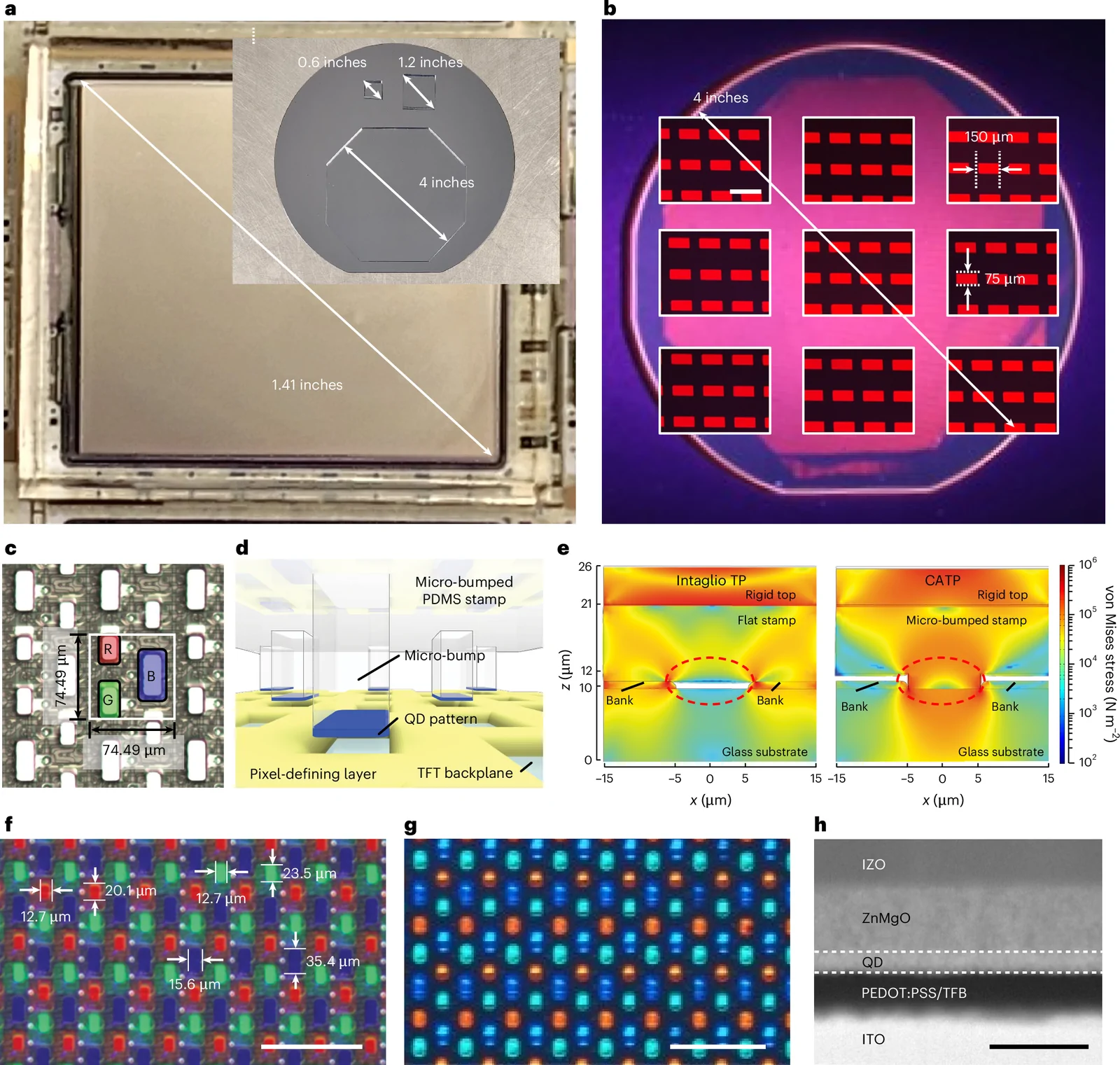
Various QD pixel patterns in a large-area, high-resolution and non-planar AM TFT backplane by CATP. **a**, Full contact of a micro-bump PDMS stamp on the non-planar surface of a 1.41-inch LTPS TFT backplane without air-bubble entrapment. Inset: various-sized micro-bump PDMS stamps (0.6 inches, 1.2 inches, and 4 inches diagonally) on a planar wafer substrate without air-bubble entrapment. **b**, PL images of pixelated 150 μm × 75 μm red QD patterns on a 4-inch glass substrate obtained by CATP. **c**, LTPS TFT backplane with a 1-pixel size of 74.49 µm × 74.49 µm. A seven transistor–one capacitor circuit is used for AM pixel driving. **d**, A micro-bump PDMS stamp in contact with the TFT backplane in CATP for the QD pixel formation into the 1.5-μm-deep bank structure. **e**, Simulated von Mises stress in the PDMS stamp for the intaglio TP (left) and CATP (right) on the bank-structured TFT backplane. **f**,**g**, PL (**f**) and EL (**g**) images of the Cd-free RGB QD pixels fabricated on the TFT backplane by CATP. Scale bar, 150 µm. **h**, Cross-sectional HAADF-STEM image of multilayered stack architecture of the InP red QD-LED device by CATP. Scale bar, 100 nm.

Building on the foundational principles and demonstrated capabilities of CATP for achieving high-resolution QD pixel patterning, we evaluate its practical application as a process technology for fabricating full-colour AM EL QD display systems, which encompasses the use of Cd-free materials, large-area processing, ultrafine pixel patterning and nano-interfaced device architecture. Figure 2 demonstrates various QD pixel patterns on a large-area, high-resolution, non-planar AM TFT backplane (Supplementary Fig. 18 and Supplementary Notes 6 and 7) for the implementation of full-colour AM EL QD display systems via CATP.

As a reference study, the intaglio TP method, one of the most actively researched techniques in the field of QD TP, was preliminarily tested to assess its feasibility for fabricating full-colour AM EL QD display systems concerning ultrahigh-resolution and large-area QD pixel patterning under the same conditions (Supplementary Note 8 and Supplementary Fig. 19). Although a pixel size down to 100 nm in a stripe-patterned QD layer (84,666 ppi) was successfully processed, demonstrating a nanopatterned EL QD-LED device for ultrahigh-resolution applications using the TP method, it is important to note that this method is limited in its capability for large-area printing. Although we aimed to extend intaglio TP to cover large areas, targeting up to a 4-inch diagonal on a planar substrate and an AM backplane with non-planar surface topology, air-bubble entrapment was observed in both planar and non-planar surface topologies (Supplementary Fig. 20a–c), greatly limiting the large-area processability. This limitation originates from interfacial air pockets confined within nanoscale gaps and the absence of continuous air-release pathways at the stamp–substrate interface, which limits large-area processability (Supplementary Fig. 20d–f).

In contrast to intaglio TP, the micro-bump PDMS stamp used in CATP enables large-area processability by introducing pathways that expel air, ensuring seamless and complete adhesion to both donor and target substrates (Supplementary Fig. 20g–i). This advancement arises from the formation of interfacial gaps between adjacent micro-bumps, which provide continuous air-escape pathways during contact formation, as schematically illustrated in Supplementary Fig. 20j–l. With a sufficiently large micro-bump height, these pathways remain open across both planar and non-planar surfaces (Supplementary Fig. 21). With these micro-bump stamps, CATP does not suffer from air-bubble entrapment in either planar or non-planar surface topologies (Fig. 2a), enabling a high-resolution uniform pixelation process on a large-area 4-inch substrate (Fig. 2b and Supplementary Fig. 22). The ability to fabricate high-resolution QD arrays in a single transfer print using a 4-inch stamp, combined with tape cleaning of the PDMS surface (Supplementary Fig. 23), demonstrates the cost-effectiveness over a large number of repeated cycles (Supplementary Fig. 24), less material wastage (Supplementary Fig. 25) and high-throughput capabilities of CATP for the implementation of AM EL QD display systems. In addition, an eight-step stitching process enabled the transfer of an 8-inch-scale QD pattern, demonstrating that CATP is not intrinsically area limited and can be extended to larger substrates (Supplementary Fig. 26).

Furthermore, CATP shows good process control with uniform and air-bubble-free pixelization at the various non-planar surface topologies. The role of the bump-structured stamp is particularly important because the AM TFT backplane exhibits various non-planar surface topologies produced by pixel-defining bank structures to avoid strong edge field effects that can induce device failure and to prevent crosstalk, thereby securing a stable pixel structure. Figure 2c shows a top view of the TFT backplane with the red (12.7 μm × 20.1 μm), green (12.7 μm × 23.5 μm) and blue (15.6 μm × 35.4 μm) emissive areas in a 74.49 μm × 74.49 μm pentile pixel layout. The micro-bump PDMS-stamp-based CATP process, which ensures the stable deposition of RGB QD pixels into 1.5-μm-deep bank structures, is illustrated in Fig. 2d. The high conformality of the micro-bump PDMS stamp enables reliable pixel formation on non-planar surface topologies, demonstrating the versatility of the CATP process.

Figure 2e shows the simulated cross-sectional von Mises stress distribution during the transfer printing steps for both intaglio TP using flat stamps and CATP using micro-bump stamps on a bank-structured TFT backplane. Although the flat stamp used in the intaglio TP process cannot effectively concentrate the force within the bank structures, the micro-bump PDMS stamp in CATP is inserted into the bank structure and applies stress directly to the intended areas. On the basis of this principle, the micro-bump stamp concentrates the applied pressure on the target pixel regions, enabling the precise TP of QDs into the pixel-defining bank structures.

Full-colour RGB pixel array patterned by CATP is successfully integrated onto the TFT backplane with a 1.5-μm bank structure, accompanied by clear PL images as shown in Fig. 2f and Supplementary Fig. 27. The array also demonstrates uniform and clear EL emission when driven by the TFT backplane (Fig. 2g). To ensure that these RGB QD patterns are consistently formed on the pentile LTPS backplane, we established a set of alignment-tolerant design rules for both QD pattern geometry and the corresponding PDMS bump structures. The full design methodology is provided in Supplementary Note 9. These rules define the allowable placement margin relative to the emissive areas, constrain the QD pattern sizes to remain within the midpoint boundaries between neighbouring subpixels, and specify the stripe- and bump-based layouts used in the cracking and pick-up steps (Supplementary Figs. 28 and 29). The overall alignment accuracy of CATP by our equipment (Supplementary Fig. 30) was measured to be less than 4 µm in both horizontal and vertical directions (Supplementary Fig. 31), and the transfer yield is nearly 100% over the entire area (Supplementary Fig. 32). It is anticipated that enhanced stamp robustness and equipment precision will enable reliable large-area integration of sub-micrometre- and nanometre-scale CATP-patterned pixels.

Figure 2h shows the cross-section of the EL QD-LED device taken by high-angle annular dark-field scanning transmission electron microscopy (HAADF-STEM). The device architecture of the EL QD-LED pixel is a stack of indium tin oxide (ITO)/silver (Ag)/ITO triple layer for a reflective anode electrode, poly(3,4-ethylene dioxythiophene):polystyrene sulfonate (PEDOT:PSS) for a hole injection layer, poly[(9,9-dioctylfluorenyl-2,7-diyl)-*co*-(4,40-(*N*-(4-*s*-butyl phenyl)) diphenylamine)] (TFB) for hole transport layer (HTL), Cd-free and Cd-based QDs for an emissive layer, zinc magnesium oxide (ZnMgO) for an electron transport layer (ETL) and indium zinc oxide (IZO) for a transparent cathode electrode (Supplementary Fig. 33). As shown in the cross-sectional HAADF-STEM image of the multilayer stacked structure of EL QD-LED pixels, the concentrated pressure during CATP enables the successful deposition of QD layers on non-planar surfaces with bank structures. In addition, atomic force microscopy of SC QD and CATP QD films on a TFB-coated Si substrate reveals that the CATP process facilitates the formation of well-defined nano-interfaces of the QD layers, exhibiting higher uniformity compared with SC-based films (Supplementary Fig. 34).

## Electro-optical characteristics of QD-LEDs produced via CATP

**Fig. 3:**
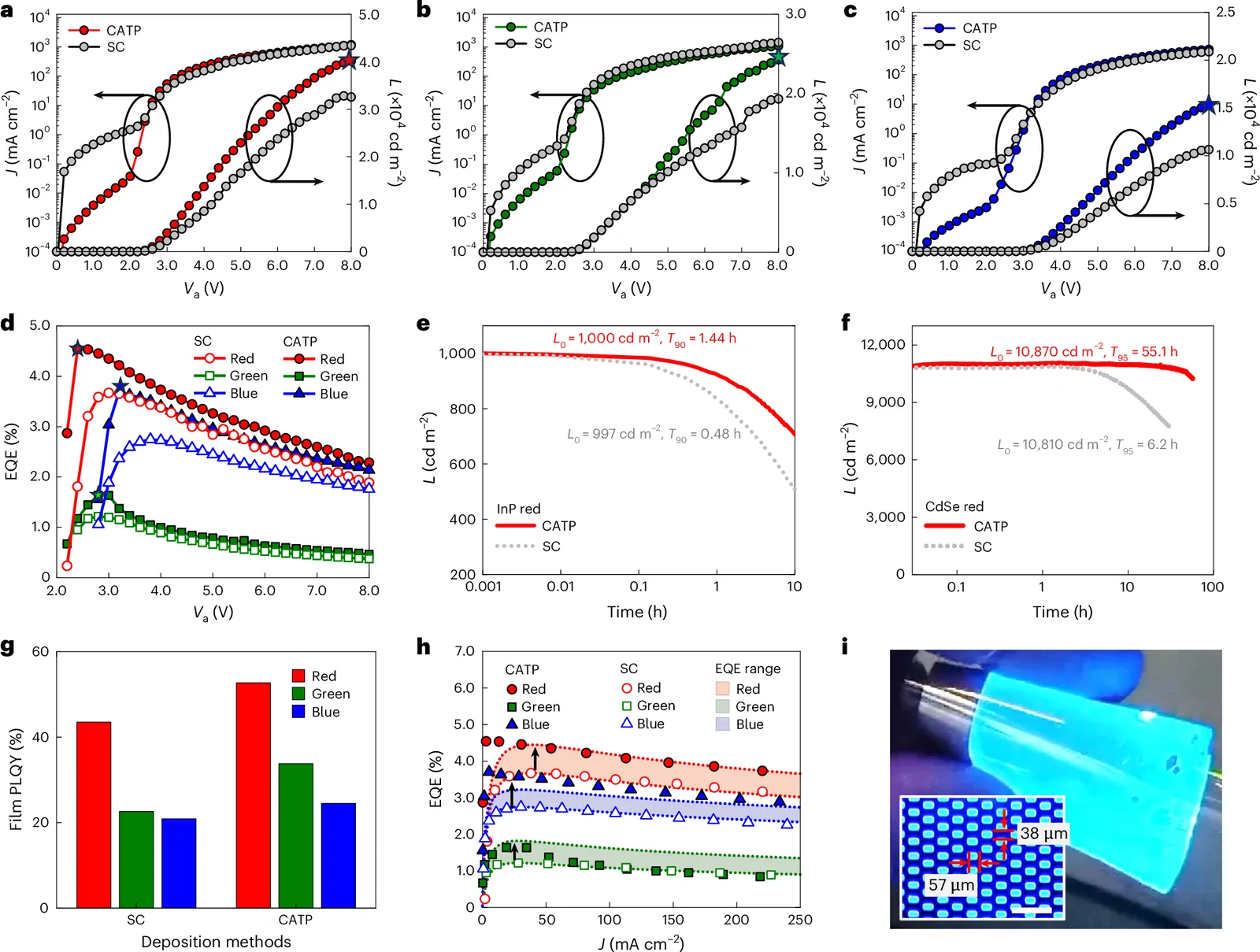
EL characteristics of QD-LEDs fabricated by CATP process. **a**–**c**, Current density *J* and luminance *L* as a function of the applied voltage *V*_a_ for InP-based red (**a**), InP-based green (**b**) and ZnTeSe-based blue (**c**) QD-LED devices fabricated by CATP and SC. **d**, EQE curves as a function of current density for the respective QD-LED devices fabricated by CATP and SC. **e**, Lifetime measurement of the Cd-free red QD-LEDs fabricated by CATP and SC under initial luminance *L*_0_ of 1,000 cd m^−2^. **f**, Lifetime measurement of the Cd-based red QD-LEDs fabricated by CATP and SC under *L*_0_ of 10,000 cd m^−2^. **g**, Experimental PLQYs of the Cd-free red, green and blue QD films fabricated by CATP and SC. **h**, EQE as a function of current density simulated by the ABC model, along with the experimental PLQYs of CATP- and SC-based Cd-free QD films. **i**, Demonstration of flexible-form-factor Cd-free pixelated EL QD-LED by CATP with 57 µm × 38 µm blue emissive pixels on a 4 cm × 4 cm active area. Scale bar, 200 µm. Source data

The impact of CATP on the electro-optical properties of Cd-free and Cd-based EL QD-LED devices is analysed with respect to the conventional SC method. Figure 3 presents the electro-optical characteristics of Cd-free and Cd-based EL QD-LED devices processed by CATP and SC. The EL QD-LED devices are a stack of ITO (anode)/PEDOT:PSS (hole injection layer)/TFB (HTL)/ZnMgO (ETL)/Al (cathode) with Cd-free and Cd-based QD materials. Figure 3a–c shows the current density and luminance curves as a function of the applied voltage for InP-based red and green, and ZnTeSe-based blue EL QD-LED devices fabricated using CATP and SC processes. The Cd-free red, green and blue EL QD-LED devices fabricated by CATP exhibit luminance values of 40,150 cd m^−2^, 22,210 cd m^−2^ and 15,360 cd m^−2^, respectively, at 8 V. In comparison, the Cd-free red, green and blue EL QD-LED devices fabricated by SC exhibit luminance values of 32,583 cd m^−2^, 17,850 cd m^−2^ and 10,650 cd m^−2^, respectively, at the same voltage. The devices fabricated by CATP exhibit higher luminance compared with those fabricated by the SC method. An increase in the maximum luminance achieved by CATP is also observed in Cd-based QD-LEDs (Supplementary Fig. 35a–c). It is notable that our Cd-free EL QD-LED devices fabricated by CATP exhibit a maximum luminance that surpasses that of Cd-based QD-LED devices fabricated by conventional TP^6,10,22,35–37^. Moreover, these devices exhibit the highest luminance among reported patterned Cd-free EL QD-LEDs (Table 1)^26–32^.

The EQE curves corresponding to the Cd-free and Cd-based red, green and blue EL QD-LED devices for the applied voltage are plotted in Fig. 3d. The maximum EQEs of Cd-free red, green and blue EL QD-LED devices fabricated by CATP are 4.54%, 1.63% and 3.70%, respectively, whereas those of EL QD-LED devices fabricated by SC are 3.67%, 1.21% and 2.74%, respectively. Cd-based red, green and blue EL QD-LED devices fabricated by CATP also exhibit a higher maximum EQE than those fabricated by SC (Supplementary Fig. 35d–f), with the Cd-based red QD-LED reaching 23.8%, the highest EQE reported among transfer-printed Cd-based EL QD-LEDs (Table 1). Thus, both Cd-free and Cd-based red, green and blue EL QD-LED devices fabricated by CATP exhibit a higher maximum EQE compared with those fabricated by SC. Supplementary Fig. 36 shows the histograms of the maximum EQE for 20 devices in Cd-free red, green and blue EL QD devices fabricated by CATP, demonstrating their reproducibility.

The device lifetimes of the Cd-free and Cd-based red EL QD-LEDs fabricated by CATP and SC are compared in Fig. 3e,f. Overall, the CATP QD-LEDs exhibit superior operational lifetime characteristics compared with those fabricated by SC. In particular, the Cd-based red QD-LED device demonstrated a *T*_95_ lifetime of 55.1 h at 10,870 cd m^−2^, corresponding to 254,897 h at 100 cd m^−2^, which is particularly longer than the operational lifetimes of Cd-based QD-LEDs processed using other QD patterning technologies (Supplementary Table 1).

These improvements are attributed to the dense packing of the QD layer by CATP, which is facilitated by the mechanical force applied during the pick-up and TP steps. To verify the improved density of the QD layer, we measured the refractive index of CdSe/ZnS red and ZnSeTe/ZnSe/ZnS blue QD films prepared by SC and TP using spectroscopic ellipsometry (Supplementary Fig. 37). Transfer-printed films exhibit a higher refractive index than spin-coated films, indicating greater film densification^49^. Consistently, CdSe/ZnS red QD-LEDs fabricated via TP show lower series resistance (*R*_s_) and charge-transfer resistance (*R*_ct_), further supporting the formation of a densely packed QD film (Supplementary Fig. 38a). In addition, capacitance–voltage (*C*–*V*) measurements reveal that transfer-printed QD-LEDs exhibit a lower capacitance across the entire voltage range compared with spin-coated devices, indicating suppressed charge accumulation in the charge transport layers (Supplementary Fig. 38b). The dense packing of QD nanoparticles results in more stable charge injection into the QD layer and mitigated device degradation caused by localized current concentration^6,10,11,14,21,22,38^_._

To investigate the influence of the QD deposition method on the reduced leakage current, we fabricated single-carrier devices. Both electron-only and hole-only devices exhibited lower current density at voltages below the turn-on voltage compared with the spin-coated films, effectively suppressing carrier injection in this region (Supplementary Fig. 39a,b). In addition, the analysis of EL spectra at 600 mA cm^−^^2^ shows that SC devices exhibit higher emission intensity in the blue region, indicating noticeable current leakage through additional pathways that allow some injected electrons to bypass the QD layer and reach the HTL directly (Supplementary Fig. 39c). This highlights the main advantages of CATP as a non-destructive and well-controlled nano-interface for Cd-free QD patterning.

We also conducted measurements of film PLQY and performed a theoretical analysis using the ABC model to further investigate the enhancement of electro-optical properties. Figure 3g shows the experimental PLQY of the QD films processed by CATP and SC. The red, green and blue QD films produced by CATP exhibit PLQYs of 52.7%, 33.8% and 24.5%, respectively, demonstrating higher PLQY compared with the QD films produced by SC, which show PLQYs of 43.5%, 22.6% and 20.9% for the same colours. The higher PLQY of CATP-processed QD films originates from their improved morphological uniformity and the reduced density of surface-related trap states, as evidenced by the lower surface roughness observed in atomic force microscopy measurements (Supplementary Fig. 34). This enhanced film quality suppresses defect-mediated non-radiative recombination, in agreement with prior reports demonstrating that nanoscale roughness strongly influences PLQY and overall radiative efficiency in QD-LEDs^50,51^. Furthermore, the PLQY of SC-based Cd-free QD films generally decreases after multiple continuous wet processing steps of RGB pixels^27,29–32^, whereas our CATP process shows robust PLQY due to its discrete pixelization method. The EQEs rely on the PLQY and carrier recombination parameters of the QD film, according to the theoretical ABC model (Supplementary Note 10)^52^. The theoretical EQE–current density curves are calculated to illustrate the impact of PLQY by CATP and SC, along with the experimental EQE curves by CATP and SC (Fig. 3h). Experimental EQE–current density curves show good agreement with the theoretical ABC model. The EQE curves by CATP are higher than those by SC, which is attributed to the superior PLQY values of CATP-processed QD films. In addition, a ZnTeSe blue QD-LED device with pixel dimensions of 57 μm × 38 μm and an active area of 4 cm × 4 cm was successfully fabricated on a flexible substrate using CATP, highlighting its potential for flexible and wearable applications (Fig. 3i).

## CATP-enabled integration of full-colour Cd-free AM EL QD display system

**Fig. 4:**
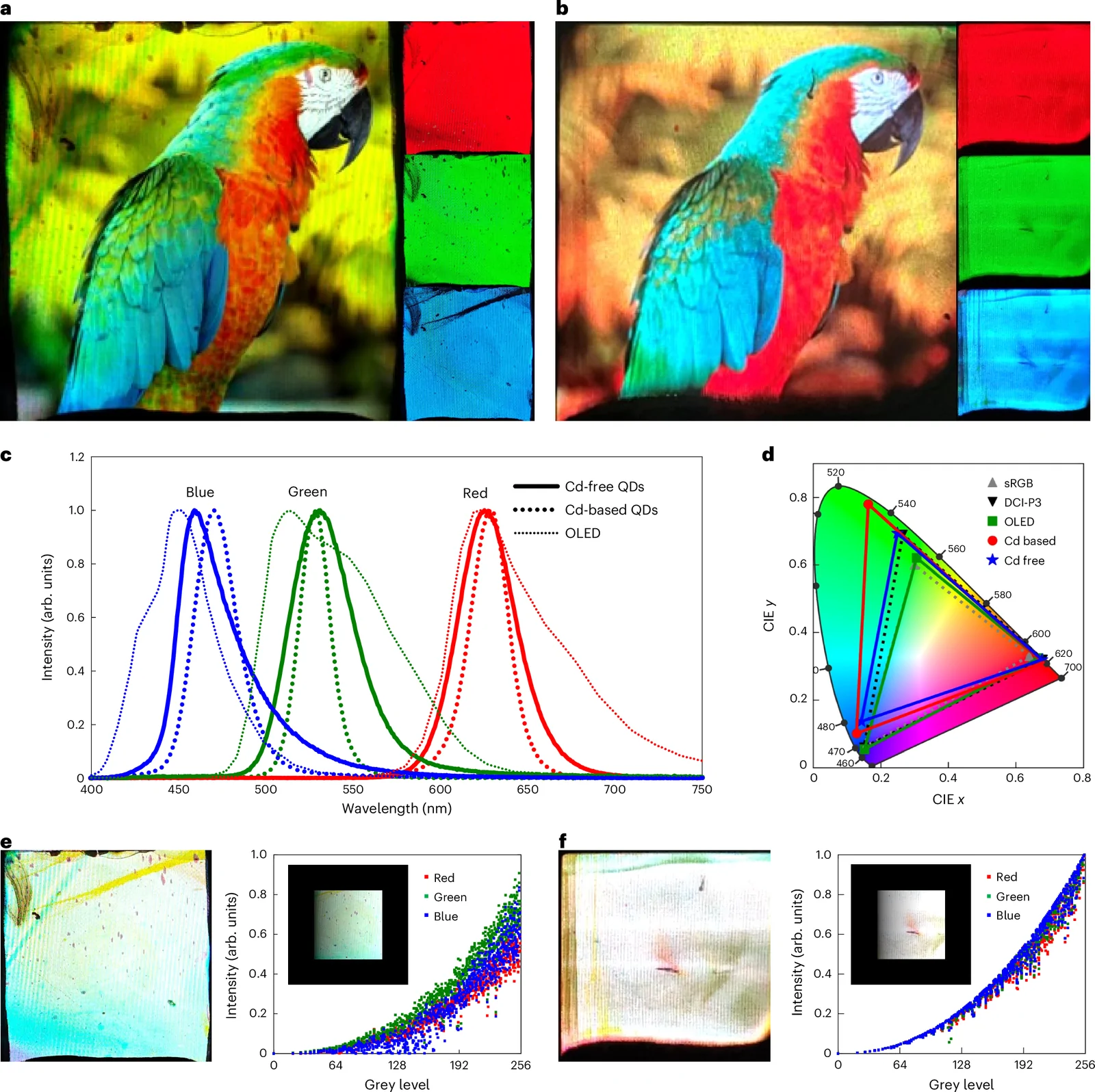
Full-colour Cd-free AM EL QD display system integration. **a**,**b**, The 1.41-inch full-colour AM EL QD display system made by CATP on an AM TFT backplane with 320 × RGB × 360 pixels (341 ppi) based on Cd-free (**a**) and Cd-based (**b**) QD materials, along with RGB EL colour images. **c**, Normalized spectra of red, green and blue EL images for Cd-free and Cd-based AM EL QD displays, along with the EL spectra of red, green and blue for the conventional OLEDs. **d**, Chromaticity in CIE 1931 colour space for the Cd-free and Cd-based AM EL QD displays. The chromaticity of the sRGB and DCI-P3 standards, as well as the conventional OLED displays, are marked together for comparison. **e**,**f**, White images of AM Cd-free (**e**) and Cd-based (**f**) EL QD displays along with the intensity distribution on gamma curves for the red, green and blue colours. Inset: gamma test patterns on each display. Credit: original image in **a** and **b**, Joakim Lloyd Raboff. Source data

Finally, a full-colour Cd-free AM EL QD display system on the TFT backplane is demonstrated in Fig. 4a with InP/ZnS for red and green and ZnTeSe/ZnSe/ZnS for blue QD materials. The system is compared with a Cd-based (CdSe/ZnS for red, green and blue) full-colour AM EL QD display system, also made by CATP (Fig. 4b). The full-colour parrot EL images with red, green and blue EL images are presented on both Cd-free and Cd-based AM EL QD display systems. In both cases, the display panel features an active area of 1.41-inch in diagonal size with 320 × RGB × 360 pixels (341 ppi in display resolution). Both AM EL QD display systems deliver high-quality images with bright and vivid colours over a large area. However, CATP exhibits superior uniformity compared with intaglio TP, which shows pronounced colour mura and defective pixels caused by air-bubble entrapment (Supplementary Figs. 19 and 32). In addition, individual red, green and blue EL images of the display fabricated by CATP demonstrate each colour without any visible colour crosstalk (Fig. 4a,b). This is attributed to the bank structures of the LTPS and emission layer patterning, which suppress lateral leakage between adjacent pixels to prevent colour mixing (Supplementary Fig. 40). The observed defects in the displays originate from the laboratory-scale setup and the SC processes used for the HTL and ETL (Supplementary Fig. 41), rather than from the CATP process itself, which exhibits a near-100% transfer yield (Supplementary Fig. 32).

The system-level colour properties of Cd-free and Cd-based AM EL QD displays are analysed and compared with conventional OLEDs as the reference^53^ (Fig. 4c,d). In Fig. 4c, Cd-free QDs exhibit wider full-width at half-maxima of 39 nm (InP red), 43 nm (InP green) and 38 nm (ZnTeSe blue) in contrast to the narrower full-width at half-maxima of 26 nm (red), 21 nm (green) and 29 nm (blue) in Cd-based QDs. This indicates that the spectra of Cd-free QD displays are wider compared with those of Cd-based QD displays. At the material level, this results from the larger deviation of the core diameter and non-uniform shape in the Cd-free QD materials (Supplementary Fig. 42). Moreover, the wider full-width at half-maximum of Cd-free QDs is related to their higher sensitivity to oxidation^54^, particularly during the precise alignment process conducted in ambient air, compared with Cd-based QDs. However, Cd-free QD materials still exhibit a narrower spectrum compared with OLEDs, due to the band edge emission resulting from the quantum confinement effect.

Figure 4d shows the chromaticity coordinates of red, green and blue images within the Commission Internationale de l’éclairage (CIE) 1931 colour space for both Cd-free and Cd-based AM EL QD displays. The chromaticity coordinates for the sRGB and DCI-P3 standard colour spaces, as well as for conventional OLED displays, are marked in the CIE colour space for comparison. The Cd-free AM QD-LED display shows a colour gamut of 124% for sRGB and 92% for DCI-P3, whereas conventional OLED displays show a colour gamut of 112% for sRGB and 82% for DCI-P3. The Cd-free AM EL QD display offers superior colour performance compared with conventional OLED displays. Moreover, the Cd-based AM QD-LED display exhibits the highest colour gamut of 164% for sRGB and 121% for DCI-P3, which is attributed to the high electro-optical quality of Cd-based QDs.

The intensity distributions of white colour emission for full-colour Cd-free and Cd-based AM EL QD displays are analysed on gamma test patterns to gain a better understanding of the importance of balanced white colour emission, which is a crucial element in a white lighting system (Fig. 4e,f). Interestingly, the Cd-free AM EL QD display (Fig. 4e) exhibits wider intensity distributions across various grey levels for red, green and blue colours compared with the Cd-based AM EL QD display (Fig. 4f). Under the same pixel integration process conditions and display driving environments, this wider distribution in the Cd-free AM EL QD display indicates a decrease in colour consistency emitted from each pixel, which arises from the material differences between Cd-free and Cd-based QDs. This result is consistent with the previous analysis of system-level colour properties alongside the material-level analysis.

On the basis of the above results, the CATP technology, combined with high-quality Cd-free QD materials^23–25^, is expected to provide superior electro-optical performance for Cd-free EL QD displays. Consequently, the CATP method for creating Cd-free EL QD devices is a strong candidate for future eco-friendly, large-area and high-resolution displays for next-generation immersive augmented reality/virtual reality systems. Building on the current results, future work will focus on improving alignment strategies, stamp robustness and equipment precision to enable the reliable large-area integration of sub-micrometre- and nanometre-scale CATP-patterned pixels.

## Conclusions

We have reported a cracking-assisted TP technology for creating Cd-free QD full-colour EL displays on an AM backplane. CATP offers large-area processability, ultrahigh-resolution and versatility in substrate geometry, including flexible, stretchable and textile-based backplanes. We demonstrated uniform pixelization over 4-inch substrates and pixel sizes down to 600 nm. Full-colour RGB pixels on an AM backplane with non-planar surface topology were also integrated without cross-contamination between pixels. Using CATP, we created a Cd-free full-colour AM EL QD display with 320 × RGB × 360 pixels on an active area of 1.41-inch LTPS TFT backplane (341 ppi) with a colour gamut of 124% for sRGB and 92% for DCI-P3. A pixelated Cd-free EL QD display with a flexible form factor was also fabricated. Compared with other QD patterning technologies, CATP exhibits improved electro-optical device performance and longer operational lifetime due to precision nano-interface control, which improves film PLQY and minimizes local current leakage via the high packing density of QDs. CATP should provide opportunities for further advancements in full-colour EL QD display technologies towards future high-resolution augmented reality/virtual reality and large-area display applications.

## Methods

### CATP for high-resolution QD patterning

Two types of micro-bump structure PDMS stamp were used for the CATP process. The process begins with the cracking of the QD layer, formed in a stripe shape to eliminate the interparticle forces among the QDs at the edge of the designed QD pixel shape. The cracking stamp has micro-bumps with a stripe pattern. The pick-up stamp for the picking up and transferring of the QD layer has a specific pixel design including a square or dot pattern.

CATP fabricates high-resolution QD patterns through the following five steps: (1) preparation of a donor substrate with ODTS, (2) deposition of a QD film by the SC of a QD solution onto the donor substrate, (3) stripe-pattern geometry formation by fast pick-up using a cracking stamp to remove the lateral interparticle van der Waals cohesive force, (4) fast pick-up of cracked QDs from the donor substrate using a pick-up stamp to achieve the desired QD pixel pattern and (5) TP of the picked up QD layer onto the target substrate. In our experiment, the optimal stamp velocity in the pick-up step is set to be 500 mm s^−1^.

### Device architectures for AM EL QD display

As a device architecture for the emissive pixel, top-emission QD-LED architecture with the reflective ITO/Ag/ITO anode electrode is used in the AM EL QD display (Supplementary Fig. 33a). PEDOT:PSS (AI 4083, Ossila) is spin coated on the ITO/Ag/ITO reflective anode electrode layer at 4,000 rpm for 60 s, followed by annealing at 150 °C for 30 min in a glovebox. TFB (10 mg ml^−1^ in chlorobenzene, American Dye Sources) is spin coated at 3,000 rpm for 60 s and annealed at 150 °C for 30 min. For the pixel array integration process, commercial Cd-free QD solutions (in octane, 12.5 mg ml^−1^ for the red and green devices and 17.5 mg ml^−1^ for the blue devices, Suzhou Xingshuo Nano) and commercial Cd-based QD solutions (Guangdong Poly Optoelectronics, 12.5 mg ml^−1^ in octane for all devices) are spin coated at 3,000 rpm onto donor substrates to achieve around 20-nm-thick QD layers.

The QDs are transferred onto the LTPS backplane via CATP and annealed at 80 °C for 10 min. For the ETL layer, a Zn_0.85_Mg_0.15_O nanocrystal (Guangdong Poly Optoelectronics, 25 mg ml^−1^ in butanol) layer is spin coated at 2,000 rpm and annealed at 80 °C for 10 min. A 150-nm-thick transparent IZO layer is sputtered at 50 W under a process pressure of 3.7 mtorr with Ar at 23 s.c.c.m. as the common cathode electrode in sequence to minimize damage to the underlying layers (Supplementary Fig. 43). Finally, the AM QD-LED display is encapsulated to shield oxygen and moisture, which could degrade the device layer, using ultraviolet (UV)-curable adhesive. The flat-band energy band levels of each device layer for ideal band alignment conditions are schematically illustrated in Supplementary Fig. 33b. The thicknesses of each transport layer are presented in Supplementary Fig. 33c–e, and the thicknesses of the QD layers for the emissive layer are shown in Supplementary Fig. 33f–h for Cd-free QDs and in Supplementary Fig. 33i–k for Cd-based QDs. The HAADF-STEM images were obtained using a Thermo Fisher Scientific SPECTRA 200 STEM device at 200 kV, at 50–150 pA, with a convergence semi-angle of 29.9 mrad. The lamella samples were prepared by a Helios Nanolab (FEI) scanning electron microscopy/focused ion beam.

### EL QD-LED devices for performance analysis

Device structure of ITO/PEDOT:PSS/TFB HTL/QDs/ZnMgO/Al is used for the red, green and blue EL QD devices (Fig. 3). Patterned 100-nm ITO glass with an active area of 3.0 mm × 1.5 mm (Ossila) is used as a substrate. All other transport layers are fabricated with process conditions identical to those used for the AM EL QD displays without patterning. All the fabrication steps are carried out in an inert atmosphere, except for the PEDOT:PSS deposition, which is performed in ambient conditions. Al (150 nm) is then thermally evaporated as the cathode electrodes under high-vacuum conditions and annealed at 100 °C for 10 min in a nitrogen atmosphere. The devices are encapsulated in a glovebox using UV-curable adhesives. SC devices are also fabricated for comparison under identical conditions.

### Fabrication of flexible EL QD-LED

For the flexible ZnTeSe blue EL QD device, a 130-nm ITO polyethylene terephthalate film is used as the substrate. The annealing temperature of PEDOT:PSS and TFB is modified to 120 °C (from 150 °C) due to the low glass transition temperature of the polyethylene terephthalate substrate. ZnTeSe QDs are patterned as 57 µm × 38 µm dots over an area of 4.0 cm × 4.0 cm using CATP. Zn_0.85_Mg_0.15_O nanocrystals (in butanol, 25 mg ml^−1^) are spin coated onto the QD layers at 2,000 rpm and annealed at 80 °C for 10 min. The flexible devices are encapsulated by SC UV-curable adhesives at 1,500 rpm in the glovebox.

### Device characterization

PLQYs are measured using a Quantaurus-QY Plus UV-NIR Absolute Photoluminescence Quantum Yield Spectrometer (Model C13534, Hamamatsu) with an excitation wavelength of 370 nm. The electro-optical properties, including EL spectra, current density–voltage–luminescence (*J*–*V*–*L*) characteristics, EQE–voltage curves and device lifetimes are measured using a photonic multichannel analyser PMA-12 (Hamamatsu Photonics) with a Keithley 2400 source meter for precise voltage and current control. The *T*_50_ value at 1,000 cd m^−2^ for the devices was extrapolated using an experimental fitting curve, *y* = exp(–*Ax*^*B*^). The *T*_95_ value at 100 cd m^−2^ was derived from the measured *T*_95_ at 10,870 cd m^−2^ by applying an acceleration factor of 1.8 (ref. ^23^). The thicknesses of all the films are measured using an atomic force microscope (Icon, Bruker). The refractive index was measured using spectroscopic ellipsometry (Park Systems).

## Supplementary information

Supplementary Information Supplementary Notes 1–11, Figs. 1–43, Table 1, Methods and references.

Supplementary Code 1 Python code, ReadMe.txt, bitmap images and data text files.

## Source data

Source Data Fig. 3 QD-LED device performance data.

Source Data Fig. 4 QD-LED display performance data.

## Data Availability

The data that support the findings of this study are available from the corresponding authors on reasonable request. Source data are provided with this paper.

## Code availability

Source codes used for the image analysis are provided in Supplementary Code 1.
