# Supplementary material for: A cracking-assisted transfer printing technology for high-resolution quantum dot light-emitting diode displays
Source: Nat Electron. 2026 Jul 17;9(9):1046–57. doi: 10.1038/s41928-026-01670-9 (PMC13597301; doi:10.1038/s41928-026-01670-9)
Supplement: Supplementary file 1 [file 41928_2026_1670_MOESM1_ESM.pdf]

nature electronics

Supplementary information

https://doi.org/10.1038/s41928-026-01670-9

A cracking-assisted transfer printing technology for high-resolution quantum dot light-emitting diode displays

In the format provided by the authors and unedited

Table of Contents

Supplementary Methods 2

Supplementary Notes 5

Supplementary Figures 26

Supplementary Table 69

Supplementary References 70

Supplementary Methods

Donor substrate preparation for CATP

Si substrates are cleaned by ultrasonication process in acetone and IPA. After 15 minutes of ultraviolet-ozone treatment, Si substrates are immersed in an anhydrous hexane solution mixed with octadecyltrichlorosilane (ODTS; ODTS : hexane=3:1000 volume ratio, Sigma Aldrich) for 12 hours, to form a self-assembled monolayer (SAM) on donor substrates. The SAM-treated donor substrates are cleaned by ultrasonication in hexane and chloroform to remove unassembled ODTS molecules.

Preparation of PDMS stamps for CATP

The master templates for defining the micro-bumps of the PDMS stamps are prepared on a 4-inch silicon (Si) wafer by photolithography using a negative photoresist (SU-8 TF 6010, Kayaku Advanced Materials, Inc.) and a developer (SU-8 developer, Kayaku Advanced Materials, Inc.) to form 10 μm thick micro-bump for ordinary resolution down to 5 μm pixel. For higher resolution, SU-8 TF 6002 with low viscosity is employed for a 2 μm micro-bump structure. A mixture of 10:1 (base:agent) PDMS prepolymer (Dow Corning) is poured onto the master template for replication. To prevent the shrinkage of the PDMS stamp during the curing step, the PDMS prepolymer is cured for 100 hours at room temperature.

Electron-beam (E-beam) lithography for nanoscale geometry

Ultra-high-resolution mould by E-beam lithography is utilised for nanometre scale pixel patterning by intaglio TP and CATP. Nanopatterned polymethyl methacrylate (PMMA) master moulds are fabricated via E-beam lithography (Raith EBPG 5200). For E-beam patterning

within a few hundred-nanometre scale, 495 PMMA A8 resist is spin-coated at 4,000 revolutions per minute (rpm) and baked at 180 °C for 60 seconds to achieve 500 nm thickness. The PMMA resist is then exposed to an E-beam with a 100 keV energy and a 3 nA beam current, with the 650 μC cm^{-2} exposure dose. The exposed PMMA layer is developed in a methyl isobutyl ketone (MIBK) / isopropyl alcohol (IPA) solution at a 1:3 ratio for 30 seconds. In order to achieve higher resolution down to 100 nm stripe geometry, a thinner PMMA resist of 200 nm thickness is used. The 495 PMMA A8 resist is diluted with anisole (495 PMMA A8 / anisole ratio of 2:1) and processed under identical E-beam conditions on the above.

Precise Alignment for AM QD-LED display

The entire alignment process was performed using a customised transfer printer from Seoul Engineering Co., Ltd. (Supplementary Fig. 30a), comprising an upper stage and moving stages with XYZ translation, as well as a three-axis tilting system. In addition, a left and right-movable 7× optical microscope was installed above the system for alignment. The PDMS stamp and LTPS substrate were aligned using alignment keys patterned on both components. Initially, the PDMS stamp was placed on a glass carrier and held in place by vacuum on the upper stage, while the LTPS substrate was positioned and held by vacuum on the moving stage. The microscope was first focused on the LTPS alignment key. Subsequently, the PDMS stamp was lowered to a position near the depth of field of the 7× microscope, ensuring that it did not contact the LTPS. Alignment was then achieved by adjusting the LTPS position in the x–y directions and rotating the stage until the alignment keys on the PDMS and LTPS were matched within the microscope's depth of field. Once alignment was complete, the PDMS stamp and LTPS substrate were brought into contact under a load of 0.1 kg, and the vacuum was released to ensure full contact. Supplementary Fig. 30b,c shows the PDMS and LTPS successfully

aligned at the alignment key region. Finally, the PDMS stamp was peeled off using the transfer printer at a slow velocity of 1 mm s^{-1}, allowing the QDs to transfer onto the substrate.

Supplementary Note 1

<u>Modelling analytical tensile stress at QD layer</u>

The tensile stress at the edges of the QD layer in contact with the PDMS bump is derived analytically for both conventional TP and CATP during the pick-up process. For a square PDMS bump with side length L, the force applied to the PDMS bump is $F = P_0 \times L^2$ (Fig. 1d). This force equilibrates with the force by the tensile stress σ across the cross-section at the contact edges of the QD layer, $F = \sigma \times t_{QD} \times l$, where t_{QD} and l represent the effective thickness of the QD layer and length of the edges in contact with the PDMS bump, respectively. Therefore, the analytical tensile stress developed across the cross-section at the contact edge of the QD layer is given by $\sigma = P_0 \times L^2/(t_{QD} \times l)$. In the conventional TP where QDs are coated over the entire donor surface, the tensile stress is given by $\sigma = P_0 \times L/(4t_{QD})$ because four edges of the QD layer are in contact with the PDMS bump ($l = 4L$). In contrast, in the CATP, since only two edges are in contact with the PDMS bump ($l = 2L$), the tensile stress is $\sigma = P_0 \times L/(2t_{QD})$ which is two times higher than that of the conventional TP.

During the pick-up step, the viscoelastic square PDMS stamp with size L is pulled using an impact force generated by an instantaneous pulling pressure P_0. As a result, the velocity-dependent adhesion force F_{pq} rapidly increases, reaching a level sufficient to detach the QDs from the SAM layer ($F_{pq} > F_{qs}$) (Fig. 1e). This leads to the QDs being lifted off from the donor substrate, and the entire pulling force acting on the PDMS is balanced by the force exerted along the pixel edge of the QD layer. This force balance creates the tensile stress σ at the cross-sections of the QD layer along the pixel edges. Meanwhile, the intermolecular cohesive force between QDs (F_{qq}) determines the critical fracture stress σ_{cf} required to break the bond between QDs. Therefore, when σ exceeds σ_{cf}, the bonds between the QDs along the pixel edge breaks, allowing the QDs to be cleanly picked up from the donor with the desired pixel shape.

Simulation of von Mises stress distribution in QD layer during the conventional TP and CATP process

During the pick-up process, the von Mises stress distribution in the QD layer and PDMS stamp is simulated using COMSOL software with a three-dimensional finite element method (Fig. 1g and Supplementary Fig. 9). The simulation is initiated with a rigid donor substrate with 300 nm (x) × 300 nm (y) × 100 nm (z) dimensions. A 20 nm thick QD layer on the donor substrate is assumed to be an elastic layer with Young's modulus of 250 kPa and Poisson's ratio of 0.3. A 50 nm thick PDMS bump with 100 nm × 100 nm dimension is attached to the QD layer. To set up the initial detachment, a very thin gap of 0.1 nm between the QD layer and the donor substrate is introduced. The top face of the PDMS stamp is considered to be a rigid surface with a boundary load P_0 of 10^5 N m^{-2} in a vertical pulling direction (Supplementary Fig. 9a,b).

The von Mises stress distribution in the QD layer of the conventional TP and the CATP processes are compared (Supplementary Fig. 9c,d). For the conventional TP pick-up process with the non-cracking step, the QD layer is assumed to be fully coated on the donor surface (Supplementary Fig. 9a). In the conventional TP, a von Mises stress of approximately 2.0×10^5 N m^{-2} is generated at the contact edge of the QD layer, with a 17 nm gap between the QD pixel layer and the substrate (Supplementary Fig. 9c).

For the simulation of the CATP pick-up process, a stripe pattern of the QD layer with 100 nm × 300 nm dimensions is considered to represent the remaining QD layer after cracking (Supplementary Fig. 9b). In the CATP process, von Mises stress of around 4.5×10^5 N m^{-2} is generated at the contact edge of the QD layer with a larger gap value of 27 nm (Supplementary Fig. 9d). According to Hook's law, this tensile stress σ is proportional to the applied strain (ε) in the QD layer, where $\sigma = E_{QD}\times\varepsilon_{QD}$, with E_{QD} representing the Young's modulus of the QD material. By eliminating F_{qq}, the CATP method increases local strain, which in turn amplifies the stress, resulting in more effective fractures along the QD edges.

Supplementary Fig. 9e,f show the distribution of tensile stress within the QD layer according to PDMS bump sizes in conventional TP, as simulated by a solid mechanics model. Here, PDMS bump sizes are considered to have 50 nm × 50 nm and 200 nm × 200 nm to examine the effect of PDMS bump size. As shown in the figure, the 50 nm × 50 nm PDMS bump results in lower tensile stress within the QD layer, compared with the 200 nm × 200 nm PDMS bump. Supplementary Fig. 9g compares the analytical and simulated tensile stresses at the edge cross-sections of the QD layer for various PDMS bump sizes in the conventional TP process. The size-dependent tensile stress for different pattern sizes in the CATP is also presented in the figure. As the PDMS bump size decreases, the tensile stress at the QD layer becomes smaller in both the conventional TP and CATP processes. However, the CATP exhibits twice times higher tensile stress compared with that of the conventional method, exerting stronger stress to fracture the QD layer. This facilitates the easy pick-up of QDs through the cracking process.

Supplementary Note 2

Influence of slow peeling velocity on QD transfer

Supplementary Figure 4 shows that slow peeling during the transfer process enables QD transfer by ensuring that the adhesion between the stamp and the target substrate exceeds that between the stamp and the QDs. The viscoelastic nature of PDMS causes its interfacial adhesion to decrease sharply with decreasing peel velocity as seen in Supplementary Fig. 4b. Thus, the peel-off velocity is kept below 1 mm s^{-1}, and the PDMS–QD adhesion becomes sufficiently weak to allow the QDs to transfer on the target substrate.

To experimentally demonstrate that slow peeling reduces PDMS–QD adhesion to a level that enables transfer even onto a low surface energy SAM substrate, a ~50 nm Cd-based green QD film was transferred onto ODTS-treated SAMs. First, we pick up the Cd-based green QD films as shown in Supplementary Fig. 4c. Under slow-peeling conditions, the QD film was successfully transferred onto the ODTS-treated substrate (Supplementary Fig. 4d), with no detectable residue remaining on the stamp (Supplementary Fig. 4e). This confirms that, at low peeling velocities, the PDMS–QD adhesion force (F_{pq}) becomes smaller than both the QD–ODTS adhesion force (F_{qs}) and the interlayer QD–QD cohesive force($F_{qq\_interlayer}$).

Multilayer QD pickup mechanism in CATP

To elucidate the multilayer QD pickup and transfer mechanism in CATP, we separately consider two QD–QD cohesive forces: the interlayer QD–QD interaction ($F_{qq\_interlayer}$) and the lateral edge QD–QD interaction (F_{qq_edge}) as illustrated schematically in Supplementary Fig. 13a. In addition, the competing adhesion forces between the PDMS stamp and the QD film (F_{pq}) and between the QD film and the ODTS SAM substrate (F_{qs}) are taken into account.

We first experimentally confirm that the interlayer QD–QD cohesive force exceeds the PDMS–QD adhesion force during fast pick-up ($F_{qq\_interlayer} > F_{pq}$ under fast peeling conditions).

Specifically, using a large-area PDMS stamp at a peeling velocity of 500 mm s^{-1}, we attempted to pick up QDs from a 50 nm-thick Cd-based green QD film coated on an ozone-treated Si substrate, as shown in Supplementary Fig. 13b,c. While successful QD pickup is observed from ODTS SAM-treated substrates, no pickup occurs from the ozone-treated Si substrate to the PDMS stamp. This result indicates that both the adhesion between the QD film and the ozone-treated Si substrate and the interlayer QD–QD cohesion exceed the PDMS–QD adhesion force under fast peeling conditions.

Based on the force hierarchy $F_{qq\_interlayer} > F_{pq}$(fast pick-up) > F_{pq}(slow transfer) the following sequence occurs during CATP pickup and transfer. During fast pick-up (Supplementary Fig. 13d), the QD film is first cracked at the patterned lateral edges, which effectively reduces the lateral edge cohesion (F_{qq_edge}). As a result, fracture initiates preferentially at the designed lateral interfaces, while internal fracture between QD interlayers is suppressed because the interlayer QD–QD cohesion is larger than the PDMS–QD adhesion force. This enables reliable pickup of multilayer QD films without disrupting their internal integrity. Subsequently, during the slow transfer step (Supplementary Fig. 13e), the PDMS–QD adhesion force decreases sufficiently to allow the QD film to be released onto the target substrate, while avoiding interlayer delamination within the multilayer QD film.

Supplementary Note 3

Tens-of-micrometre-scale QD patterns

Supplementary Figure 14 presents the results for QD patterns with lateral dimensions on the order of tens of micrometres. Supplementary Figure 14a–c shows top-view optical EL images of red, green, and blue QD pattern arrays with sizes of 37 × 46 μm² (red), 37 × 47 μm² (green), and 37 × 56 μm² (blue). Supplementary Figure 14d–f displays 3D bar plots of the relative pattern areas extracted from 25 samples of red, green, and blue, exhibiting highly concentrated distributions without noticeable outliers. Supplementary Figure 14g–i shows the corresponding histograms, fitted with Gaussian functions, demonstrating narrow spreads with standard deviations of 0.019, 0.014, and 0.020 for red, green, and blue patterns, respectively. These results confirm that CATP achieves highly uniform and stable pattern formation at the display-relevant tens-of-micrometre scale, with area variation kept below 2%.

Micrometre-scale QD patterns

Supplementary Figure 15 examines the patterning characteristics at the 2 μm scale. Supplementary Figure 15a–c presents top-view optical EL images of red, green, and blue QD patterns, where individual features appear visually less distinct due to the reduced physical size and the diffraction-limited resolution of optical microscopy. Supplementary Figure 15d–f shows 3D bar plots of relative pixel areas extracted from 100 samples (N = 10) of red, green, and blue patterns, revealing broader distributions than in Supplementary Fig. 15 as expected for near-diffraction-scale features. Supplementary Figure 15g–i provides Gaussian-fitted histograms showing standard deviations of 0.081 for red, 0.068 for green, and 0.079 for blue patterns. Although these values indicate increased variability relative to the tens-micrometre

regime, the CATP process nonetheless succeeds in consistently transferring all patterns across the entire array, demonstrating its robustness even at this challenging 2 μm scale.

Submicrometre-scale QD patterns

Supplementary Figure 16 extends the analysis to the sub-micrometre regime, focusing on QD patterns measuring 0.6 × 0.9 μm^2. Supplementary Figure 16a shows a top-view SEM image confirming the successful formation of well-defined sub-micrometre QD pixel arrays. Supplementary Figure 16b presents the 3D bar plot of relative pixel areas obtained from 25 samples (N = 5), which visually exhibits a somewhat wider distribution than the tens-of-micrometre case but notably narrower than the 2 μm case. Supplementary Figure 16c provides the Gaussian-fitted histogram with a standard deviation of 0.051, revealing that CATP maintains appreciable pattern fidelity even at the nanoscale. Importantly, the observed variation is smaller than at the 2 μm scale, indicating that pattern fidelity does not deteriorate monotonically with decreasing feature size. Supplementary Figure 16d further presents the AFM height-profile analysis, confirming well-defined pixel edges and uniform feature heights, thereby further supporting the nanoscale pattern fidelity achieved by the CATP process.

Supplementary Note 4

<u>Formation of irregular QD pattern by CATP</u>

Supplementary Figure 17a–c illustrate the conceptual steps of the CATP process using the example of a circular pixel. In the first step (Supplementary Fig. 17a), the cracking stamp removes two opposite edges of the uniformly coated QD film, producing stripe-like QD segments on the donor substrate. This step is essential because it reduces the dominant lateral cohesive forces at those edges, leaving only two remaining edges that can subsequently be fractured in a controlled fashion. When the pick-up stamp (Supplementary Fig. 17b), is brought into contact with the cracked stripes, its circular micro-bump geometry defines the mechanical boundary along which the fracture propagates. As a result, the pick-up step selectively detaches only the circular portions of the initially stripe-shaped QD region. This sequence leads to the outcome illustrated in Supplementary Fig. 17c, where the circular QD pixels are cleanly lifted onto the PDMS stamp while the surrounding material remains on the donor.

Supplementary Figure 17d–f provide experimental verification corresponding directly to this conceptual sequence. After the cracking step (Supplementary Fig. 17d), only parallel QD stripes remain on the donor substrate. Following the pick-up step reveals that circular regions have been selectively removed, leaving behind the complementary cracked pattern (Supplementary Fig. 17e). Finally, Supplementary Fig. 17f confirms that these circular pixels are successfully transferred to the target substrate, demonstrating that the fracture-guided mechanism of CATP faithfully reproduces the intended circular geometry. These three images collectively demonstrate that the transition from a continuous QD film to circular pixel arrays proceeds exactly as predicted by the schematic process. Beyond circular pixels, Supplementary Fig. 17g–i further demonstrate that CATP can reliably generate much more complex geometries. Using appropriately shaped pick-up bumps, we produced star-shaped (Supplementary Fig. 17g), triangular (Supplementary Fig. 17h), and hexagonal (Supplementary Fig. 17i) pixel arrays with

high fidelity. These results confirm that the CATP mechanism is inherently generalisable: the cracking step always produces stripe-like QD segments with two free edges, and the pick-up step then defines the final pixel boundary according to the shape of the micro-bump. As long as the intended geometry is encoded into the pick-up stamp, the fracture naturally follows the mechanically weakest path defined by the stamp contour.

Supplementary Note 5

Preparation of QD materials

Commercial InP/ZnS red, green QDs and CdSe/ZnS red, green, and blue QDs are purchased from Suzhou Xingshuo Nano. ZnTeSe/ZnSe/ZnS core/shell blue QDs are synthesised as detailed previously[S25]. In brief, a three-neck flask containing 2 mmol zinc acetate ($Zn(Ac)_2$), 2 mL oleic acid (OA), and 15 mL octadecene (ODE) is degassed at 120 °C and heated to 210 °C under a nitrogen atmosphere. Subsequently, 1.5 ml of 2 M Se precursor and 0.75 mL of 0.047 M Te precursor dissolved in tri-*n*-octylphosphine (TOP) solution are added into the flask. The core formation process is maintained at 210°C for 30 minutes, followed by 1 hour at 300 °C. Then, 20 mL of $Zn(OA)_2$ solution and 5.0 mL of 1.2 M Se precursor dissolved in TOP are injected into the flask using a precision syringe pump to grow the ZnSe shell. The resulting ZnTeSe/ZnSe core/shell QDs are then extracted from a 20 mL QD solution by ethanol precipitation and redissolved in 9 mL hexane. Subsequently, 2 mmol $Zn(Ac)_2$, 4 mmol 1-hexadecylamine (HAD), 2 mL OA, and 20 mL trioctylamine (TOA) are degassed at 120 °C for 10 minutes, followed by nitrogen refilling. Then, 3 mL of the ZnTeSe/ZnSe/ZnS QDs in hexane is added into the flask, and the mixture is heated to 330 °C. The reaction is initiated with the addition of 1.5 mL of 1.2 M S in TOP and 6 mL of $Zn(OA)_2$, continuing for 1 hour to form the outer shell. The synthesized QDs are repeatedly purified via the precipitation (acetone)/redispersion (hexane) method before redispersing in octane for EL QD-LED device fabrication.

Synthesis of $Zn_{0.85}Mg_{0.15}O$ nanocrystals for ETL

Colloidal ZnMgO nanoparticles are synthesized by dropwise adding 10 mL ethanol solution of a 0.5 M tetramethylammonium hydroxide (TMAH) into a 30 mL dimethyl sulfoxide (DMSO)

solution of zinc acetate dihydrate (0.85 M) and magnesium acetate tetrahydrate (0.15 M). The mixture is precipitated from the solution by adding acetone and then dispersed in butanol.

Supplementary Note 6

LTPS TFT backplane

Supplementary Figure 18 provides details of the TFT backplane used in this study. The active area of the display screen in the TFT backplane is 1.41-inch in diagonal size (Supplementary Fig. 18a). The TFT backplane employs a pentile RGB pixel layout (Supplementary Fig. 17b). The cross-sectional architecture of the TFT backplane is schematically illustrated in Supplementary Fig. 18c. The TFT backplane, incorporating a conventional coplanar LTPS TFT architecture, is fabricated using an eight-photomask process sequence. First, a SiO_2/SiN_x buffer layer is deposited on a glass substrate via a sputtering process, followed by the formation of an LTPS layer for active channel through excimer laser annealing. Then, a SiN_x layer is deposited as the gate insulator 1 via plasma-enhanced chemical vapour deposition (PECVD). A molybdenum (Mo) layer is deposited to serve as the set of gate line 1 and source/drain electrodes via the sputtering process. Subsequent layers included a SiN_x as the gate insulator 2, followed by another Mo layer for the set of gate line 2. A SiN_x/SiO_2 interlayer is deposited as a passivation layer via PECVD. For the data lines, a tri-layer of titanium (Ti)/Al/Ti is deposited via a sputtering process. A planarization layer is then formed on top of the data line layer. After planarization, an ITO/silver (Ag)/ITO stack is patterned via the sputtering and photolithography process as a reflective anode contact electrode for driving QD-LED pixel devices. After patterning the anode electrode layer, 1.5 μm deep bank structures, designed to avoid the strong electric field at the edge of the pixel electrode, are formed with a pixel-defining layer using photolithography.

The transfer characteristic curve of the driving TFT is plotted in Supplementary Fig. 18d. The dimensions of the active channel for the driving TFT are 3.5 μm in width and 40 μm in length. The gate threshold voltage of the driving TFT is determined to be -2.29 V. The channel mobility

and the on/off ratio are measured to be 106.8 $cm^2\ V^{-1}\ s^{-1}$ and 4.18×10^5, respectively, under a drain voltage of -10 V.

Supplementary Note 7

Topological match (conformality) of the CATP on the bank-structured TFT backplane without air-bubble entrapment

The CATP technique, employing a micro-bump PDMS stamp, enables selective pixel formation due to the topological match of the micro-bump PDMS stamps with the bank structures on TFT backplanes. Figure 2a shows the micro-bump PDMS with a 1.41-inch size in diagonal on the TFT backplane, demonstrating the air-bubble entrapment-free QD pattering for large-area processing by CATP. To compare the QD-TFT backplane interaction between CATP and intaglio processes during the placing step, we performed a finite-element simulation to more clearly elucidate the mechanical behaviour of micro-bumped PDMS stamps on bank-structured emission areas. Figure 2e presents a comparison of the deformation profiles of a micro-bumped PDMS stamp (CATP) and a flat PDMS stamp (intaglio) subjected to the same applied pressure. The micro-bumped stamp, designed with a lateral margin slightly larger than the emissive region, conforms to the bank-defined emissive area and ensures firm contact within the emissive region (Fig. 2e, left). In contrast, the planar stamp fails to reach the emission area under identical loading conditions (Fig. 2e, right). These simulations confirm that the micro-bump geometry provides superior vertical compliance, enabling the stamp–QD interaction to reliably dominate over the QD–backplane adhesion during the placing step. This behaviour directly explains the higher placement yield achieved in CATP, ensuring full contact of the pixelated QD layer within the pixel area on the non-planar surface, as evidenced in Fig. 2f.

Full-colour integration of the QD pixels on the TFT backplane by CATP

Supplementary Figure 27 schematically outlines the integration of full-colour red, green, and blue QD pixels onto the TFT backplane. In this study, the green, blue, and red QD pixels are

integrated in sequence using a CATP technique. The red QD layers on the donor substrate are patterned into stripes by the cracking steps as shown in Supplementary Fig. 27a-c. Utilizing these stripe-patterned donor substrates, the red QDs are picked up (Supplementary Fig. 27a,b) and transferred to the designated location on the target TFT backplane as illustrated in Supplementary Fig. 27c. The full-colour integration steps and PL images of the pixelated QD layers for the green, blue, and red pixel processes on the TFT backplane are demonstrated in Supplementary Fig. 27d-i.

Supplementary Note 8

QD pixel patterning capability and quality by intaglio TP

Supplementary Fig. 19a describes the process of intaglio TP that begins with the lifting of the entire QD layer using a flat PDMS from the donor substrate. The QDs on the flat stamp are then patterned by removing all QDs except for the designed pattern using an intaglio trench, enabling the transfer printing of the desired QD pattern onto the target substrate. Intaglio TP process with nano-structured PMMA moulds by E-beam lithography is used for patterning stripe lines for the highest resolution of QD EL devices. A red QD EL device with a 600 nm width and 900 nm space stripe geometry is fabricated using intaglio TP with the emissive area of 3.0 mm × 1.5 mm (inset), exhibiting a luminance of 3,826 cd m^{-2} at 8 V (Supplementary Fig. 19b). Supplementary Fig. 19c shows the first successful QD EL device of 100 nm stripe geometry processed by intaglio TP process. The inset shows green QD EL devices with 300 μm × 150 μm emissive area at ultra-high-resolution with luminance of 3,123 cd m^{-2} at 8 V.

Full-colour AM EL QD displays by intaglio TP

A Nano-patterned EL QD-LED device in a small area is successfully demonstrated by intaglio TP. However, the flat PDMS stamp in large area application by the intaglio TP generates air-bubble entrapment between the stamp and the substrate due to the lack of the necessary paths for air to escape as shown in Supplementary Fig. 19d. In addition to air-bubble entrapment, LTPS TFT backplane based upon bank structure (Supplementary Fig. 19e). Supplementary Fig. 19f shows the unsuccessful pixelization by intaglio TP even for large pixels of 57 μm × 38 μm with 1.5 μm deep bank. Hence, a 0.5 μm deep bank structure is employed to facilitate the easier pixelization for full-colour AM EL QD display by the intaglio TP. As a result, Cd-free and Cd-based RGB EL QD display images on 1.41-inch LTPS backplane by intaglio TP are

demonstrated in Supplementary Fig. 19g,h. However, there are still serious air-bubble entrapment phenomena by intaglio TP which causes strong colour Mura. Therefore, incomplete pixelization by intaglio TP is a serious issue due to air-bubble entrapment, especially for a large-area pixelation process.

Supplementary Note 9

Detailed design of QD patterns

Supplementary Figure 28 shows the spatial arrangement of the red, green, and blue subpixels within a 74.49 µm × 74.49 µm pixel on the LTPS TFT backplane. The emission areas of each subpixel are indicated in white, following a pentile architecture (Supplementary Fig. 28a). To accommodate process-induced misalignment throughout CATP, the QD patterns were intentionally designed to be larger than the emissive areas while remaining confined within the midpoint boundaries between neighbouring subpixels (Supplementary Fig. 28b). This ensures that each transferred QD film fully covers its target emission area even in the presence of several micrometres of placement variation, while avoiding undesired intrusion into adjacent subpixel regions. Based on this constraint, the final QD pattern sizes were set to 37.25 µm × 46.0 µm (red), 37.25 µm × 47.0 µm (green), and 37.25 µm × 56.0 µm (blue). These dimensions exceed the corresponding emissive areas by more than 20 µm in both lateral directions, providing a robust alignment tolerance. As a result, the total vertical span of the RGB stack becomes 149 µm (approximately twice the pixel pitch), while the horizontal width remains within the pixel pitch (74.49 µm).

Design rule for PDMS stamps

Supplementary Figure 29 presents the PDMS stamp design rules for the cracking and pick-up steps. Here, we used a blue subpixel as an example to describe the PDMS stamp design rule. In the pentile structure, the blue QD pattern is 37.25 µm wide and 56 µm tall, with a 36 µm vertical spacing between adjacent blue subpixels (Supplementary Fig. 29a). During the cracking step, the PDMS stamp incorporates horizontal stripe-shaped bumps that break the QD film into stripes without requiring any alignment. Because the donor substrate is uniformly

coated with a continuous QD layer at this stage, the stripe geometry ensures that the vertical spacing between PDMS bumps remains fixed at 36 μm, and the remaining QD stripes retain a width of 56 μm after cracking (Supplementary Fig. 29b).

In the pick-up step, selective retrieval of each stripe requires tight control of the bump geometry. Since the QD stripes extend across the entire donor substrate in the horizontal direction, horizontal alignment is not necessary. Accordingly, the horizontal width of the pick-up bump is set to 37.25 μm. Conversely, vertical alignment is critical to maintaining high pick-up yield; therefore, the vertical dimension of the pick-up bump is designed to be 3.0 μm larger than the 56 μm stripe width (i.e., +1.5 μm margin on each side) (Supplementary Fig. 29c). This expanded margin maximises the window for mechanical engagement between the PDMS bump and the QD stripe while preventing accidental pick-up of adjacent stripes. The resulting bump geometry reliably fractures the remaining QD edges during the pick-up step and enables transfer of the intended pattern onto the target TFT backplane with high fidelity.

Alignment Accuracy

The alignment accuracy was experimentally quantified by measuring the centre-to-centre positional deviation between the transferred QD patterns and their corresponding emissive areas on the LTPS TFT backplane. Supplementary Figure 31 shows that the red, green, and blue subpixels exhibited alignment offsets of (–2.9, –3.7) μm, (–4.9, –8.4) μm, and (–4.7, +1.2) μm, respectively, where each pair denotes the horizontal and vertical deviation. When averaged across all subpixels, the overall misalignment amounted to approximately (–3.8, –3.6) μm. This accuracy not only falls within the mechanical tolerance of our transfer-printing equipment but, more importantly, remains well below the alignment margin intentionally engineered into both the QD pattern and the PDMS stamp design.

Supplementary Note 10

Computational Characterisation of EL QD-LED devices

The EQE of QD-LED devices is characterized by a theoretical ABC model with respect to the optical properties and the recombination properties of QD layers. Optical properties include the light extraction efficiency (LEE) of the optical device configuration and the PLQY of the QD film η_{PLQY}. The recombination properties of the QD materials are represented by the SRH recombination rate A, the Langevin recombination strength B, and the Auger recombination probability C (ABC parameters). In the ABC model, the EQE and current density are described by the functions of the carrier density k at QDs as shown in equations (1) and (2).

$$\eta_{\mathrm{EXT}} = LEE \frac{\eta_{\mathrm{PLQY}} Bk}{A + Bk + Ck^2} \quad (1)$$

$$J = q t_{\mathrm{QD}} (Ak + Bk^2 + Ck^3) \quad (2)$$

Here, q is the proton charge and t_{QD} is the effective thickness of the QD film. In the computational device characterisation, LEE is set to be 0.20, and the thicknesses of red, green and blue Cd-free QD films are assumed to be 22.5 nm, 19.5 nm, and 15.0 nm from the experimental results in Supplementary Fig. 33f-h. To emulate the EQE-current density curves of the spin-coated red, green, and blue QD-LED devices, the A, B, and C parameters are set to be 1.39×10^{5} s^{-1}, 0.77×10^{-12} cm^{3} s^{-1}, and 1.99×10^{-30} cm^{6} s^{-1} for red, 2.03×10^{6} s^{-1}, 0.77×10^{-12} cm^{3} s^{-1}, and 5.37×10^{-30} cm^{6} s^{-1} for green, and 7.60×10^{4} s^{-1}, 0.77×10^{-12} cm^{3} s^{-1}, and 0.53×10^{-30} cm^{6} s^{-1} for blue. The η_{PLQY}'s for spin-coated red, green, and blue QD films are set to be 0.435, 0.226, and 0.209, and those for CATP-based red, green, and blue QD films are set to be 0.527, 0.338, and 0.245 from the experimental measurements in Fig. 3g.

Supplementary Note 11

Spectral comparison of Cd-free and Cd-based QDs

Supplementary Fig. 42 illustrates spectral characteristics and emission wavelength dependencies on core sizes for both Cd-free and Cd-based QDs. The emission wavelength is calculated using the effective mass approximation. Supplementary Fig. 42a,b outline the core-size dependent emission wavelengths for the Cd-free (InP for red/green, and ZnTeSe for blue) and Cd-based (CdSe for red, green, and blue) QDs. The peak wavelengths of InP/ZnS red and green, and ZnTeSe/ZnSe/ZnS blue QDs are 625 nm, 530 nm, and 460 nm, respectively, and those of Cd-based red, green, and blue QDs are 630 nm, 530 nm, and 470 nm. Combined with the FWHM of the QDs in Supplementary Fig. 42, the effective mass approximation model suggests that Cd-based QDs demonstrate a more uniform core size compared to Cd-free counterparts (InP and ZnTeSe). From the figure, it is presumed that Cd-based QDs offer superior core-size precision due to the maturity of the synthesis method. Therefore, a more stable process condition to enhance core-size controllability in Cd-free QD synthesis is required to achieve a wider colour gamut with the Cd-free QDs. Supplementary Fig. 42c-h provide experimental validation showing a strong relationship between prediction core size and the experimental observation of particle size in Cd-free and Cd-based QDs.

Supplementary Fig. 1: Progress chart of the current state-of-the-art technologies.

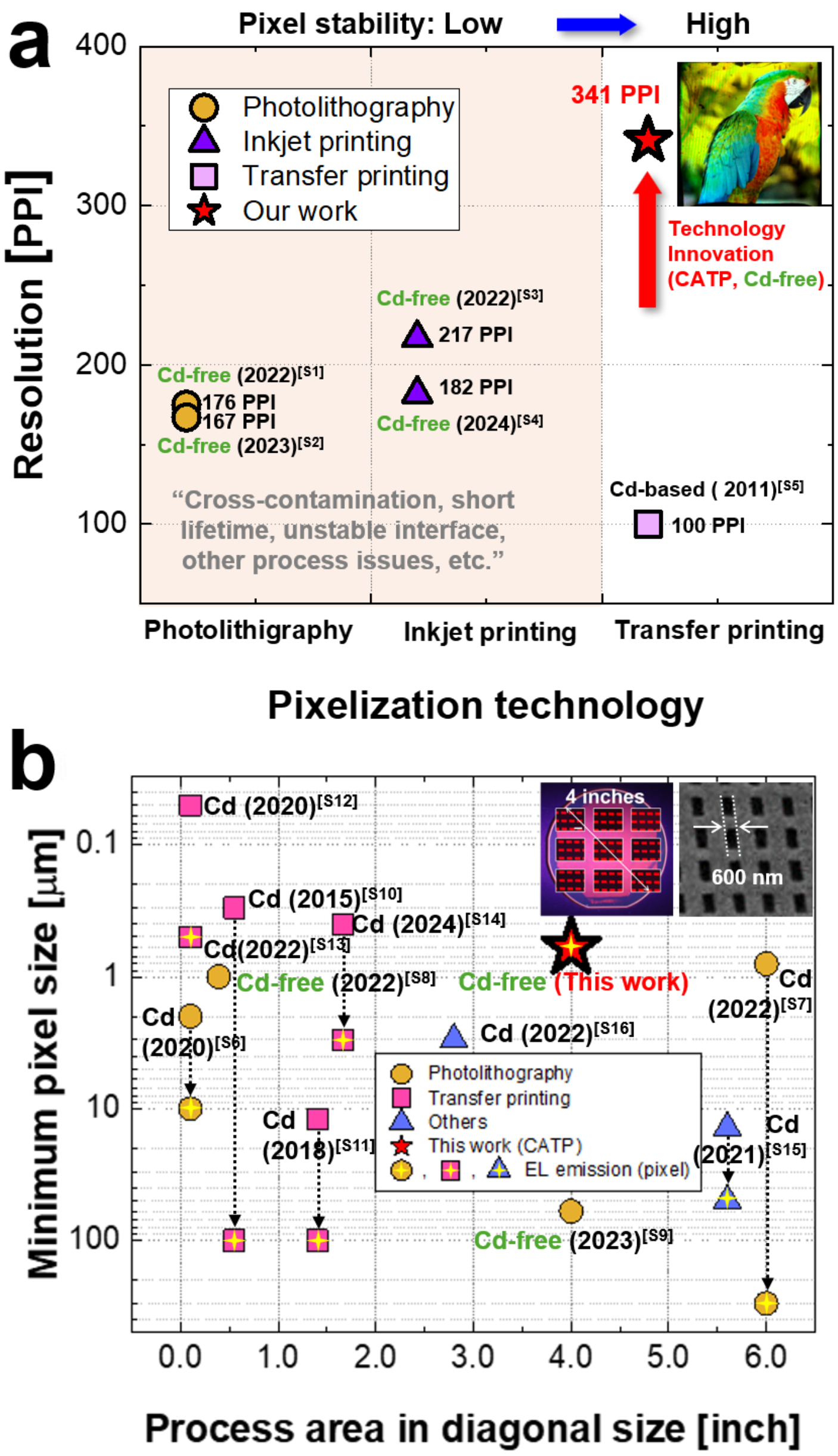

a, Progress on AM EL QD display technologies with respect to pixelization technology and display resolution. The displays by direct photolithography and inkjet printing are marked with yellow circles and blue triangles. Our achievement by CATP is marked with a red star. The display resolutions of the AM EL QD displays processed by the direct photolithography and the inkjet printing range from 167 PPI to 217 PPI, while the display resolution achieved by CATP is 341 PPI with the Cd-free QD materials (Copyright Joakim Lloyd Raboff / www.raboff.com). **b**, Progress on QD pixel patterning technologies with respect to the process area in diagonal size and the dot pixel size. The patterning processibility of other pixelization technologies is marked with yellow circles and blue triangles. The EL emissions with pixelated QDs are marked with a yellow cross. Our achievement with EL emission by CATP is also marked with a red star.

Supplementary Fig. 2: Cracking-assisted transfer printing fabrication process.

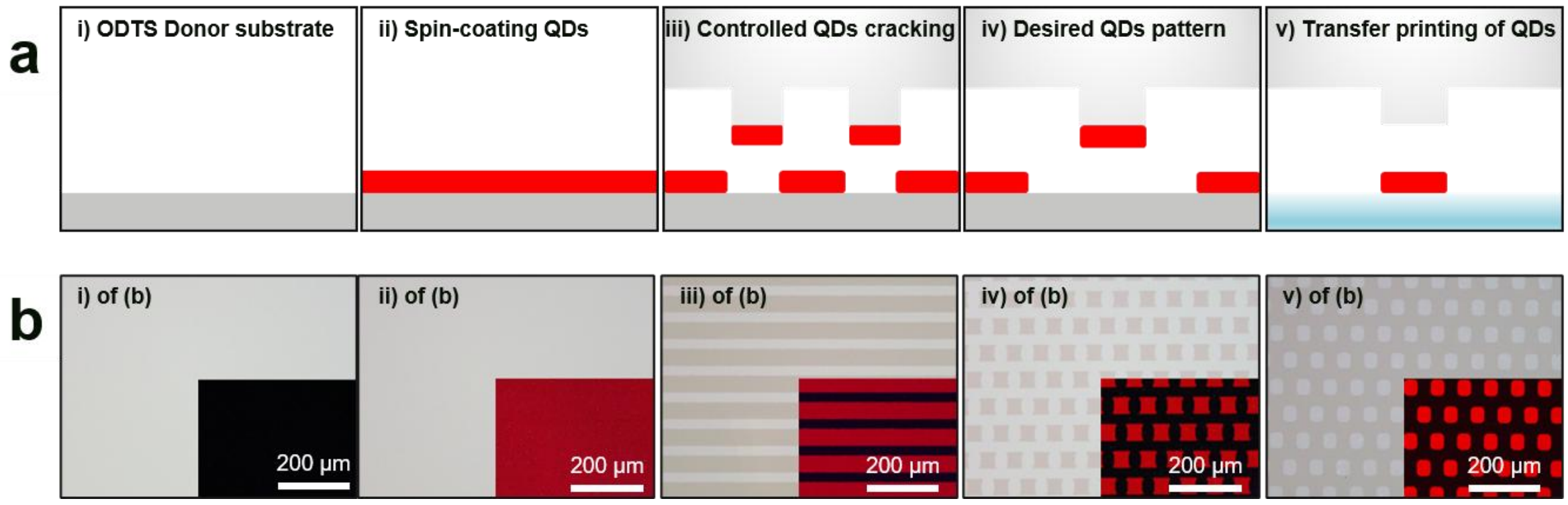

a, Cross-sectional schematic illustrations of the step-by-step cracking-assisted transfer printing technique for the step-by-step procedure. **b,** OM images (Scale bars, 200 µm) corresponding to the frames of (**a**). The inset shows a fluorescent OM image of the patterned QD film.

Supplementary Fig. 3: PL images of RGB QD films on SAM substrates.

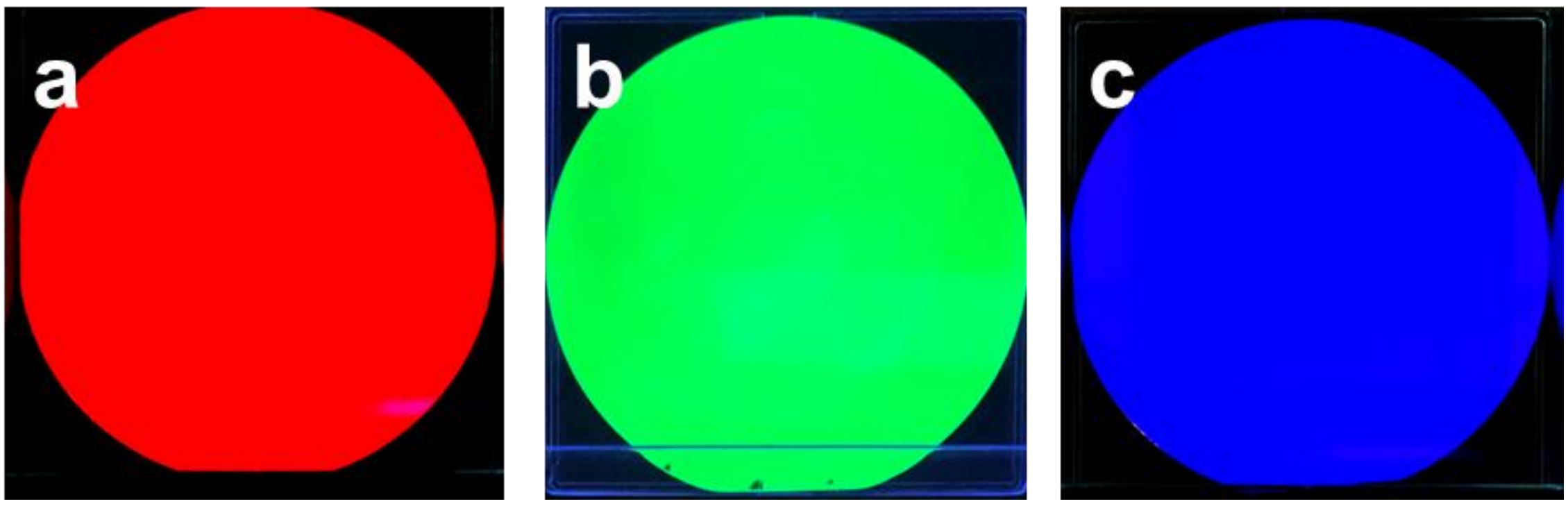

a-c, PL images of Cd-based red (a), green (b), and blue (c) QD films spin-coated on ODTS SAM-treated substrates, showing uniform emission across a 4-inch wafer.

Supplementary Fig. 4: Influence of slow peeling velocity on QD transfer.

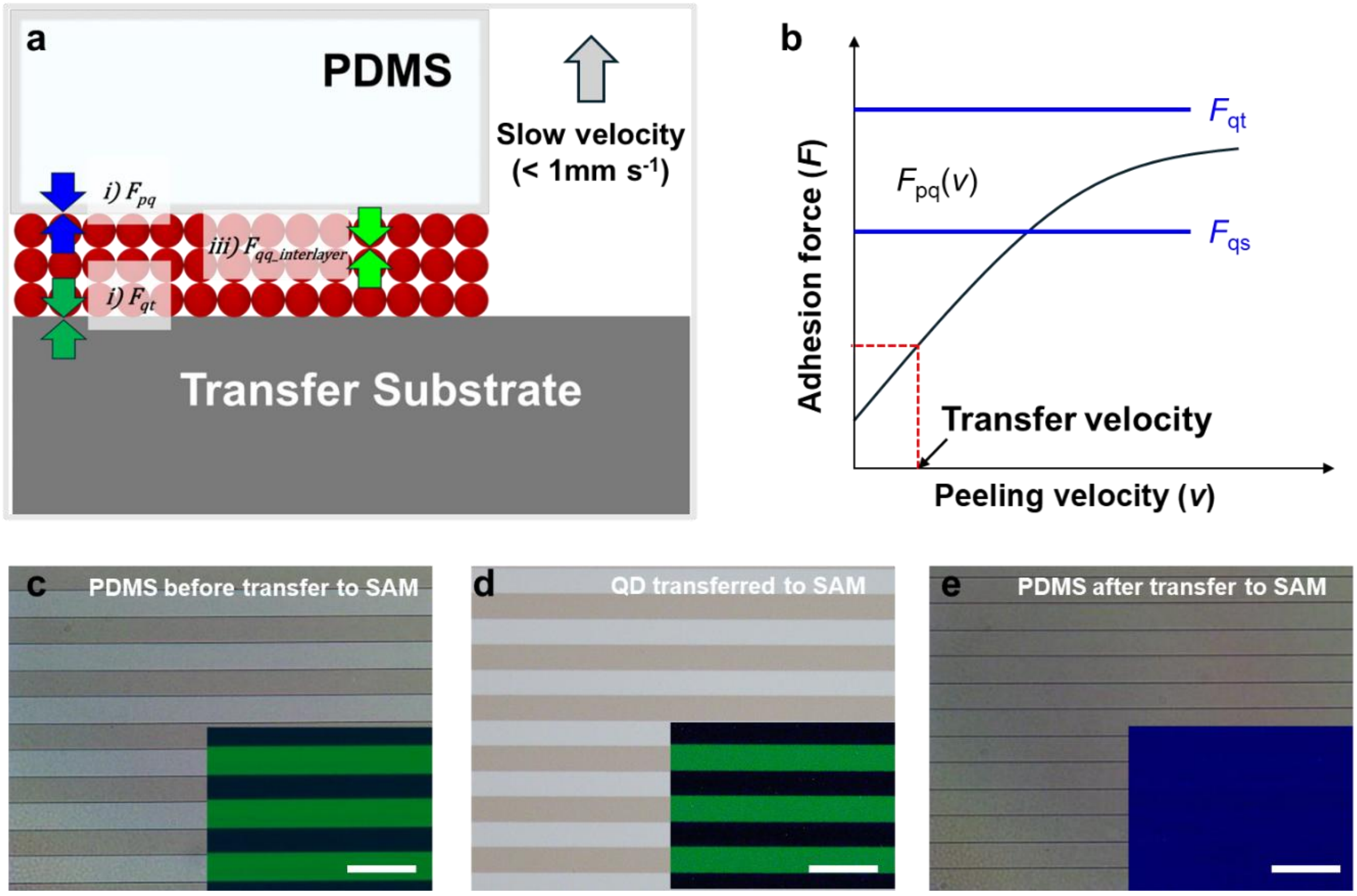

a, Schematic illustration of the competing adhesion and cohesive forces during QD transfer printing, including the PDMS–QD adhesion force (F_{pq}), the QD–substrate adhesion force (F_{qt}), and the interlayer QD–QD interlayer force($F_{qq\_interlayer}$). Slow peeling reduces F_{pq}, enabling release of the QD film. **b,** Force–peeling-velocity relationship illustrating the decrease in PDMS–QD adhesion under slow peeling. Under slow velocity F_{pq}, has a lower adhesion force than the QD-ODTS SAM adhesion (F_{ps}). **c-e,** Optical microscopy images showing a Cd-based green QD film before transfer on the PDMS stamp (c), successful transfer from PDMS onto an ODTS-treated SAM substrate under slow-peeling conditions (d), and OM image showing no residual QDs on the PDMS stamp after transfer (e). Scale bars: 100 µm.

Supplementary Fig. 5: Adhesion force related to competing fracture dynamics in kinetically controlled TP depending on QD pixel size.

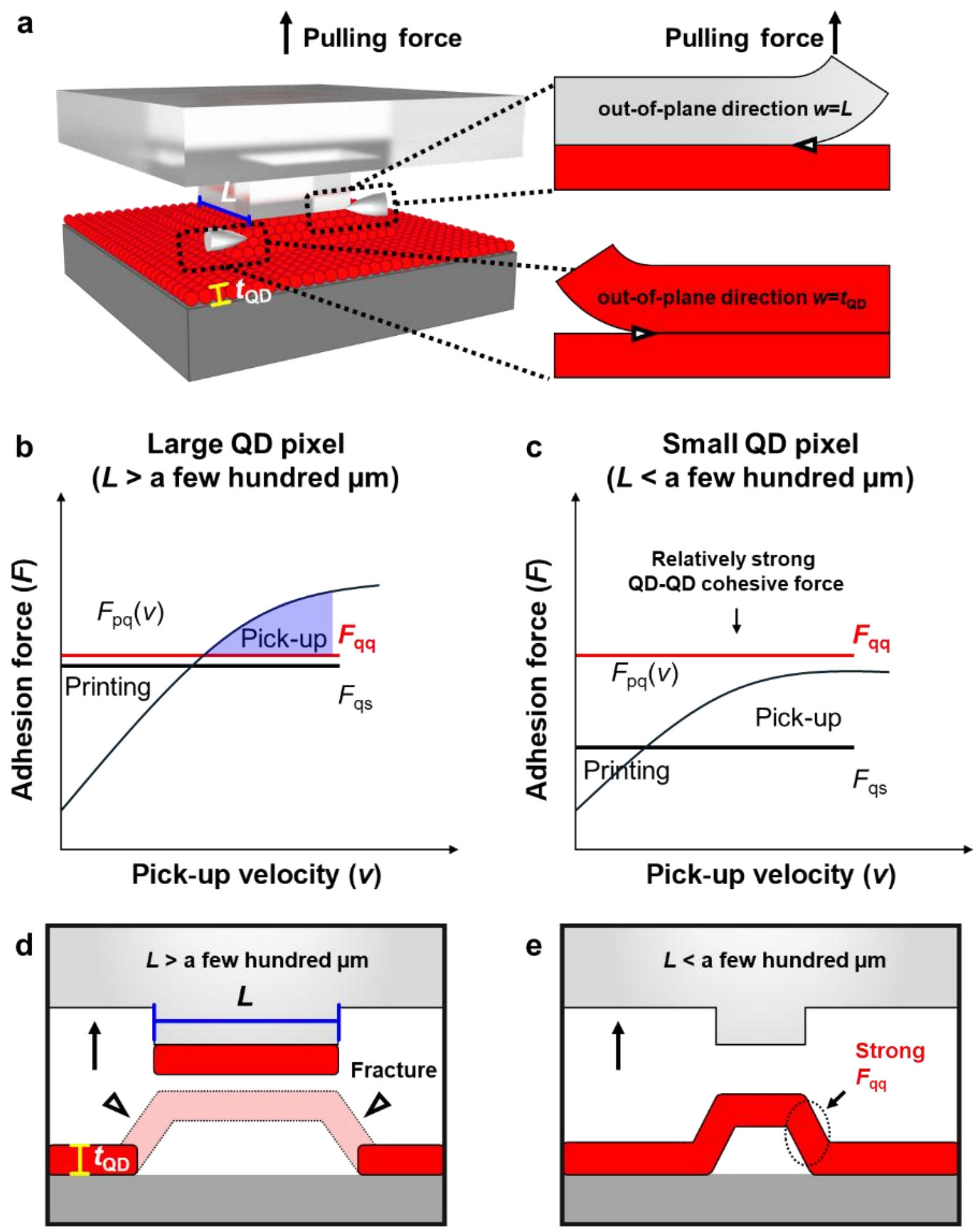

a, Schematic diagrams illustrating the separation of the PDMS/QD (right, top) and QD/QD (right, bottom) interfaces during the pick-up process. The white arrows indicate the direction of crack propagation. **b**,**c**, Schematic diagrams of the adhesion forces for the PDMS/QD interface, QD/substrate interface, and QD-QD interparticle cohesion of model QD TP experiments, consisting of a stamp, continuous QD film, and substrate. The intersection of the horizontal line with the monotonically increasing curve represents the critical pick-up velocity for kinetically controlled TP. The red and black horizontal lines correspond to the adhesion forces for QD-QD cohesion and the QD/substrate interface, respectively. The blue-shaded area indicates the region where successful pick-up of the QD layer is achievable. For large QD pixels patterning (**b**), the F_{qq} does not significantly affect the pick-up of the QD layer, as the QD-QD interparticle cohesive force induced by van der Waals interactions primarily serves to maintain the integrity of the QD layer as a thin film. In contrast, for small QD pixel patterning (c), the influence of F_{qq} becomes relatively more pronounced, making the pick-up process more challenging. **d**,**e**, Cross-sectional schematic illustrations of the conventional TP process during pick-up step for large QD pixel patterning (d) and small QD pixel patterning (e).

Supplementary Fig. 6: QD pixel patterning in high resolution by conventional TP.

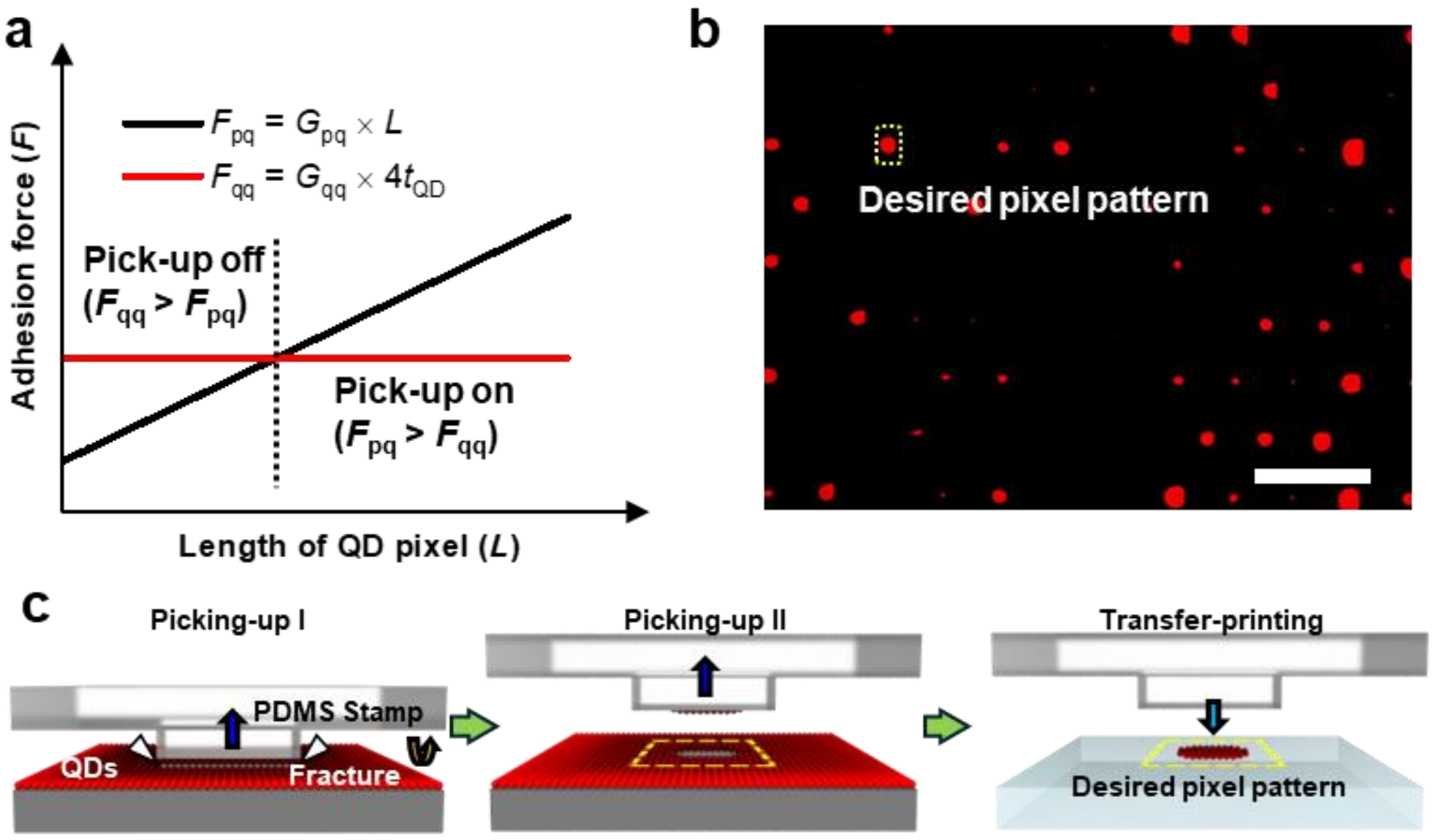

a, Schematic diagrams of adhesion force as a function of the length of the QD pixel for the micro-bump PDMS stamp, illustrating the ON and OFF states during the pick-up process. As the length of the QD pixel (L) decreases, F_{pq} (proportional to L) sharply decreases, while F_{qq} (proportional to t_{QD}) remains constant. This disparity makes the pick-up and patterning of small QD pixels infeasible. **b**, Experimental results of pixel patterning in the conventional TP process. Scale bar: 150 µm. **c**, Key steps in the conventional TP process, where the PDMS bump directly picks up QDs from the fully QD-coated donor substrate. The conventional TP process exhibits a very low pick-up yield, as the predominant interparticle cohesive forces (F_{qq}) hinder fracture at the edges of the QD pixels during the high-resolution QD pixelization process.

Supplementary Fig. 7: Dimension-dependent square pixel transfer yield in conventional and cracking-assisted transfer printing.

Pixel size (µm)	TP	CATP	Pixel Per Inch (PPI)
300	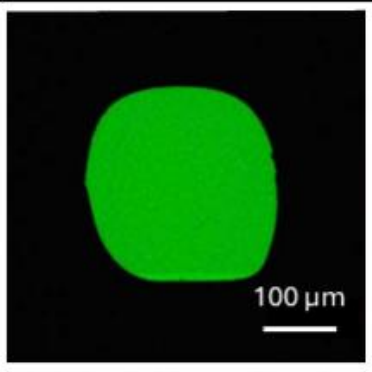	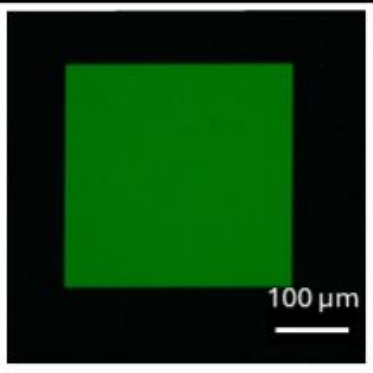	84
200	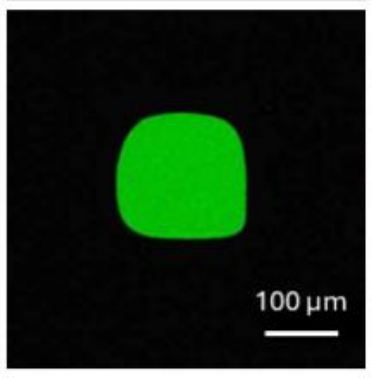	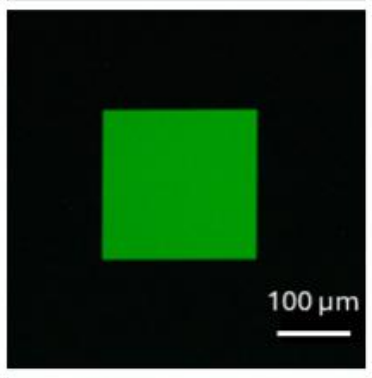	127
100	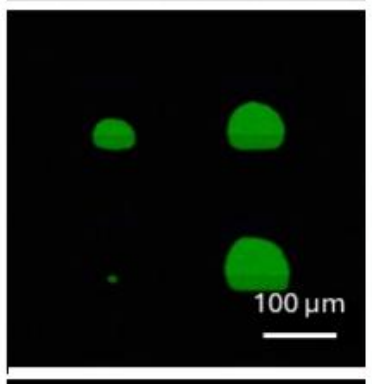	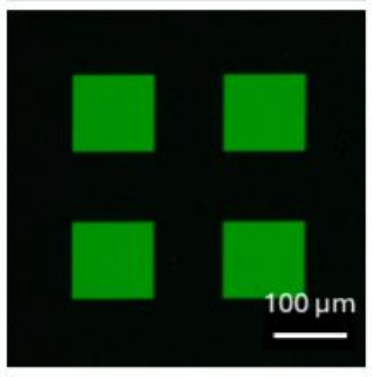	254
60	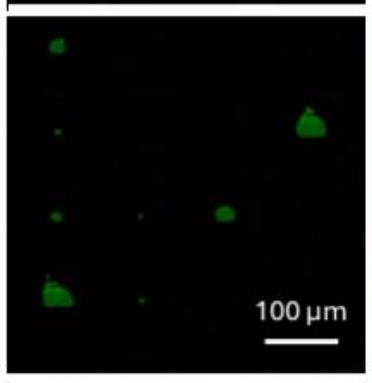	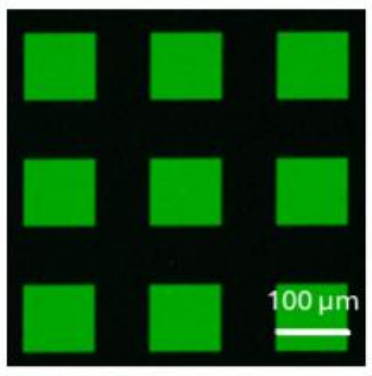	423
30	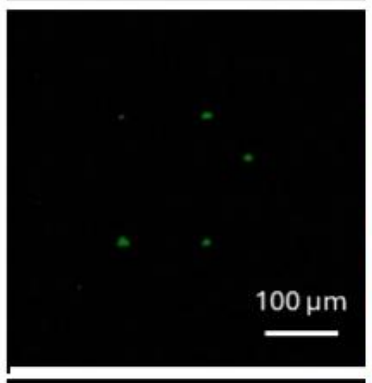	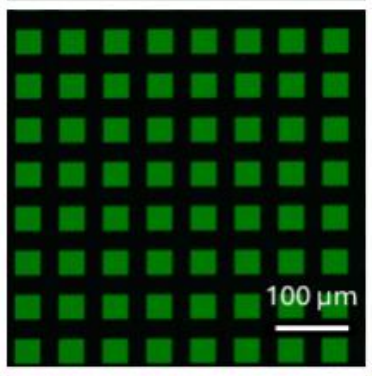	847
12	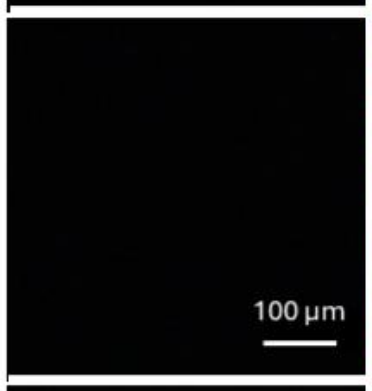	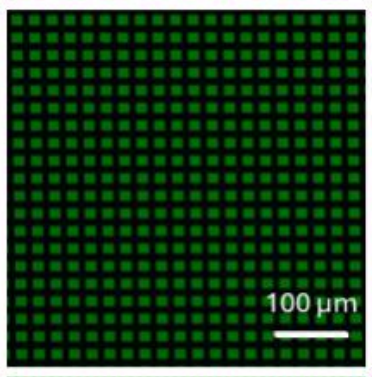	2117
6	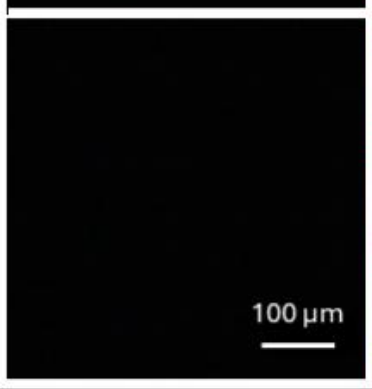	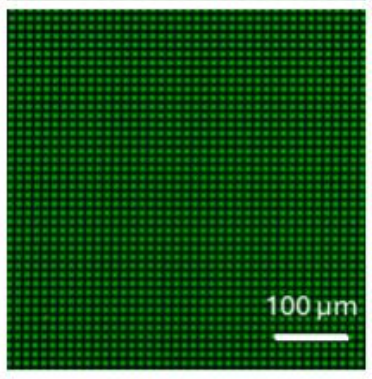	4233

Square pixel transfer yield versus pixel dimension for conventional transfer printing and CATP. As the designed square pattern size decreases, the transfer yield in the conventional TP method dramatically decreases. By contrast, the transfer yield in the CATP method is ~100% regardless of pattern size.

Supplementary Fig. 8: Schematic illustration of potential contamination and damage during the multi-patterning process using flat and micro-structured PDMS stamps.

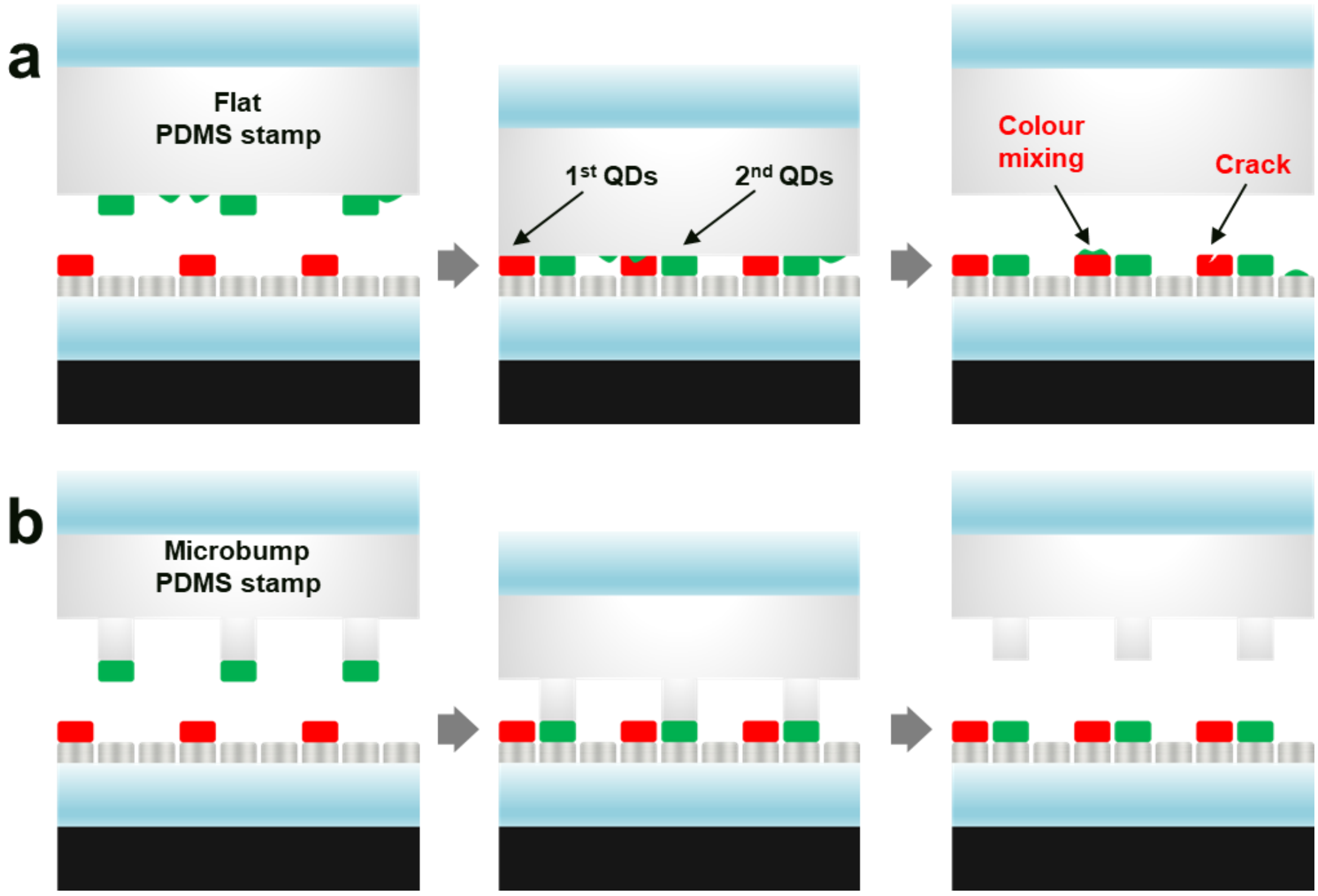

a, In the transfer printing process using a flat PDMS stamp, the PDMS must come into contact with the patterned QDs and the underlying CTL, which can leave residual QDs that are not completely removed in the intaglio process or cause damage to the patterned QDs. **b,** In the transfer printing processes using micro-structured PDMS stamps, the problem of cross-contamination and damage to patterned QDs can be avoided by eliminating unnecessary contact of the PDMS.

Supplementary Fig. 9: Analytical and simulated tensile stresses at the contact edge of the QD layer during the pick-up process using a three-dimensional solid mechanics model for conventional TP and CATP.

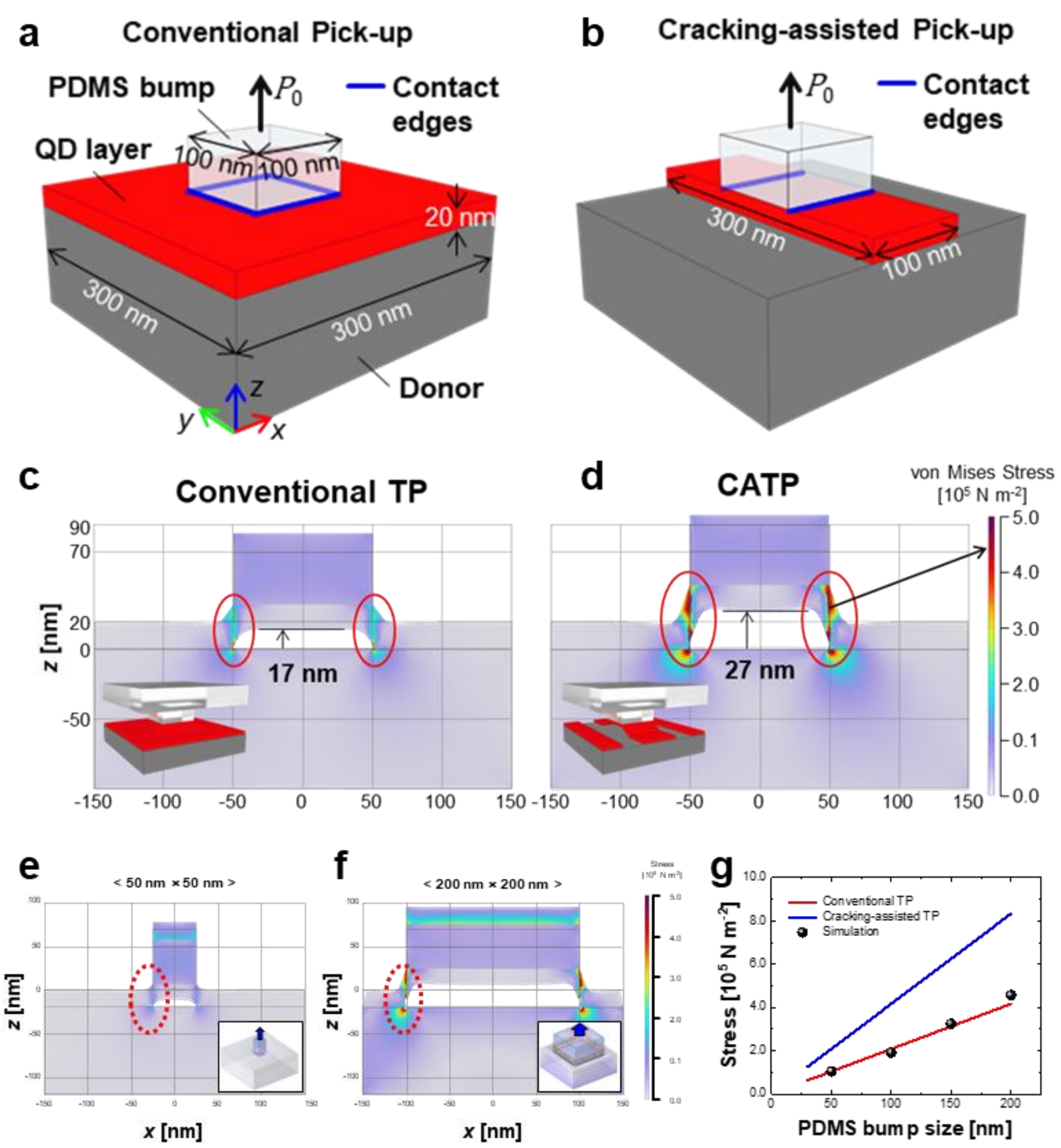

a, Simulation templates of solid mechanics analysis for conventional TP with fully coated 300 nm × 300 nm QD layer. **b**, Simulation templates of solid mechanics analysis for CATP with stripe-patterned 100 nm × 300 nm QD layer. **c**,**d**, Simulated von Mises stress in the QD layer for the conventional TP (**c**) and CATP (**d**). In the conventional TP, approximately 2.0×10^5 N m^{-2} von Mises stress at the edge of the QD layer is generated with a 17 nm gap between the QD pixel layer and substrate. In the CATP process, von Mises stress of around 4.5×10^5 N m^{-2} is generated with a larger gap of 27 nm, facilitating the effective creation of the fracture at the edge of the desired QD pattern. **e**,**f**, Distribution of tensile stress within the QD layer in the pick-up step of the conventional TP for 50 nm × 50 nm (**e**) and 200 nm × 200 nm (**f**) PDMS bump sizes. The 50 nm × 50 nm PDMS bump shows lower tensile stress within the QD layer compared with the 200 nm × 200 nm PDMS bump in the conventional TP process. **g**, Analytical and simulated tensile stresses at the edge of the QD layer for various PDMS bump sizes in the conventional TP and CATP. As the PDMS bump size increases, the tensile stress at the QD layer becomes larger in both the conventional TP and CATP processes. CATP exhibits higher tensile stress due to the partial elimination of QDs via the cracking, facilitating the pick-up of the high-resolution QD pixels.

Supplementary Fig. 10: OM images of nanoparticle and quantum dot with different material and surface ligands patterned via CATP.

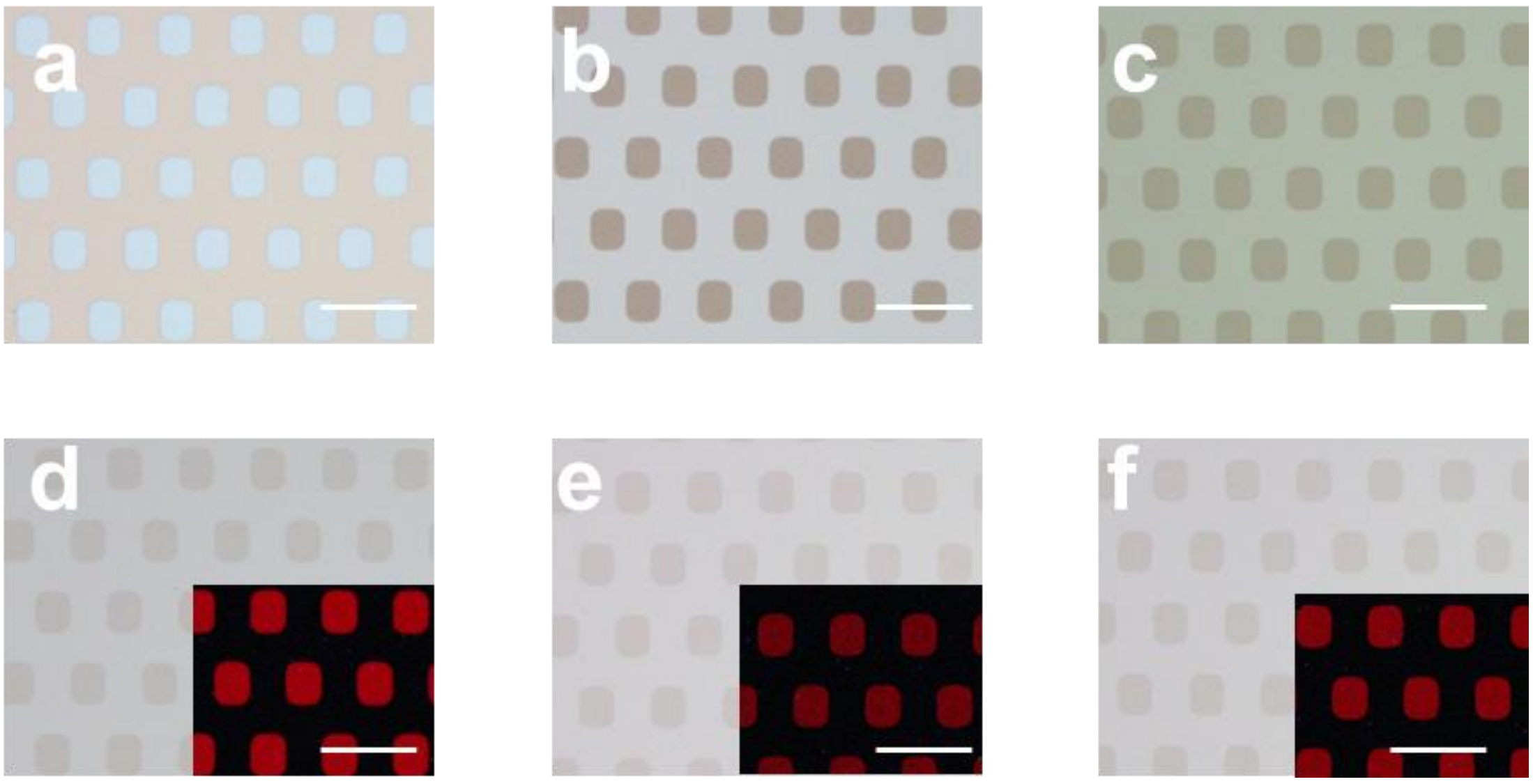

a, Ag nanoparticles. **b,** NiO nanoparticles. **c,** ZnO nanoparticles. **d,** Cd-based red QDs. **e,** Cd-based red QDs treated with EDT ligands. **f,** Cd-based red QDs treated with TBAI ligands. Insets show corresponding PL images. Scale bar 100 μm.

Supplementary Fig. 11: Transfer-printed cross-pattern QD films with controlled thickness.

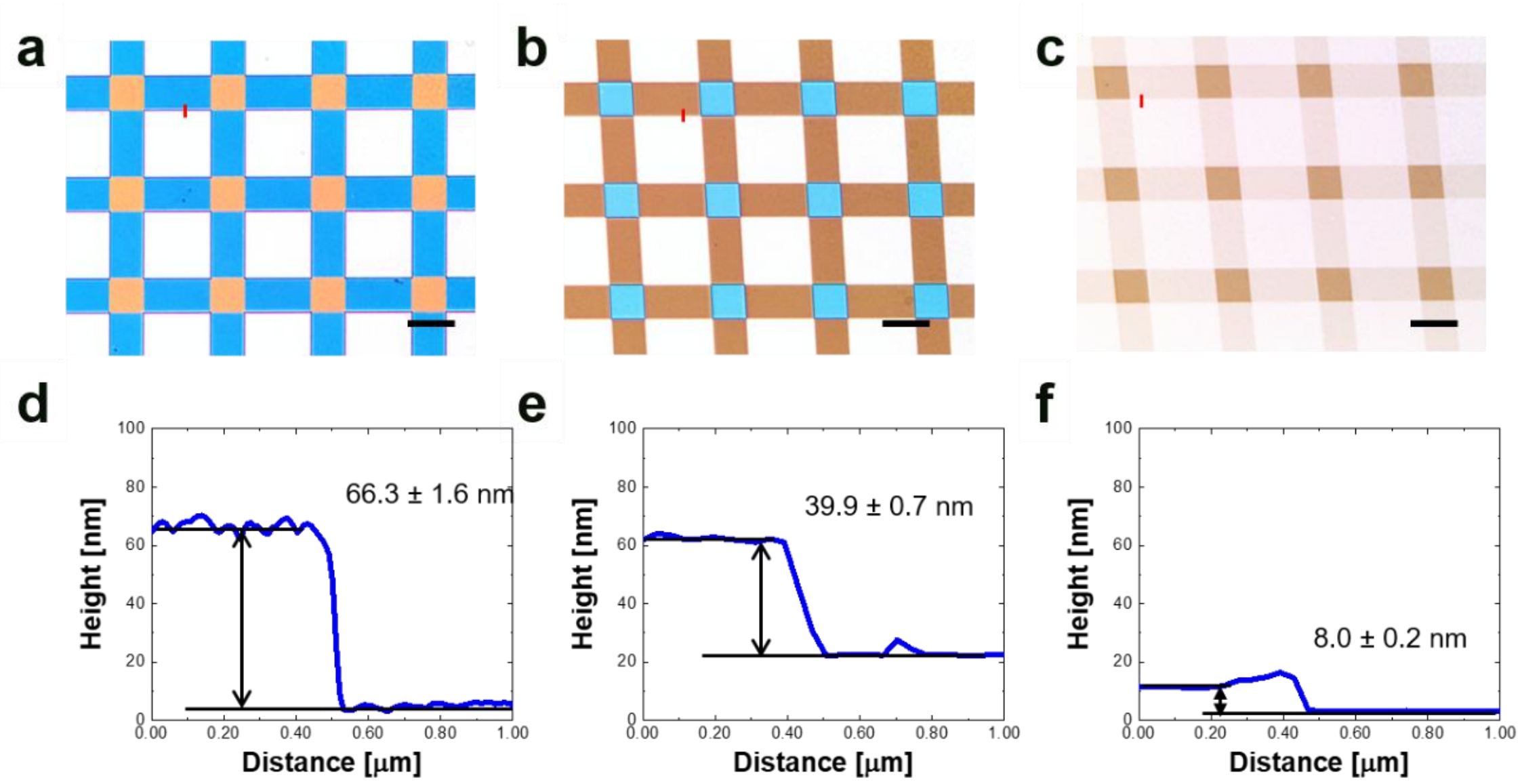

a–c, Optical microscopy images (scale bar: 100 µm) of transfer-printed QD cross-patterns fabricated from donor substrates with QD concentrations of 75mg ml^{-1} (a), 45 mg ml^{-1} (b), and 12 mg ml^{-1} (c), at a spin speed of 3000 rpm. **d–f,** corresponding AFM height profiles of the ZnSeTe/ZnSe/ZnS QD cross-patterns formed by a single transfer-printing step, measured along the positions indicated by the red lines in (a–c).

Supplementary Fig. 12: RGB QD stack fabricated via CATP.

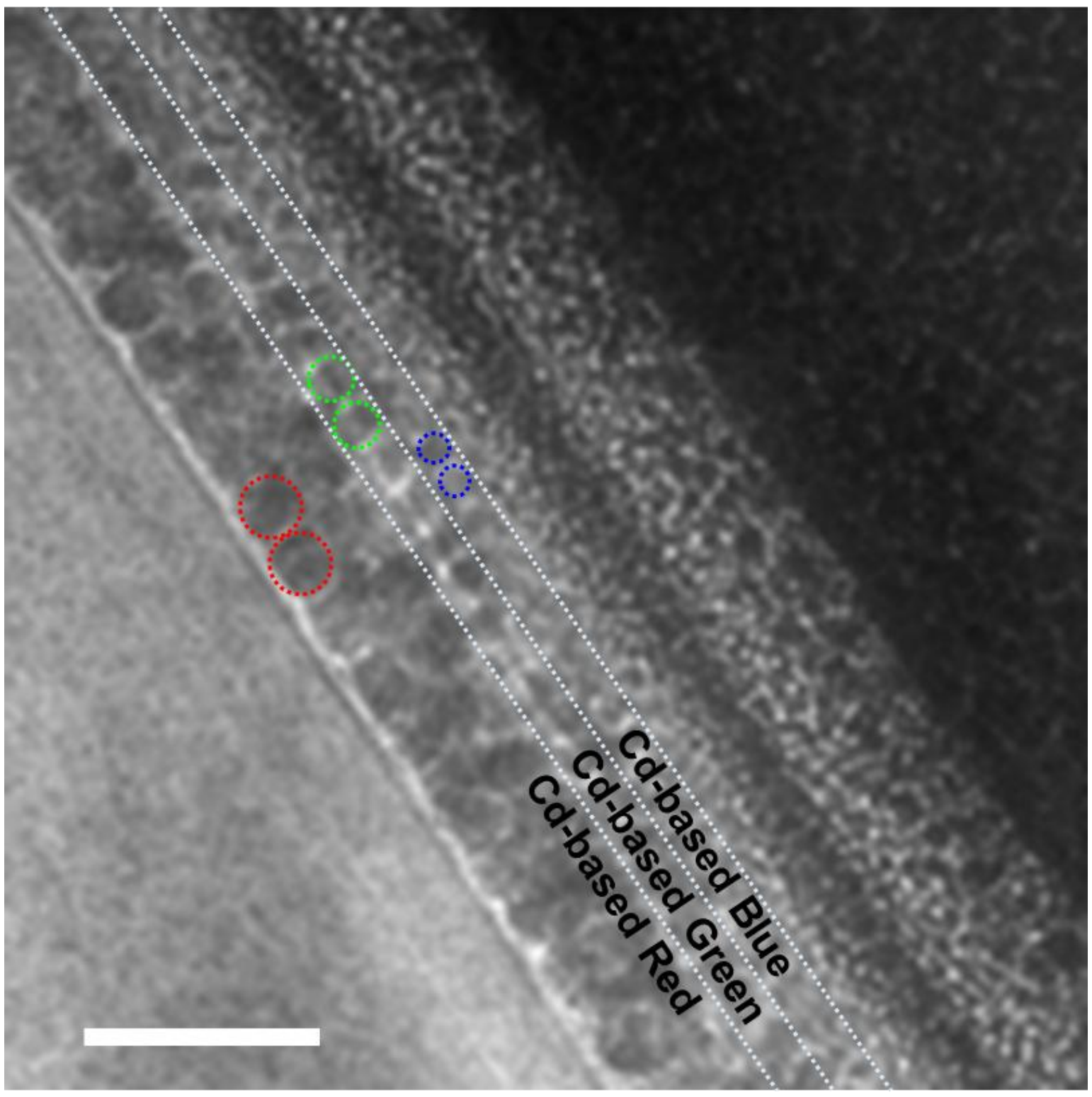

Cross-sectional TEM image of the multistacked QD layer in the sequence of R/G/B. Scale bar, 50 nm.

Supplementary Fig. 13: Multilayer QD pickup and transfer mechanism in CATP.

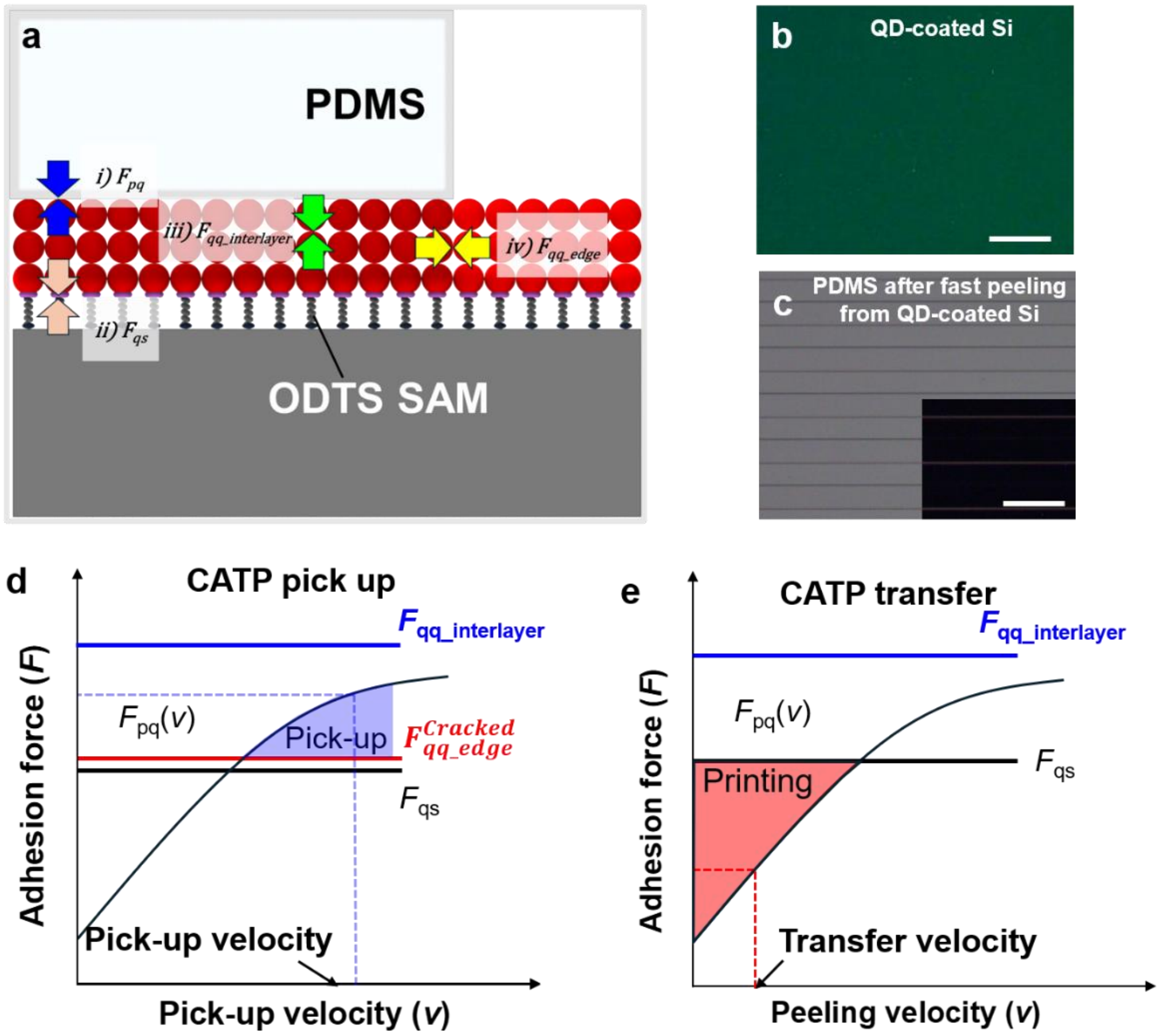

a, Schematic illustration of the competing interactions involved in CATP for a multilayer QD film on an ODTS-treated substrate, including (i) the PDMS–QD adhesion force (F_{pq}), (ii) the QD–substrate adhesion force (F_{qs}), (iii) the interlayer QD–QD cohesive force ($F_{qq\_interlayer}$), and (iv) the lateral edge QD–QD cohesive force (F_{qq_edge}). During pickup, a fracture is intentionally induced at the lateral edge of the QD–QD connections while preserving interlayer integrity. **b-c,** OM showing the no QD pickup from an ozone-treated Si substrate (**b**), to the PDMS stamp (**c**). Scale bar: 100 μm. **d,** Qualitative schematic of the force hierarchy during the pickup step, where $F_{qq\_edge}^{Cracked} < F_{pq}$ (fast pick-up) $< F_{qq\_interlayer}$, enabling edge-initiated cracking without interlayer delamination. **e,** Qualitative schematic of the transfer step, where reduced PDMS–QD adhesion at low peeling velocity, F_{pq} (slow transfer) allows release of the intact multilayer QD film onto the target substrate.

Supplementary Fig. 14: Uniformity of CATP-printed QD pixels with tens-of-micrometre dimensions.

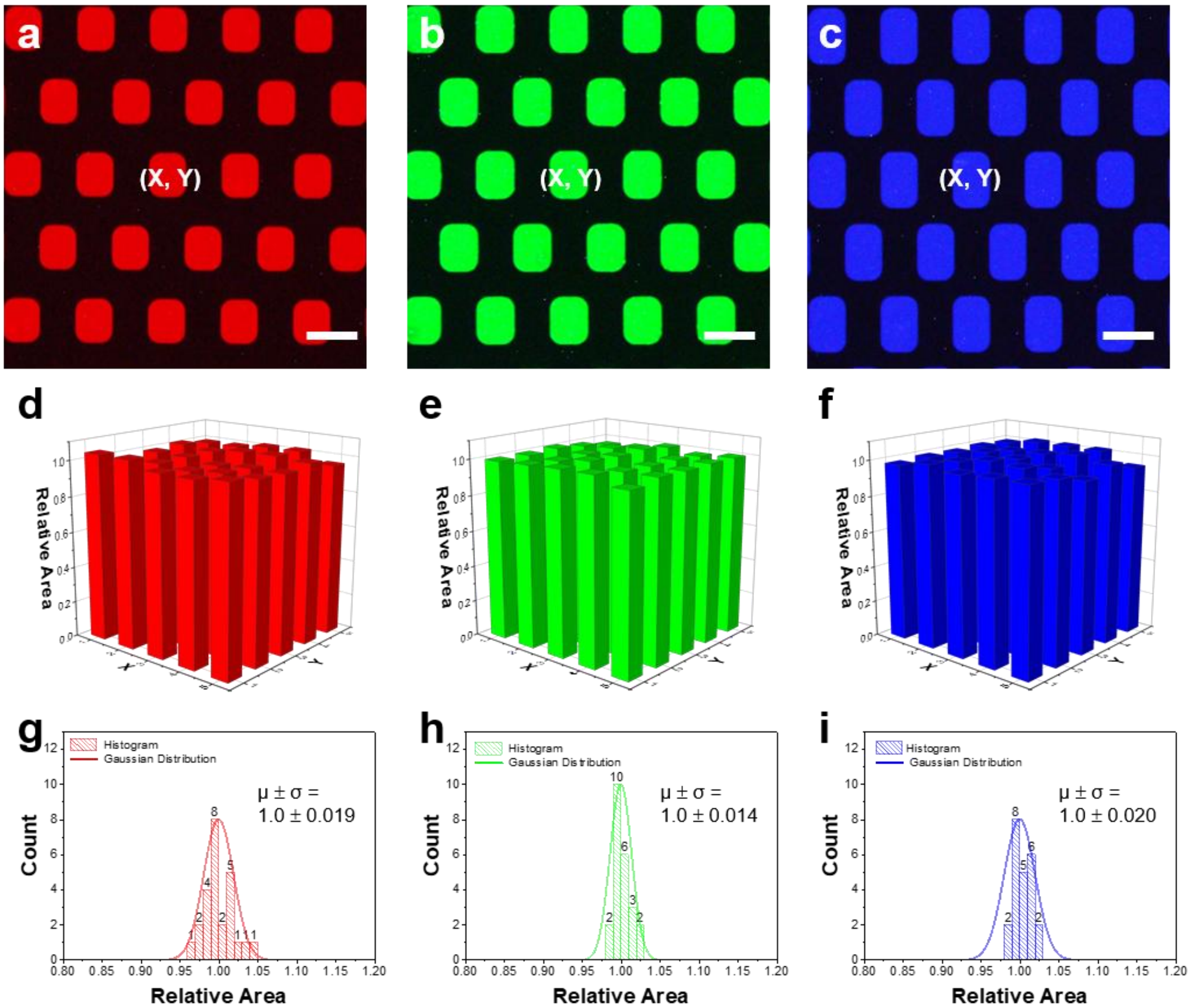

Top-view optical EL images of **a**, red (37 × 46 μm²), **b**, green (37 × 47 μm²), and **c**, blue (37 × 56 μm²) QD pixel arrays. Scale bar, 50 μm. 3D bar plots of relative pixel areas for **d**, red, **e**, green, and **f**, blue, extracted from 25 samples (N = 5). Gaussian-fitted histograms for **g**, red, **h**, green, and **i**, blue QD patterns, showing narrow distributions with σ = 0.019 (red), 0.014 (green), and 0.020 (blue), indicating high uniformity at display-scale pixel dimensions.

Supplementary Fig. 15: Uniformity of CATP-printed 2 × 2 μm² QD pixels.

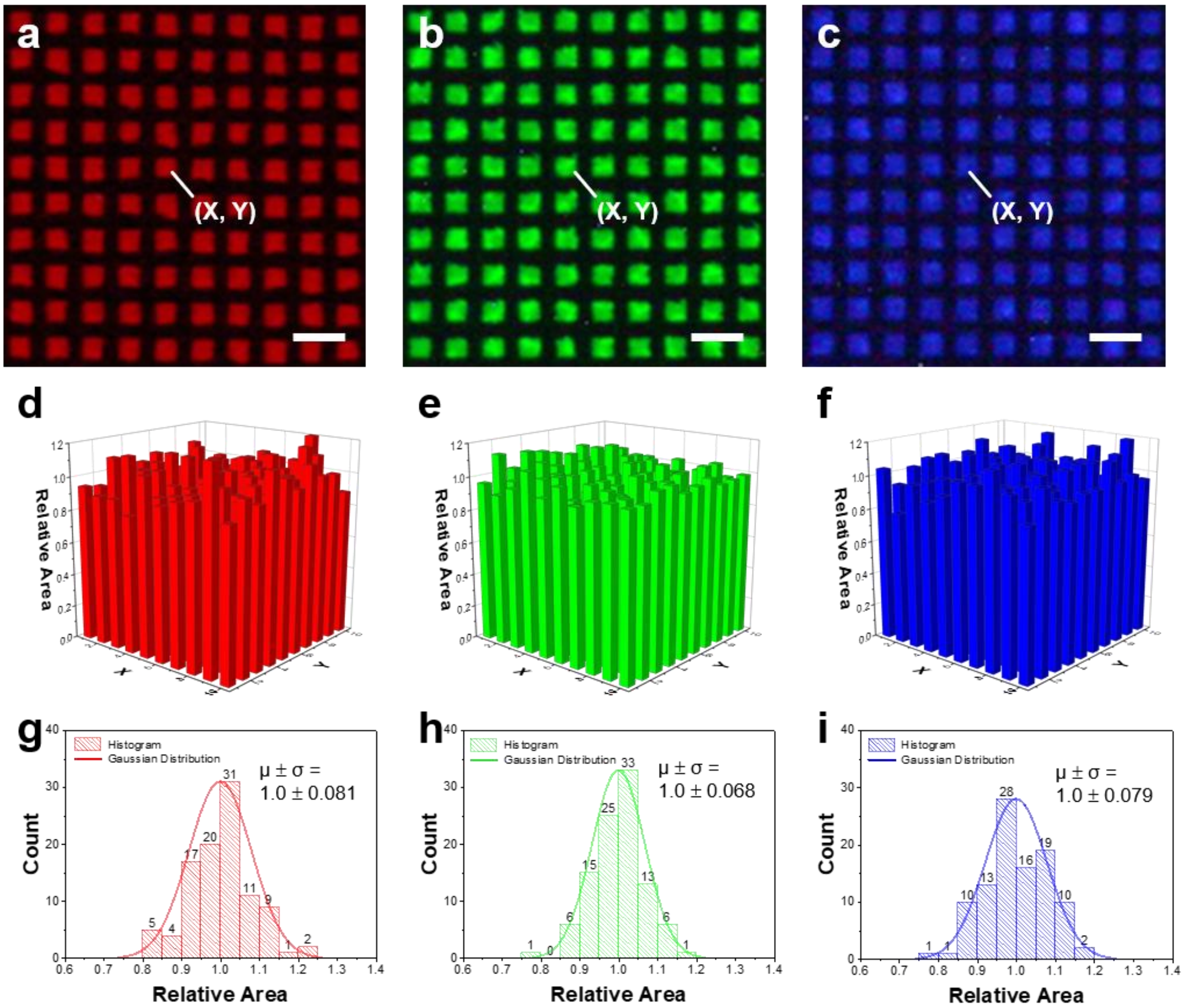

Top-view optical EL images of **a**, red, **b**, green, and **c**, blue 2 μm pixels. Scale bar, 5μm. 3D bar plots of relative pixel areas extracted from 100 samples (N = 10) for **d**, red, **e**, green, and **f**, blue QD patterns. Gaussian-fitted histograms showing σ = 0.081, 0.068, and 0.079 for **g**, red, **h**, green, and **i**, blue QD patterns, reflecting increased variability due to reduced pixel size.

Supplementary Fig. 16: Uniformity of CATP-printed sub-micrometre (0.6 × 0.9 µm²) QD pixels.

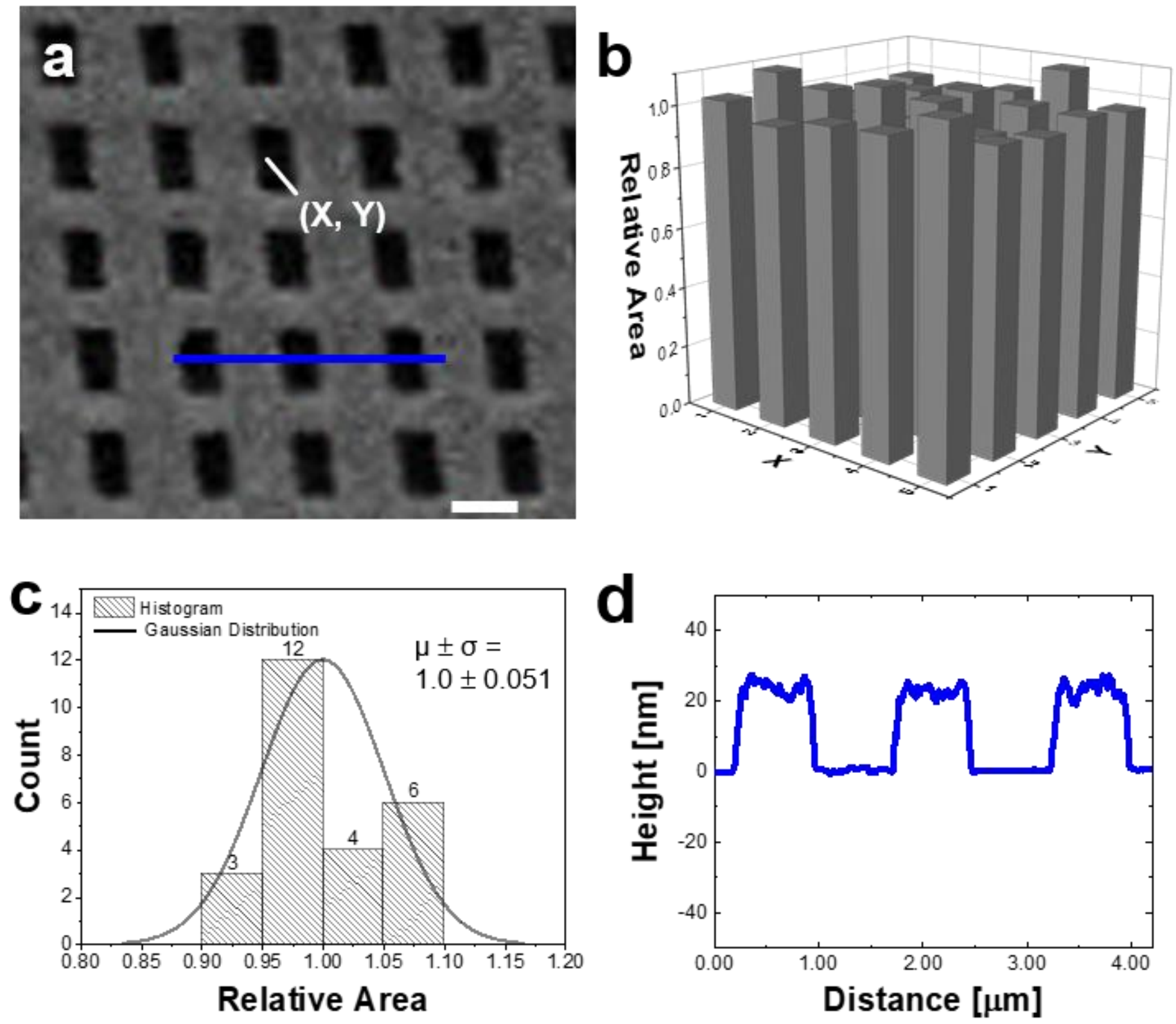

a, SEM image of sub-micrometre red QD pixel arrays. Bottom right white scale bar, 1 µm. **b**, 3D bar plot of relative pixel areas (N = 5). **c**, Gaussian-fitted histogram showing σ = 0.051, confirming reproducible pattern formation even at the nanometre scale. d, Corresponding AFM height profile along the blue line in (a), confirming distinct edge definition and uniform feature height across the patterned pixels.

Supplementary Fig. 17: CATP-enabled patterning of arbitrary pixel geometries.

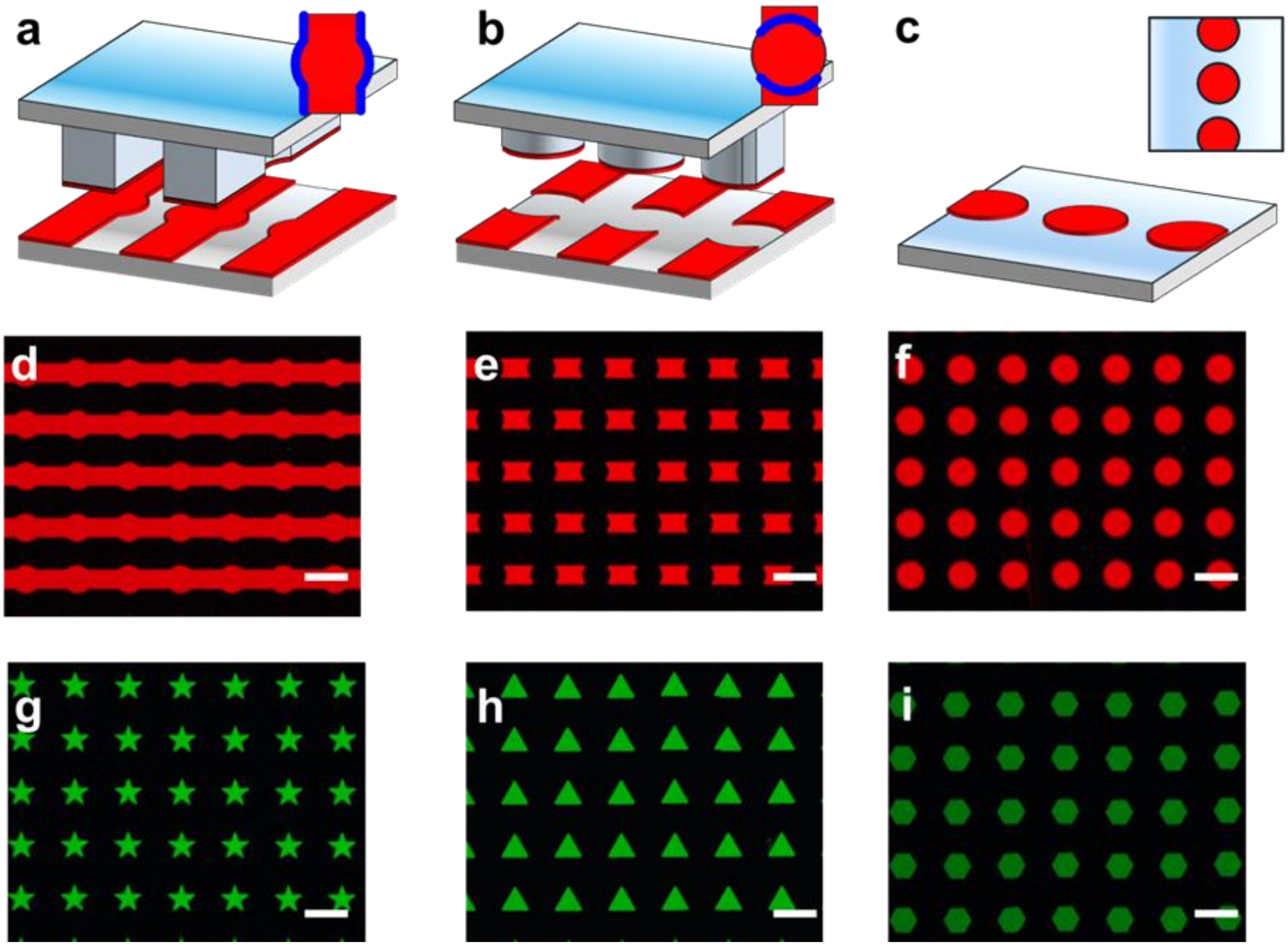

Schematic illustration of the CATP process for **a**, cracking step, **b**, pick-up step, and **c**, transfer printing step. Experimental verification of the schematic sequence for **d**, cracking, **e**, pick-up, and **f**, transfer printing steps. Demonstration of the geometric versatility of CATP with patterned QD pixels in **g**, star-shaped, **h**, triangular and **i**, hexagonal patterns. Scale bar, 50 µm.

Supplementary Fig. 18: Details of LTPS TFT backplane for AM EL QD displays.

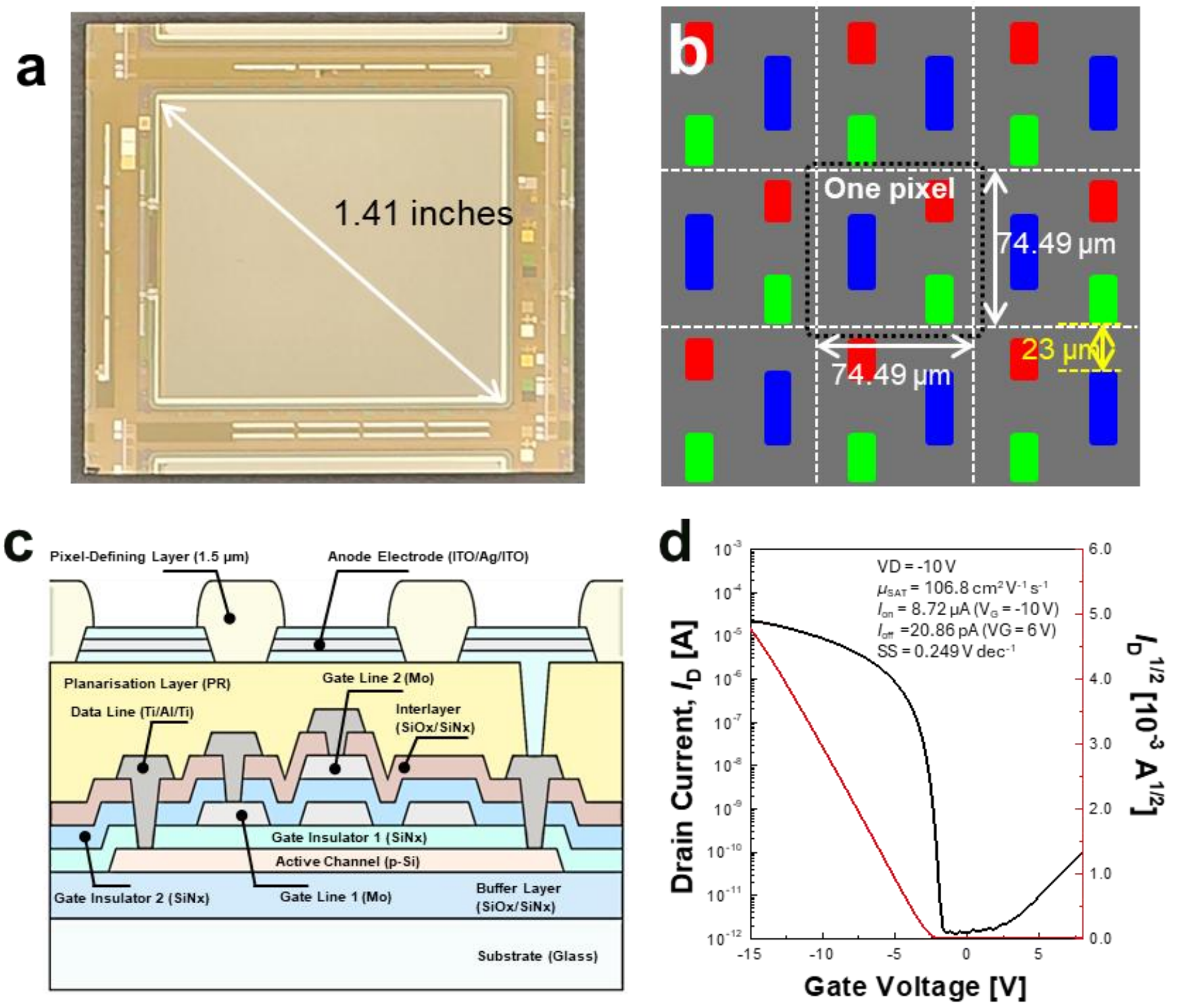

a, TFT backplane with an active area of 1.41 inches in diagonal size. **b**, Schematic illustration of RGB layout for pentile pixel structure with 74.49 μm × 74.49 μm size. **c**, Schematic illustration of cross-section architecture of the LTPS TFT backplane. **d**, Transfer characteristics of the driving LTPS TFTs (W/L = 3.5 μm/40 μ). TFT mobility, on/off ratio, and the sub-threshold swing (SS) are 106.8 cm^2 V^{-1} s^{-1}, 4.18×10^5, and 0.249 V dec^{-1}, respectively.

Supplementary Fig. 19: Preliminary test experiments by intaglio TP as a reference study for QD pixel patterning capability.

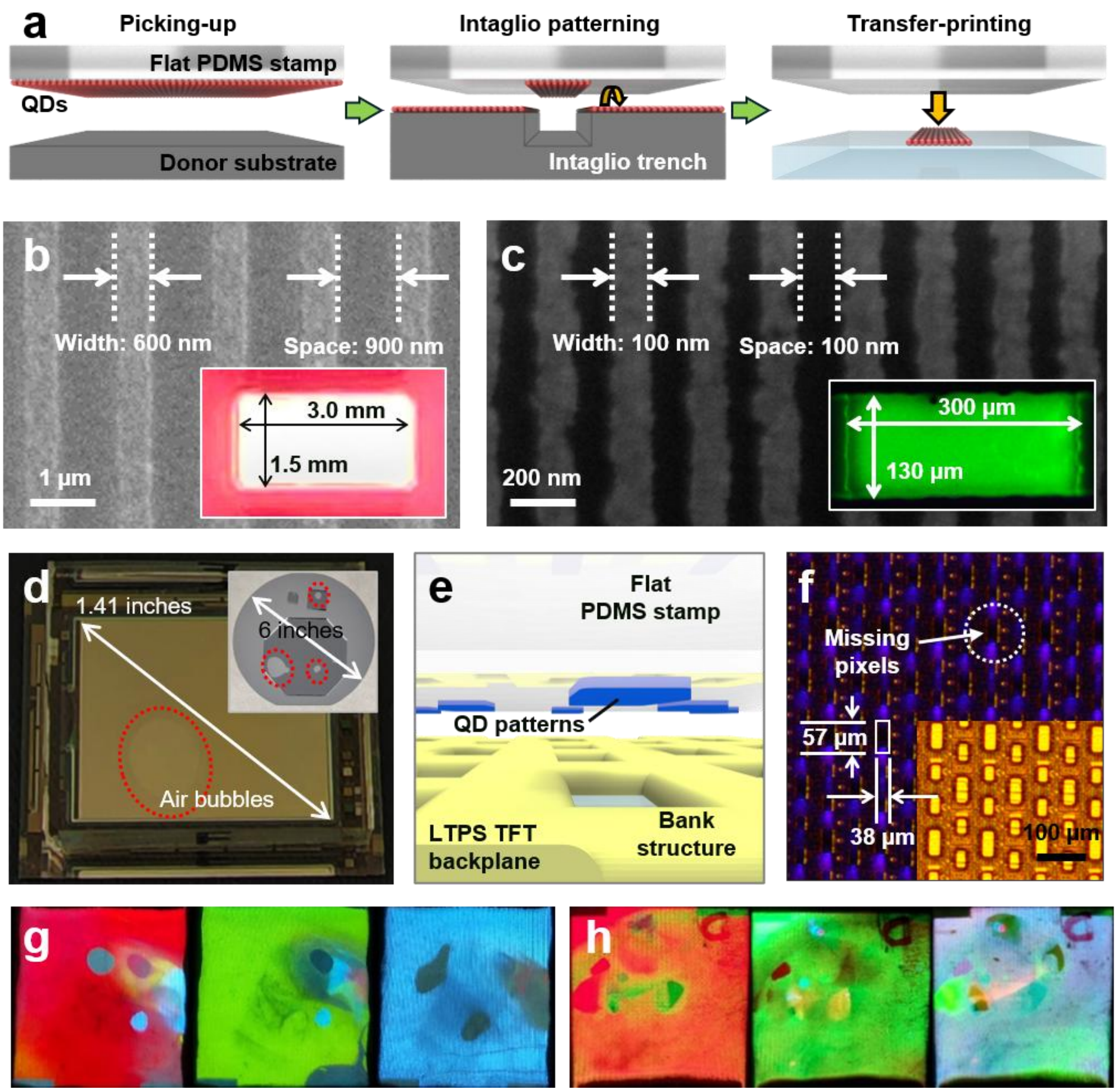

a, Key process steps of the intaglio TP with the flat PDMS stamp. The QD layer on a flat PDMS stamp is patterned by a rigid intaglio trench, and the patterned QDs are transfer printed on target substrates by the flat stamp. **b**, 600 nm line (900 nm space) stripe red QD patterns processed by intaglio TP (Inset: EL device with a luminance of 3,826 cd m^{-2} at 8 V). **c**, 100 nm line (100 nm space) stripe green QD patterns processed by intaglio TP (Inset: EL device with a luminance of 3,123 cd m^{-2} at 8 V). **d**, Air-bubble entrapment (dotted red line) between flat PDMS stamp and 1.41-inch TFT backplane (Inset: various sized flat stamps (0.6 inches, 1.2 inches, and 4 inches in diagonal) on planar wafer substrate with air-bubble issues). **e**, Topological mismatch (non-conformality) between flat stamp and non-planar TFT backplane due to bank structure. **f**, Unsuccessful pixelization of QD patterns by intaglio TP within the bank structure on the TFT backplane. **g**,**h**, Cd-free (**g**) and Cd-based (**h**) full-colour RGB EL QD display images on 1.41-inch TFT backplane by intaglio TP, showing the colour Mura caused by air-bubble entrapment and non-planar surface effect by TFT backplane.

Supplementary Fig. 20: Comparative analysis of air trapping for flat and microbumped PDMS stamps.

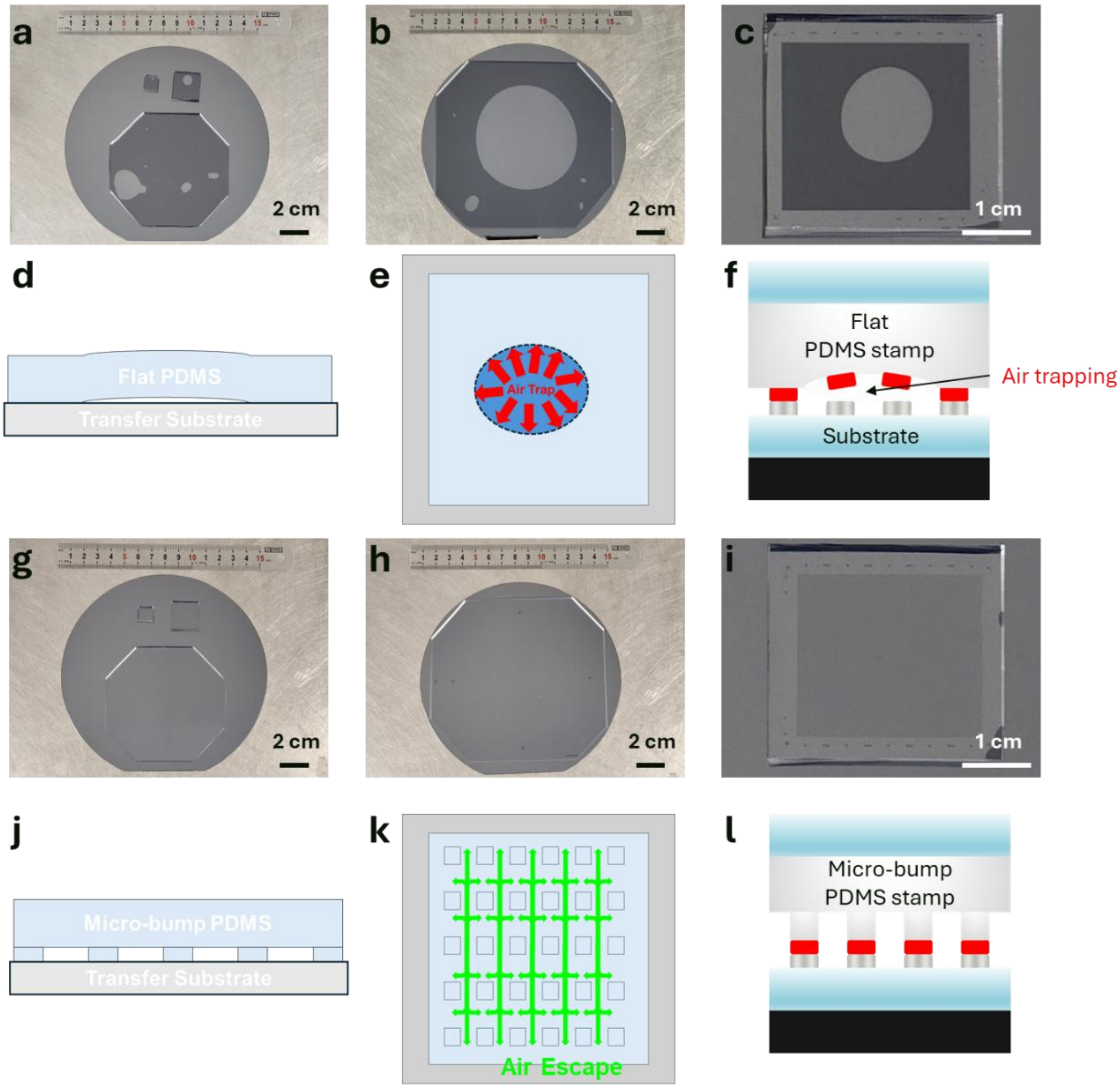

a-b, Photographs of flat PDMS stamps with varying diameters, ranging from 0.6 to 6 inches, placed on a 6-inch Si wafer. **c,** Photograph of a flat PDMS stamp used to fabricate a 1.41-inch active-matrix quantum dot display, placed on a 6-inch Si wafer. **c,** Photographs of flat PDMS stamp used to fabricate a 1.41-inch active matrix quantum dot display, placed on a 6-inch Si wafer. **d–e,** Schematic illustrations showing air trapping during the transfer process using a flat PDMS stamp, including side-view (**d**) and top-view (**e**) representations. **f,** Schematic illustration of air trapping during the transfer of QDs onto LTPS backplanes using flat PDMS stamps. **g-h,** Photographs of micro-bump PDMS stamps with varying diameters, ranging from 0.6 to 6 inches, placed on a 6-inch Si wafer. **i,** Photographs of micro-bump PDMS stamp used to fabricate a 1.41-inch active matrix quantum dot display, placed on a 6-inch Si wafer. **j–k,** Schematic illustrations showing suppressed air trapping and efficient air escape during the transfer process using a microstructured PDMS stamp, including side-view (**j**) and top-view (**k**) representations. **l,** Schematic illustration of conformal contact during the transfer of QDs onto LTPS backplanes using micro-bump PDMS stamps.

Supplementary Fig. 21: Effect of microstructure height and spacing on the collapse of striped PDMS stamps.

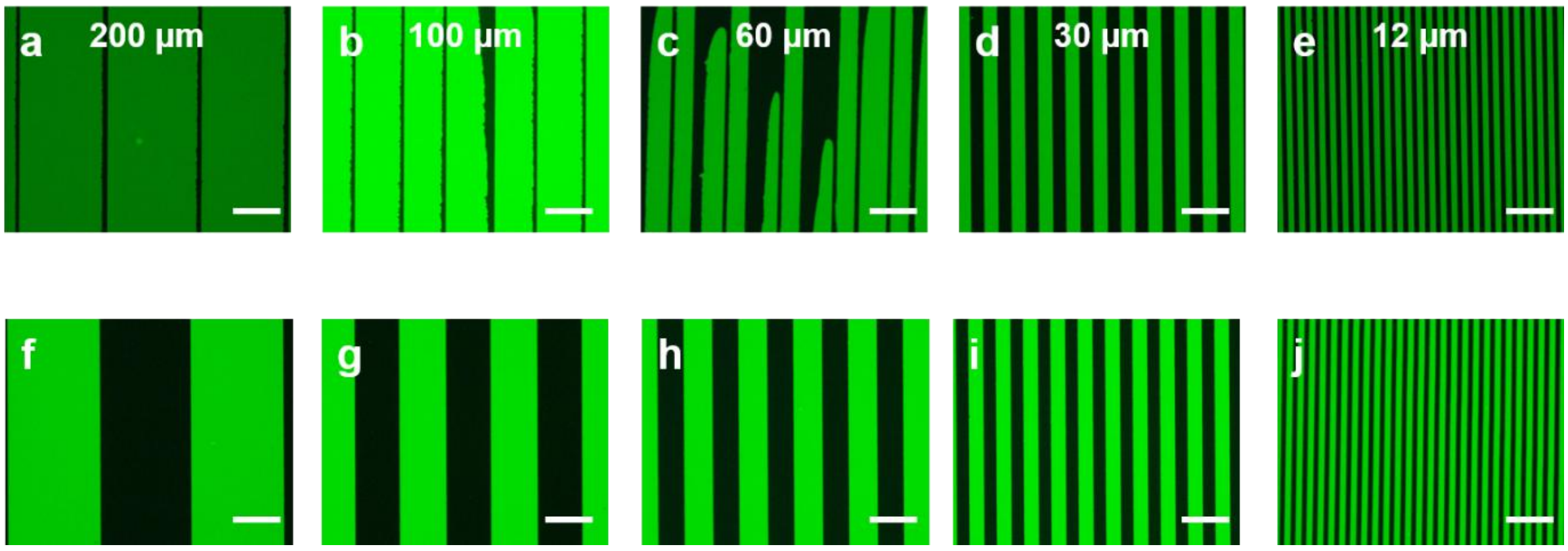

a–e, QD patterns transferred using PDMS stamps with a stripe height of 2 μm, with spacings of 200 μm (**a**), 100 μm (**b**), 60 μm (**c**), 30 μm (**d**), and 12 μm (**e**). Pattern distortion and non-uniform stripe widths observed at larger spacings (200–60 μm) indicate the onset of elastic sagging of the PDMS stamp during contact, whereas uniform patterns are maintained at smaller spacings (30 and 12 μm). **f–j,** QD patterns transferred using PDMS stamps with a stripe height of 10 μm, with spacings of 200 μm (**f**), 100 μm (**g**), 60 μm (**h**), 30 μm (**i**), and 12 μm (**j**), exhibiting uniform and well-defined stripe patterns across all tested spacings.

Supplementary Fig. 22: Uniformity comparison of 5 µm QD square patterns fabricated by CATP and intaglio transfer printing for 4 inch.

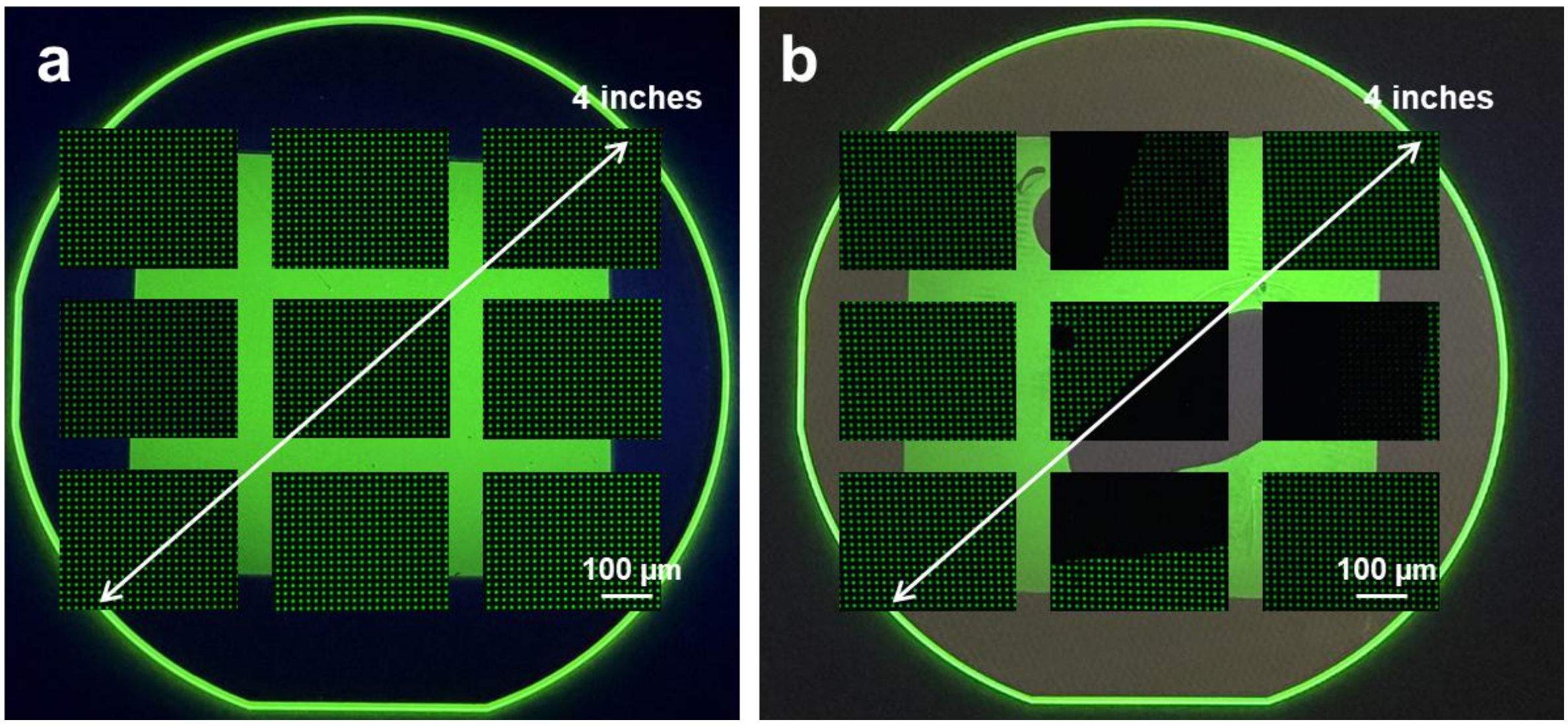

a-b, PL images of 5 µm QD dot arrays patterned over a 4-inch glass substrate using CATP (**a**) and intaglio transfer printing (**b**). CATP yields uniform, continuous PL emission across the entire substrate, whereas intaglio transfer printing exhibits multiple dark regions due to missing or incomplete dot transfer. Insets show representative regions of the 5 µm dot patterns.

Supplementary Fig. 23: PDMS stamp cleaning with PI tape.

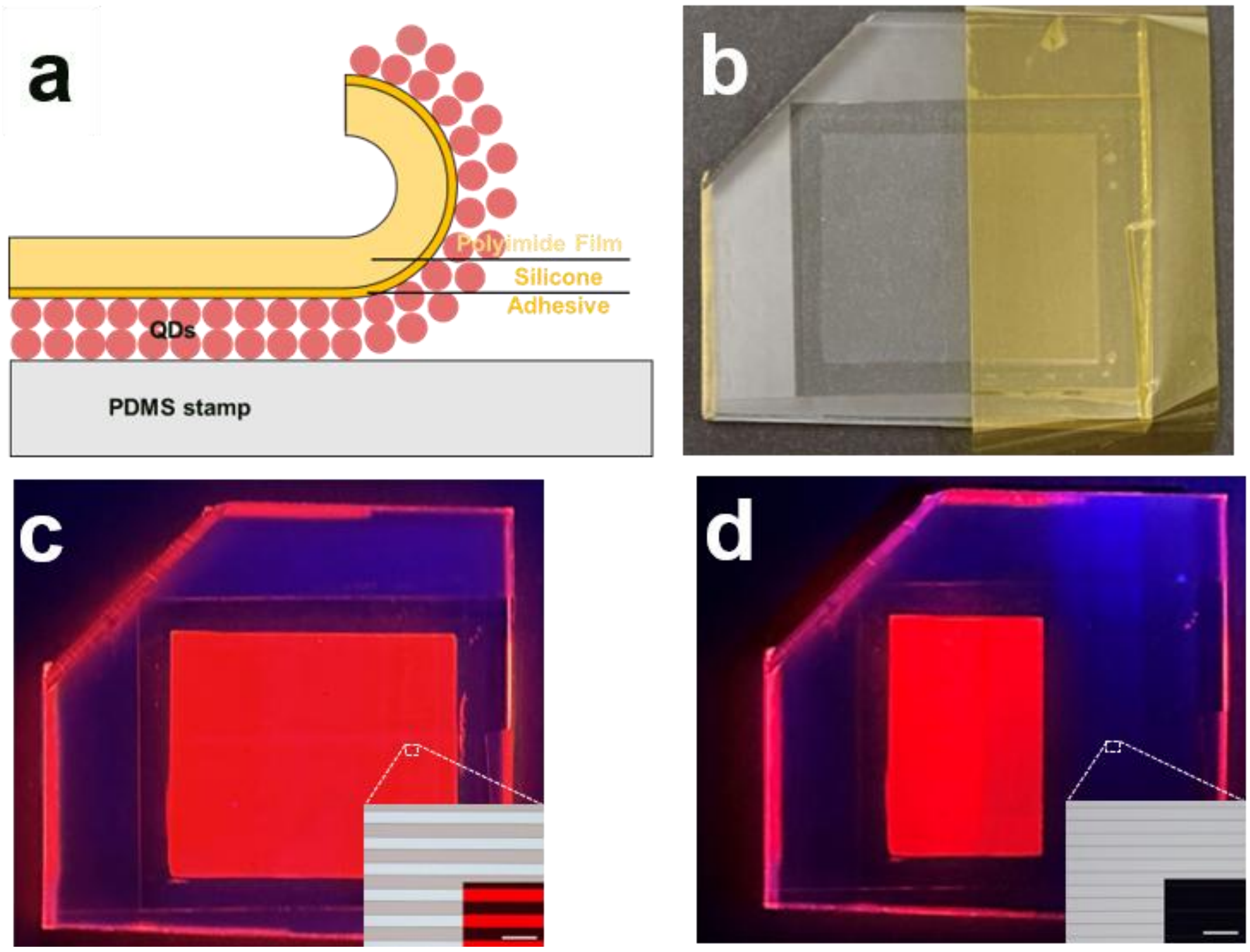

a, Schematic illustration of the cleaning process, where the silicone-based adhesive layer of PI tape peels off QDs from the PDMS surface. **b,** Image of PI tape used for cleaning of PDMS stamp **c,d,** PL images of the PDMS stamp before (**c**) and after (**d**) the cleaning process. Insets: corresponding fluorescence microscope images of the QD region (Scale bar, 100 μm).

Supplementary Fig. 24: PDMS stamp recycle test.

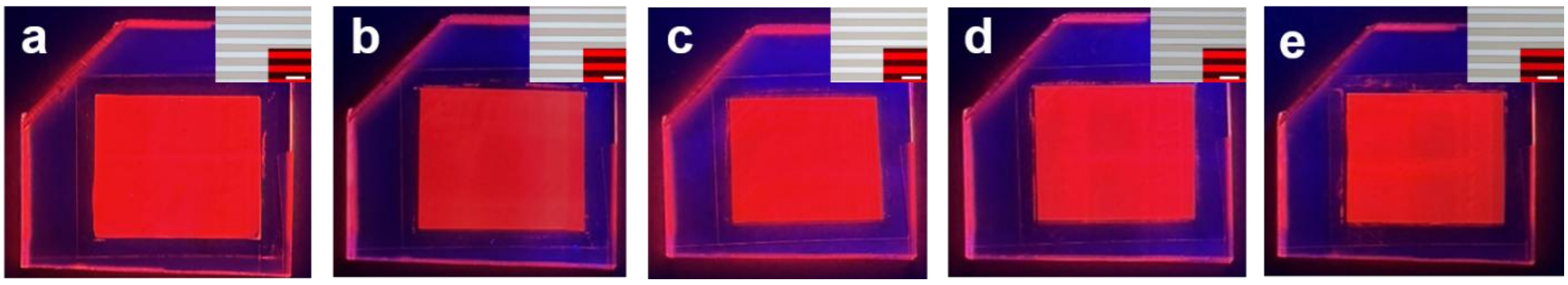

a-e, PL images of 35 μm CdSe/ZnS red QD line patterns after the 1st transfer (**a**), the 10th transfer (**b**), the 50th transfer (**c**), the 70th transfer (**d**), and the 100th transfer (**e**). Inset: Optical microscope image. All white scale bars, 100 μm.

Supplementary Fig. 25: Schematic illustration of potential QD wastage during intaglio TP and CATP.

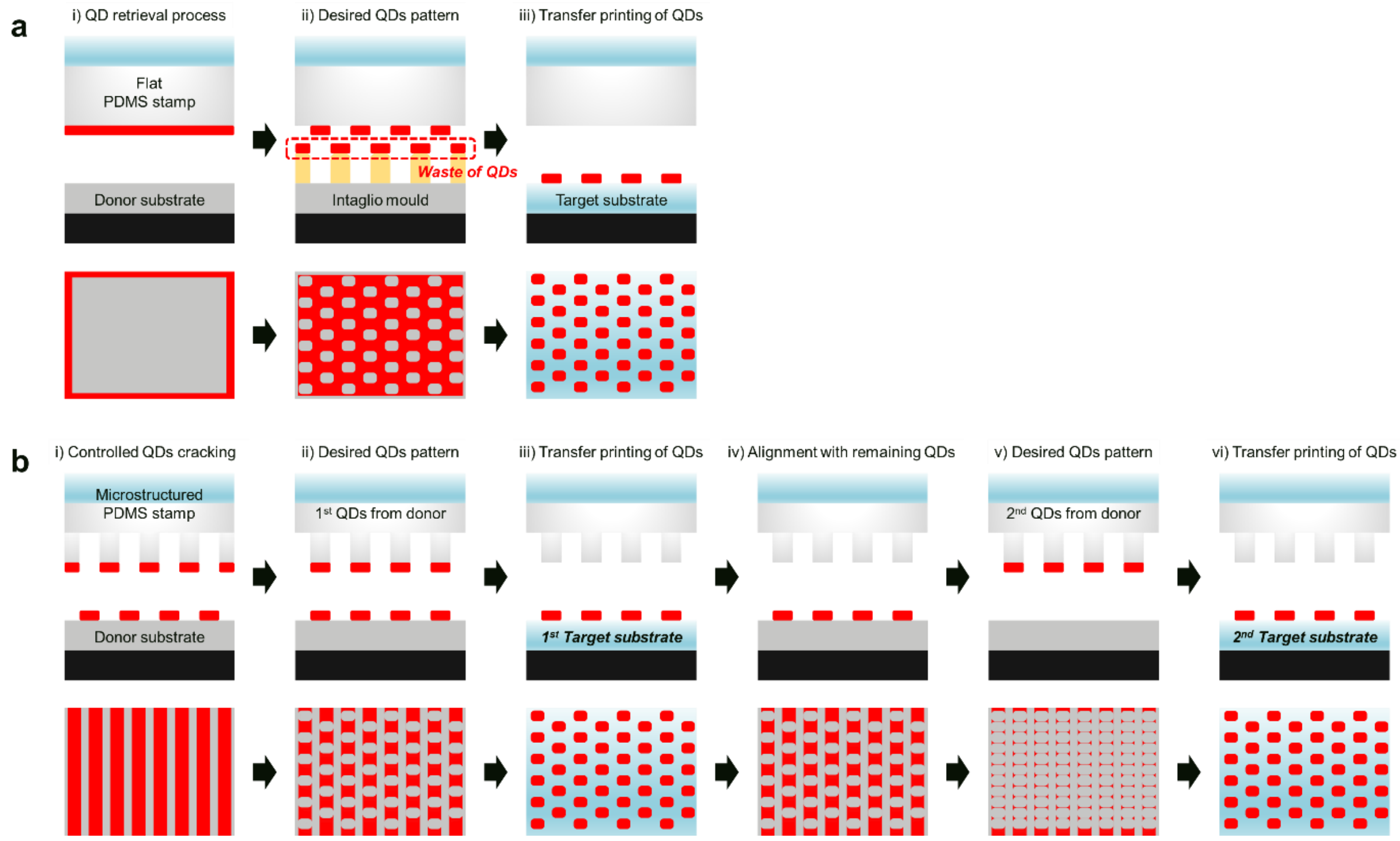

a, In the intaglio transfer printing process using flat PDMS stamps, QDs that contact the intaglio trench for patterning cannot be reused and are discarded. **b,** In the cracking-assisted transfer printing process using microstructured PDMS stamps, the QDs on the donor substrate can be reused through alignment, reducing the waste of QD material.

Supplementary Fig. 26: 8-inch-scale transfer enabled by a stitching method.

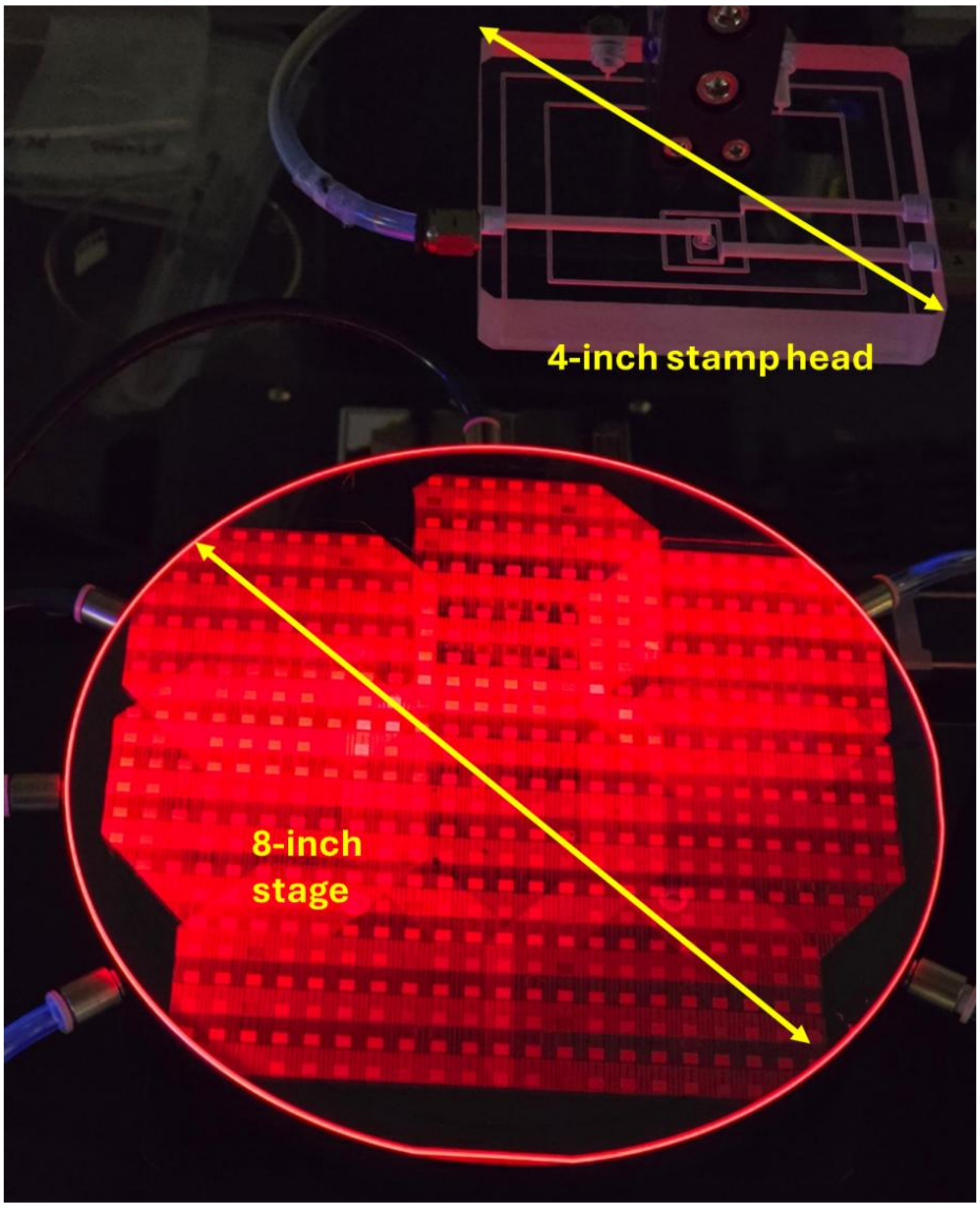

PL photograph of the stitching transfer process, where a 4-inch PDMS stamp is used to sequentially transfer across an 8-inch stage to achieve large-area pattern transfer.

Supplementary Fig. 27: Integration of RGB full-colour pixel arrays on TFT backplane.

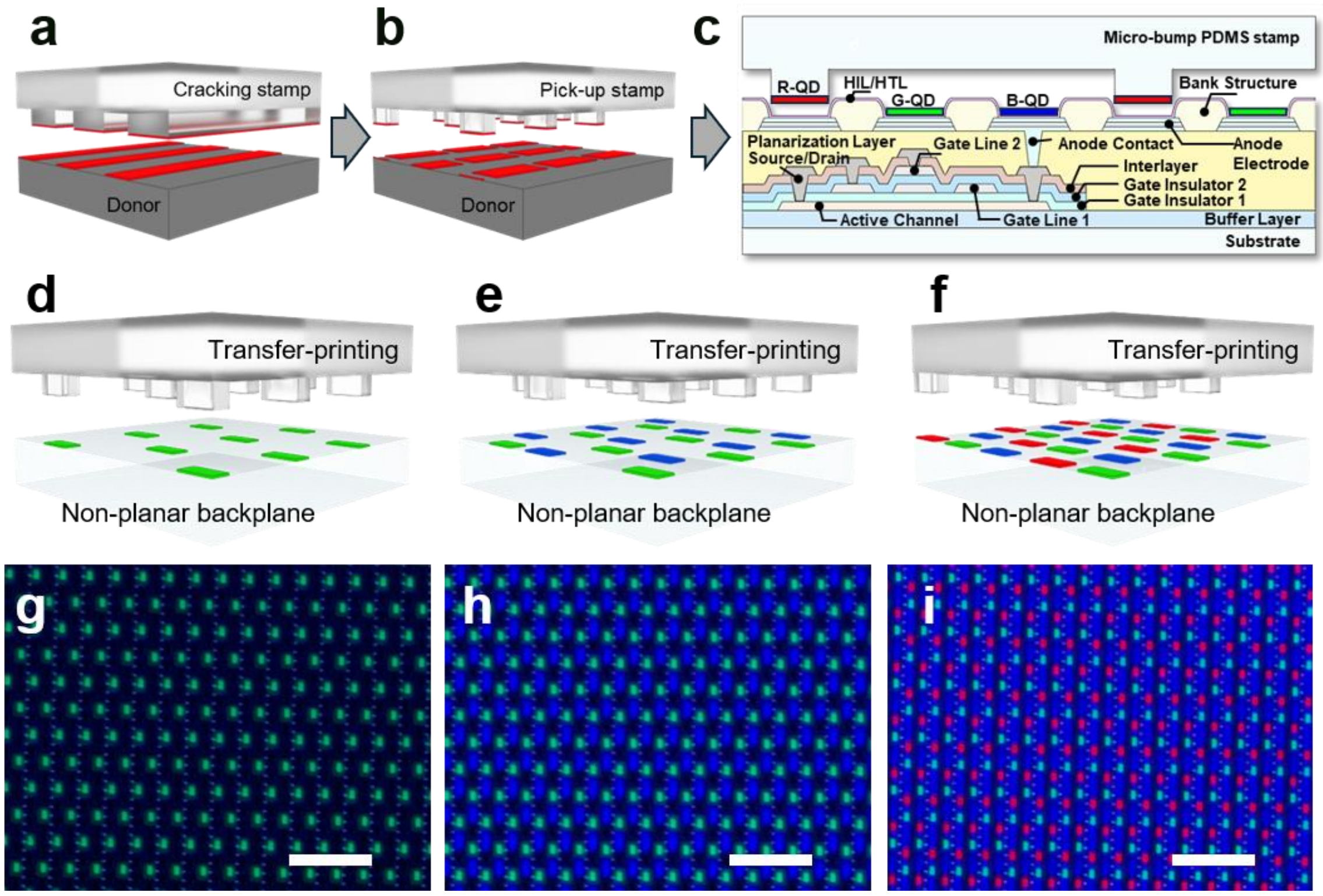

a,**b**, Red QD dot patterns on micro-bump PDMS stamp by the cracking (**a**) and pick-up (**b**) steps on donor substrate. **c**, Cross-section of the non-planar LTPS TFT backplane having bank structure with micro-bump PDMS stamp for red, green, and blue QD pixelization within the designated pixel area. **d-f**, Sequential transfer printing of green (**d**), blue (**e**), and red (**f**) pixels to the designated location on the target TFT backplane with PL images of green (**g**), green-blue (**h**), and green-blue-red (**i**) pixelated QD layers during sequential integration for full-colour pixel array. Scale bars, 200 µm.

Supplementary Fig. 28: Design of red, green, and blue QD patterns for the pentile subpixel layout on the LTPS TFT backplane.

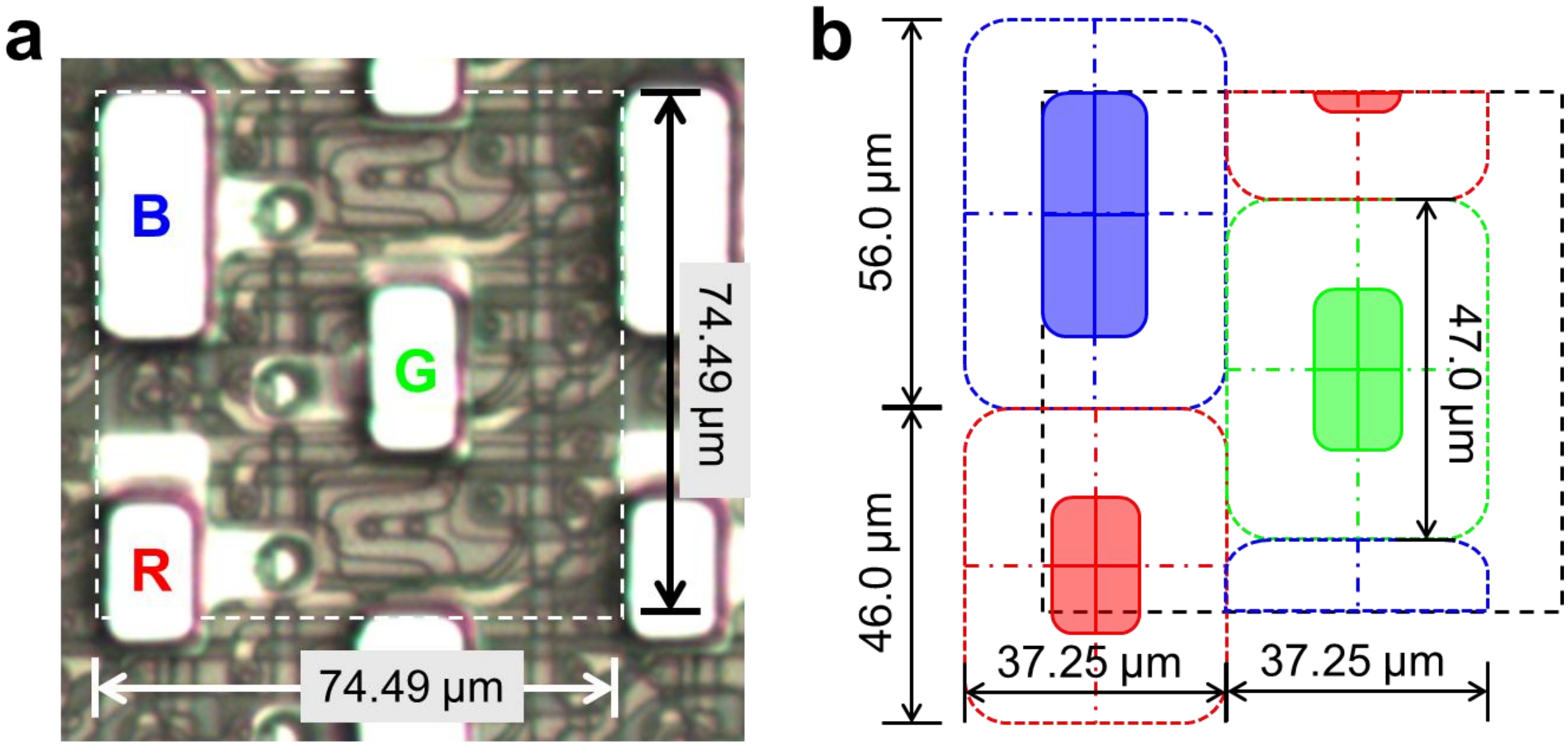

a, Optical image of a single pixel (74.49 μm × 74.49 μm) showing the emissive areas of the red (R), green (G), and blue (B) subpixels in white. **b**, Corresponding designs of the micro-bumped PDMS stamps (dashed outlines) for each colour, tailored to fully cover their respective emissive areas while remaining confined within the midpoint boundaries between neighbouring subpixels, providing mechanical tolerance for alignment during CATP.

Supplementary Fig. 29: PDMS stamp design rules and fabrication for controlled cracking and pick-up in the CATP process.

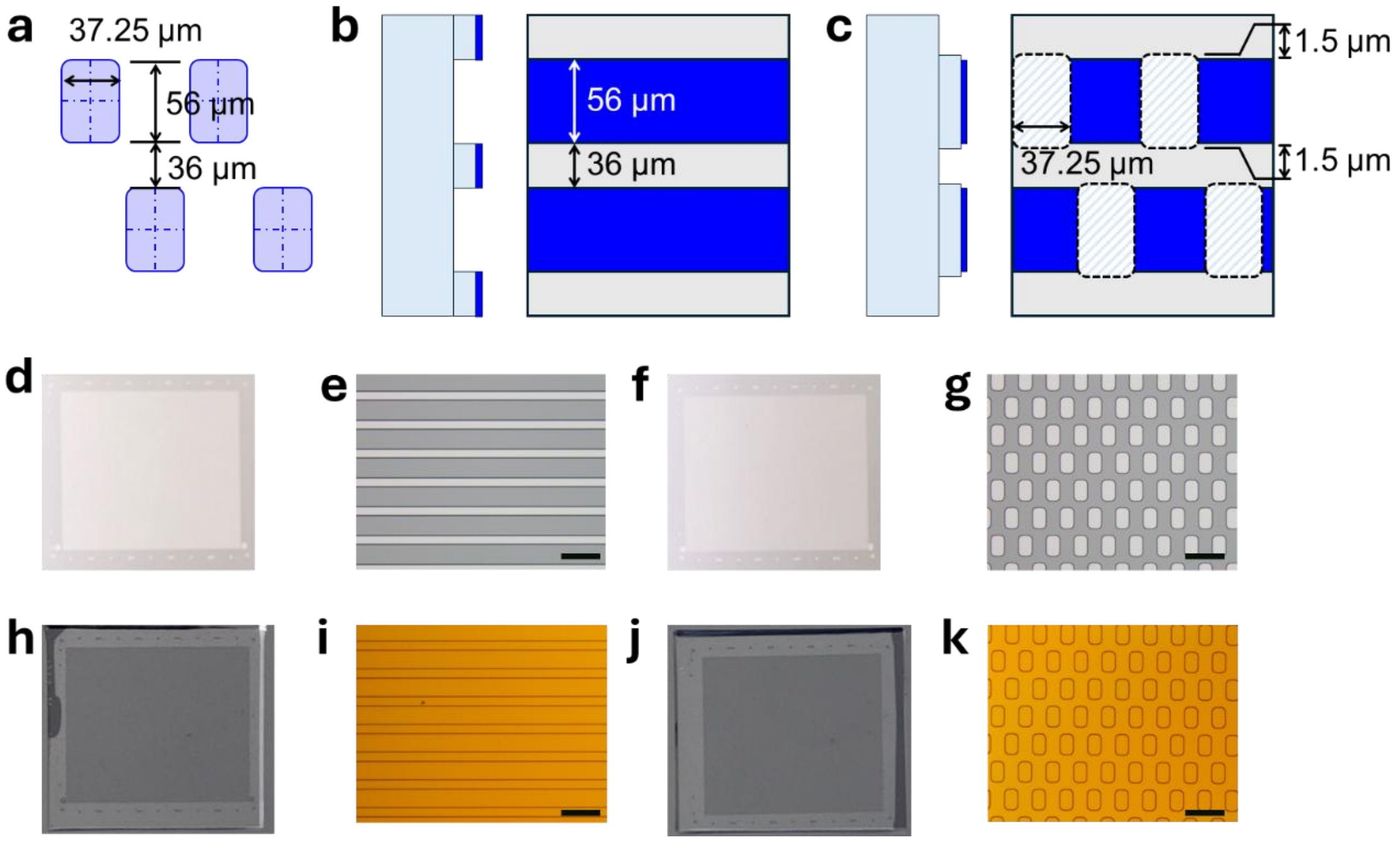

a, Planar layout of the blue subpixel in the pentile architecture, with a QD pattern size of 37.25 μm × 56 μm and a 36 μm vertical pitch. **b**, Design of the stripe-shaped PDMS bumps used in the cracking step, which segment the uniformly coated QD film into stripes without the need for alignment. The spacing between bumps is fixed at 36 μm, leaving a remaining QD stripe width of 56 μm. **c**, Geometry of the micro-bumped PDMS stamp used in the pick-up and transfer printing step, where the bump is 37.25 μm in width and enlarged by 3 μm in the vertical direction relative to the stripe width to ensure alignment-tolerant and selective pick-up of each QD stripe. **d,** Photograph of the stripe-patterned master template for the cracking step stamp. **e,** Optical microscopy (OM) image of the stripe-patterned master template for the cracking step stamp. **f,** Photograph of the pixel-patterned master template for the pick-up micro-bump stamp. **g,** OM image of the pixel-patterned master template for the pick-up micro-bump stamp. **h,** Photograph of the PDMS for the cracking step. **i,** OM image of the PDMS for the cracking step. **j,** Photograph of the PDMS stamp for dot pick-up. **k,** OM image of the PDMS stamp for dot pick-up. Scale bar: 50 μm.

Supplementary Fig. 30: Transfer printer setup for PDMS–LTPS alignment.

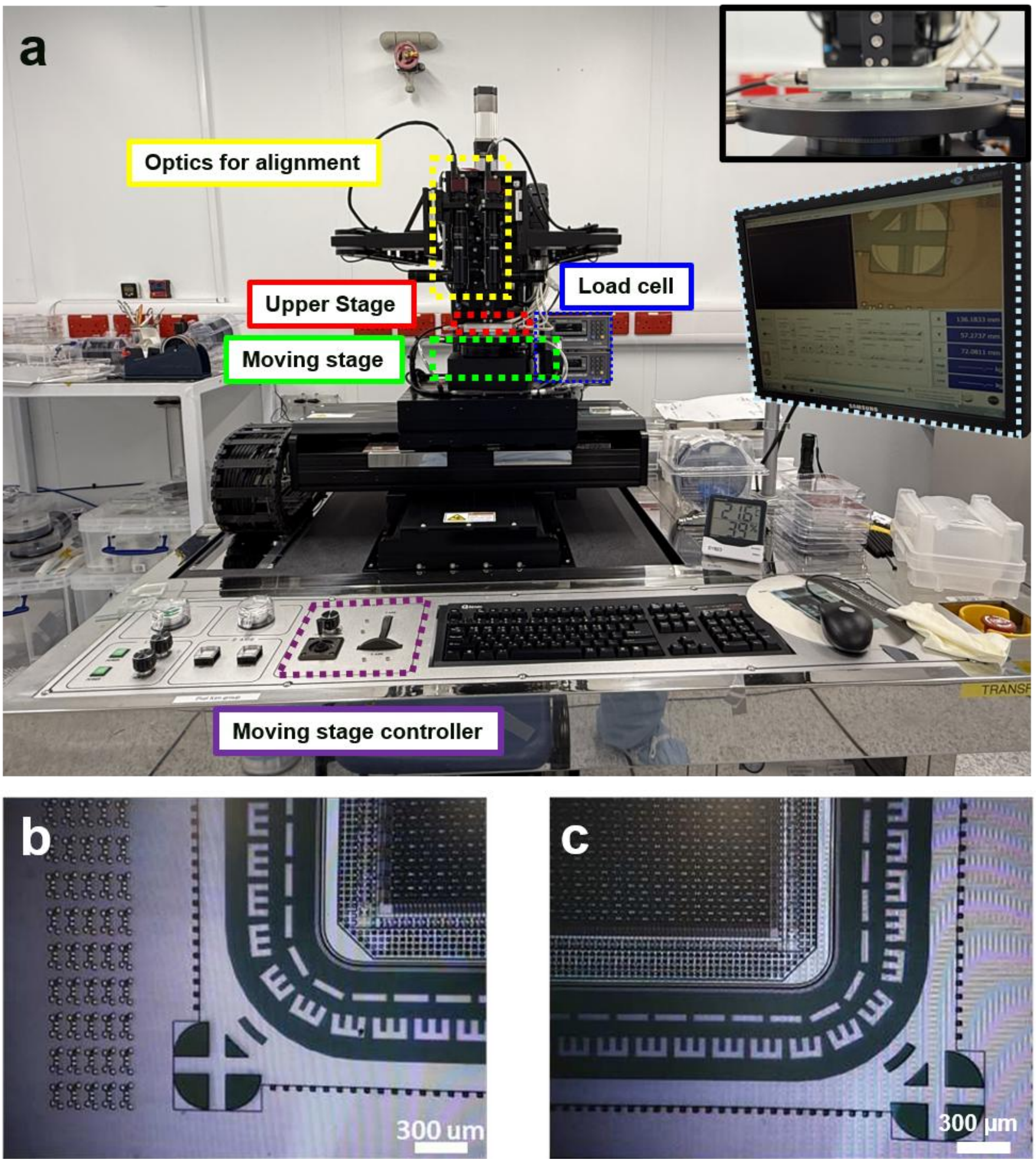

a, Photograph of the transfer printer system used for alignment, highlighting the alignment optics, upper stage, moving stage, load cell, and stage controller. Top-right inset: PDMS stamp and LTPS substrate are simultaneously in focus at the alignment working distance. **b,c,** OM images showing successful alignment between the PDMS stamp and the LTPS substrate at the left (**b**) and right (**c**) side alignment key.

Supplementary Fig. 31: Alignment accuracy of CATP-patterned red, green, and blue QD pixels on the LTPS TFT backplane.

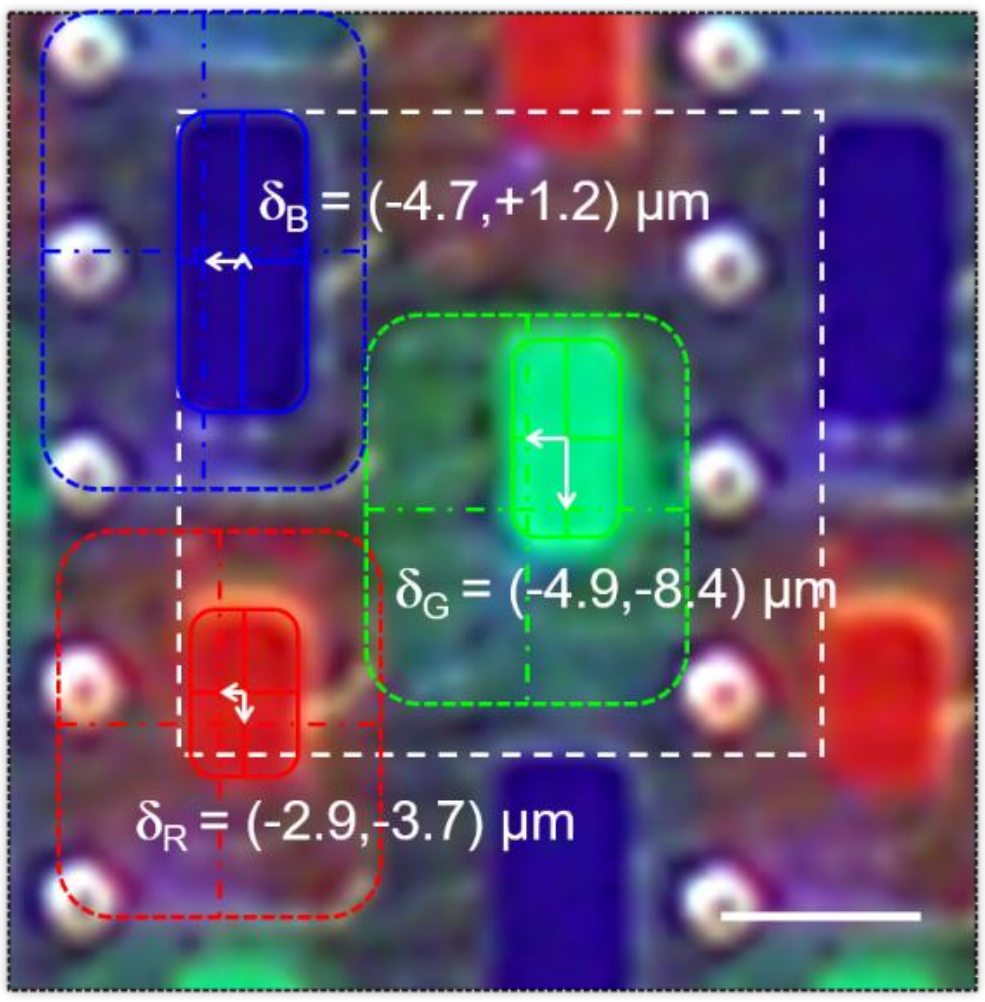

Overlay images of the transfer-printed QD pixels showing alignment offsets of (–2.9, –3.7) µm (red), (–4.9, –8.4) µm (green), and (–4.7, +1.2) µm (blue). The average deviation is (–3.8, –3.6) µm, well within the alignment margins designed for the current pixel pitch.

Supplementary Fig. 32: PL image of QDs patterned on LTPS via CATP and intaglio transfer printing.

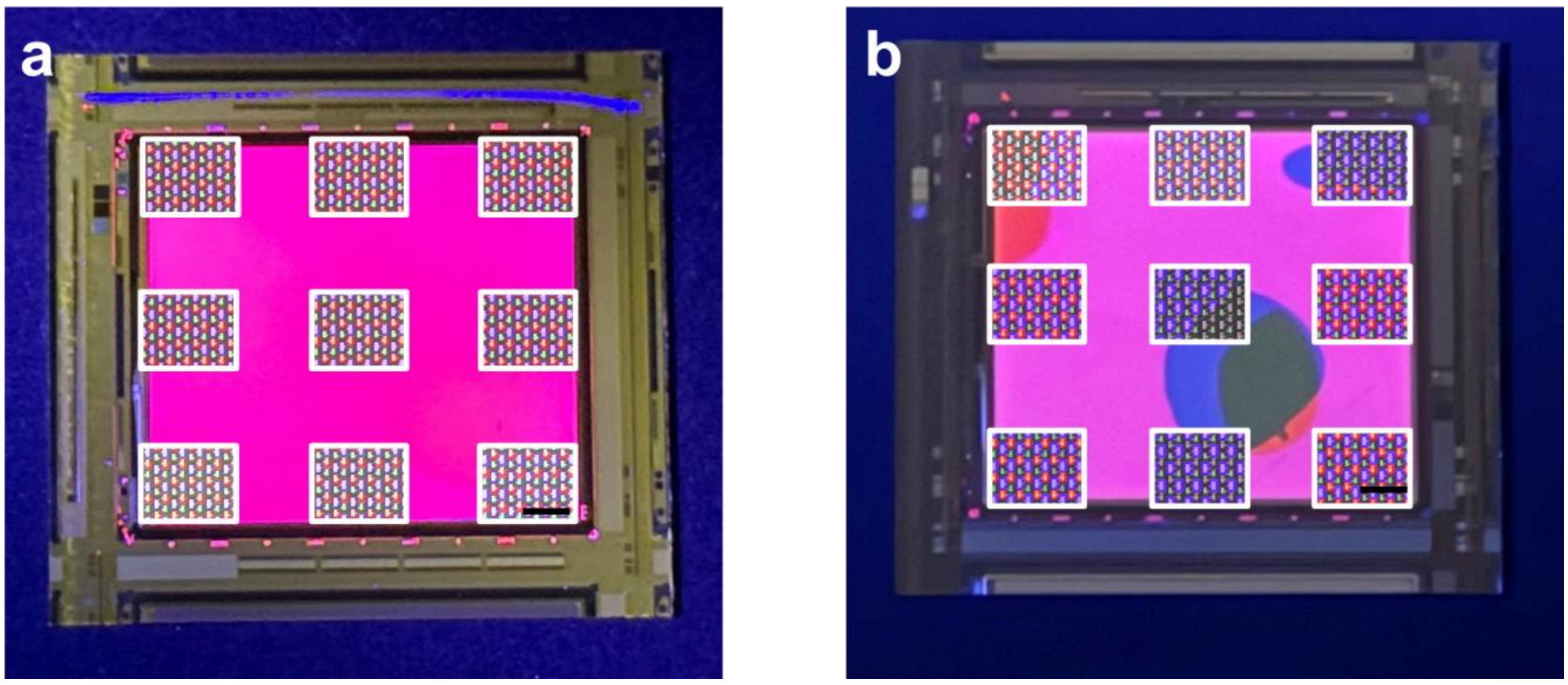

a-b, PL OM image showing nine points on the LTPS substrate of QDs patterned via CATP (**a**) and intaglio transfer printing (**b**). Black scale bar: 200 µm.

Supplementary Fig. 33: QD-LED pixel device architectures for AM EL QD display.

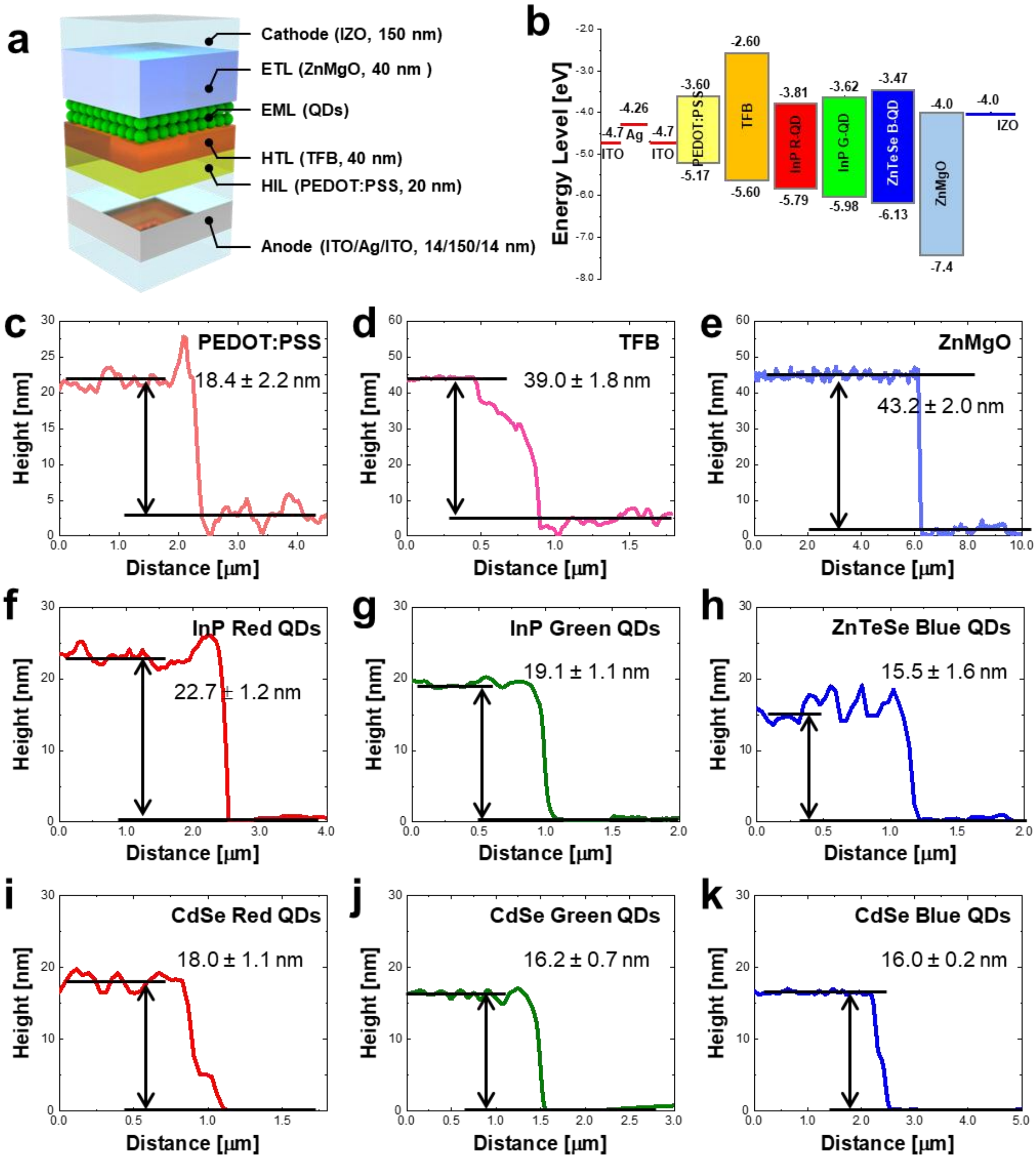

a, Multi-layered stack architecture of QD-LED devices. ITO (14 nm)/Ag (150 nm)/ITO (14 nm), PEDOT:PSS (20 nm), TFB (40 nm), QDs, ZnMgO (40 nm), and IZO (150 nm) are used for reflective anode electrode, HIL, HTL, EML, ETL, and transparent cathode electrodes, respectively. **b**, Flat band energy-band levels of each device layer under an ideal band alignment condition. **c**-**e**, Experimental measurement of thicknesses in PEDOT:PSS film for HIL (18.4 ± 2.2 nm) (**c**), TFB film for HTL (39.0 ± 1.8 nm) (**d**), ZnMgO film for ETL (43.2 ± 2.0 nm) (**e**). **f**-**h**, Experimental measurement of thicknesses in Cd-free InP red (22.7 ± 1.2 nm) (**f**), InP green (19.1 ± 1.1 nm) (**g**), and ZnTeSe blue (15.5 ± 1.6 nm) (**h**) QD layers. **i**-**k**, Experimental measurement of thicknesses in Cd-based red (18.0 ± 1.1 nm) (**i**), green (16.2 ± 0.7 nm) (**j**), and blue (16.0 ± 0.2 nm) (**k**) QD layers.

Supplementary Fig. 34: AFM topology image of ZnSeTe/ZnSe/ZnS QD films fabricated via SC and CATP.

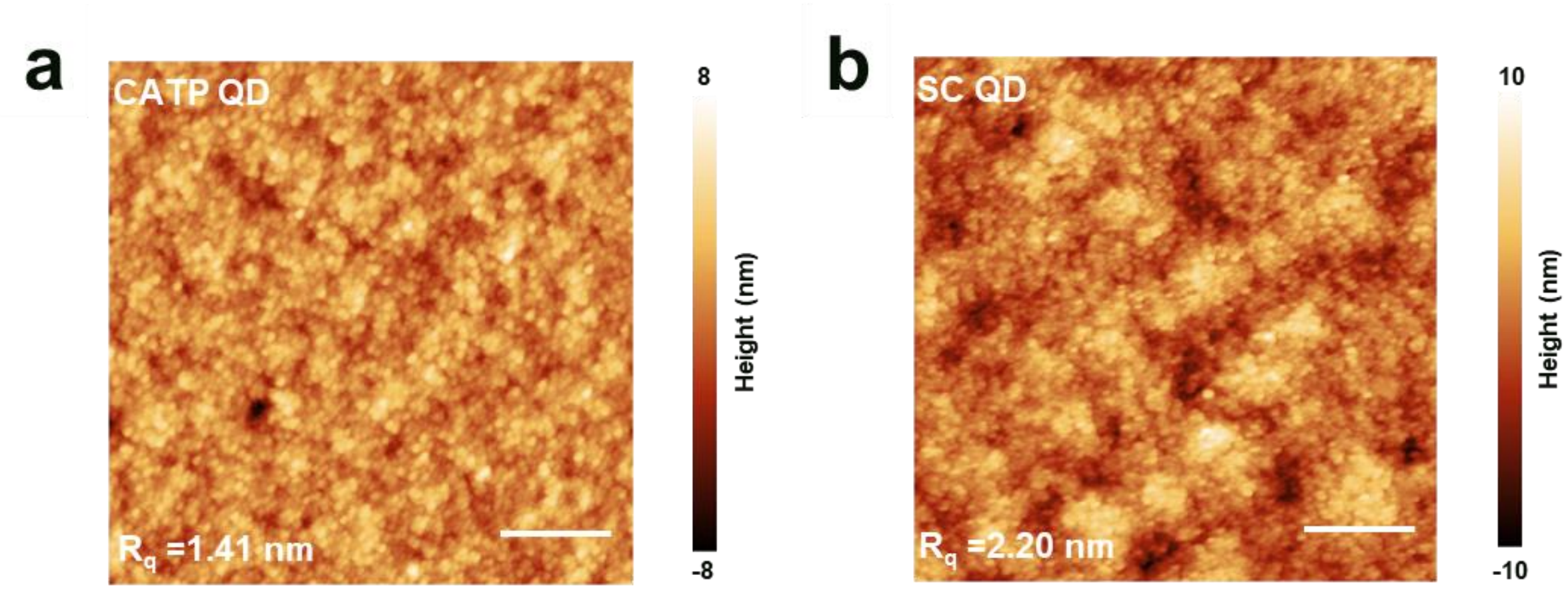

a-b, AFM topography images depicting the QD surface fabricated by CATP (**a**) and SC (**b**) on TFB coated Si substrate. Scale bar, 200 nm.

Supplementary Fig. 35: Device performance of Cd-based QD-LED devices fabricated by CATP and SC.

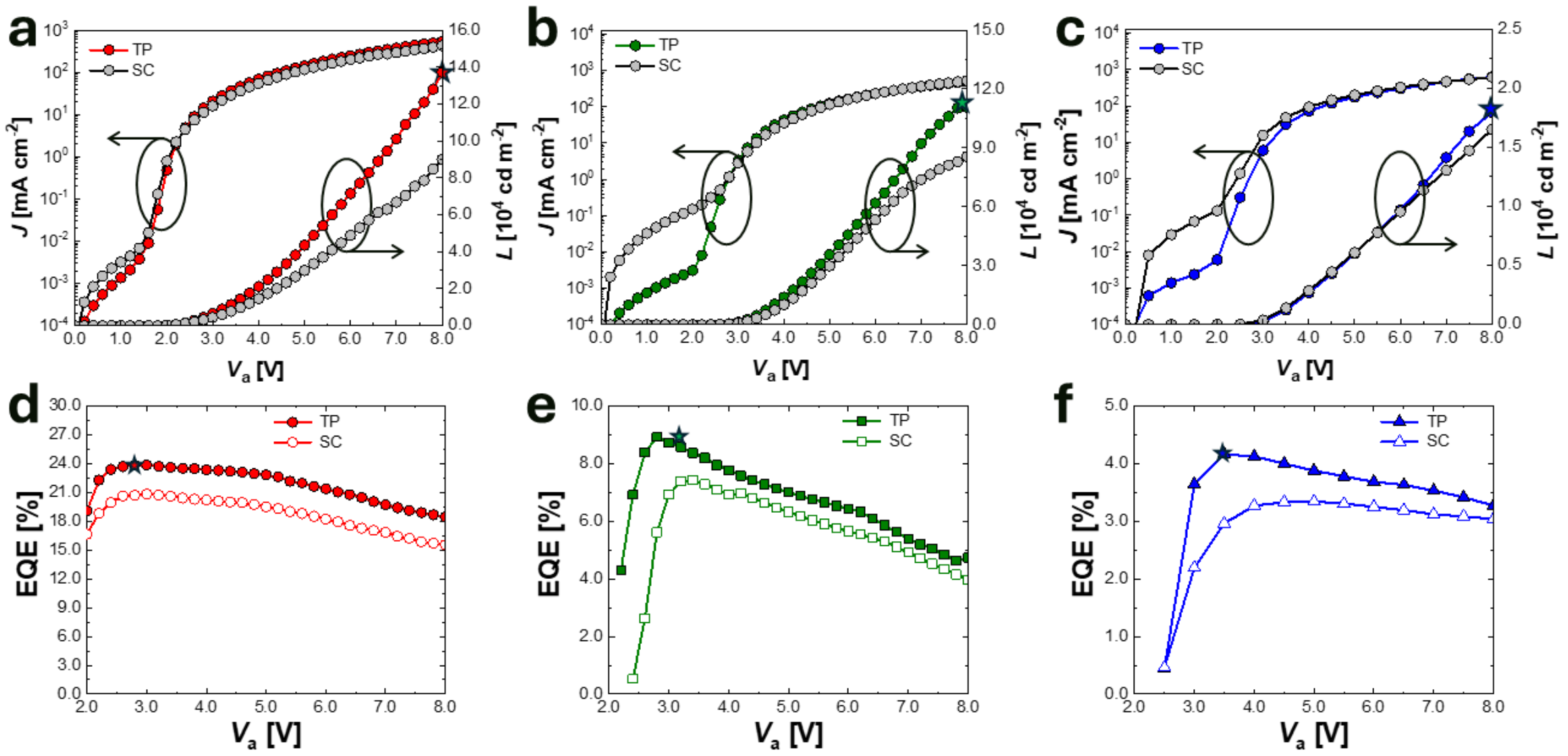

a-**c**, Current density J and luminance L as a function of the applied voltage V_a for CdSe-based red (**a**), green (**b**), and blue (**c**) QD-LED devices fabricated by CATP and SC. The devices fabricated by CATP exhibit higher luminance compared to the devices fabricated by SC. **d-f**, EQE curves as a function of current density for CdSe-based red (**d**), green (**e**), and blue (**f**) QD-LED devices fabricated by CATP and SC. Maximum EQEs of the CATP-based QD-LED devices are higher than those of the SC-based devices.

Supplementary Fig. 36: Histogram of EQEs for Cd-free red, green, and blue QD-LED devices processed by CATP and SC (N=20).

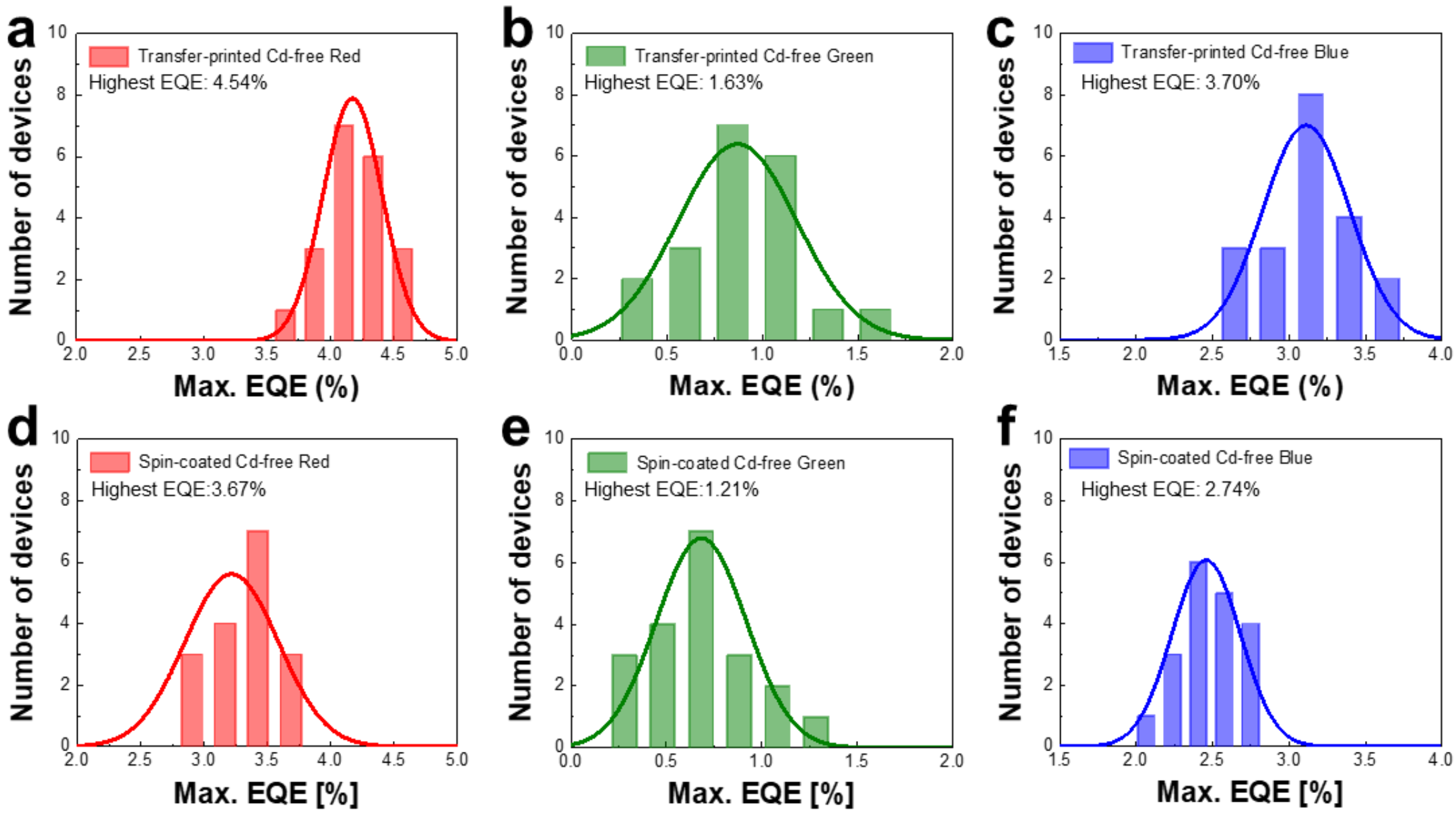

a-**c**, Histogram showing peak EQEs of Cd-free InP red (N=20) (**a**), InP green (N=20) (**b**), and ZnTeSe blue (N=20) (**c**) QD-LED devices processed by CATP. **d**-**f**, Histogram showing peak EQEs of Cd-free InP red (N=20) (**d**), InP green (N=20) (**e**), and ZnTeSe blue (N=20) (**f**) QD-LED devices processed by SC.

Supplementary Fig. 37: Refractive index and extinction coefficient of QD films fabricated via TP and SC.

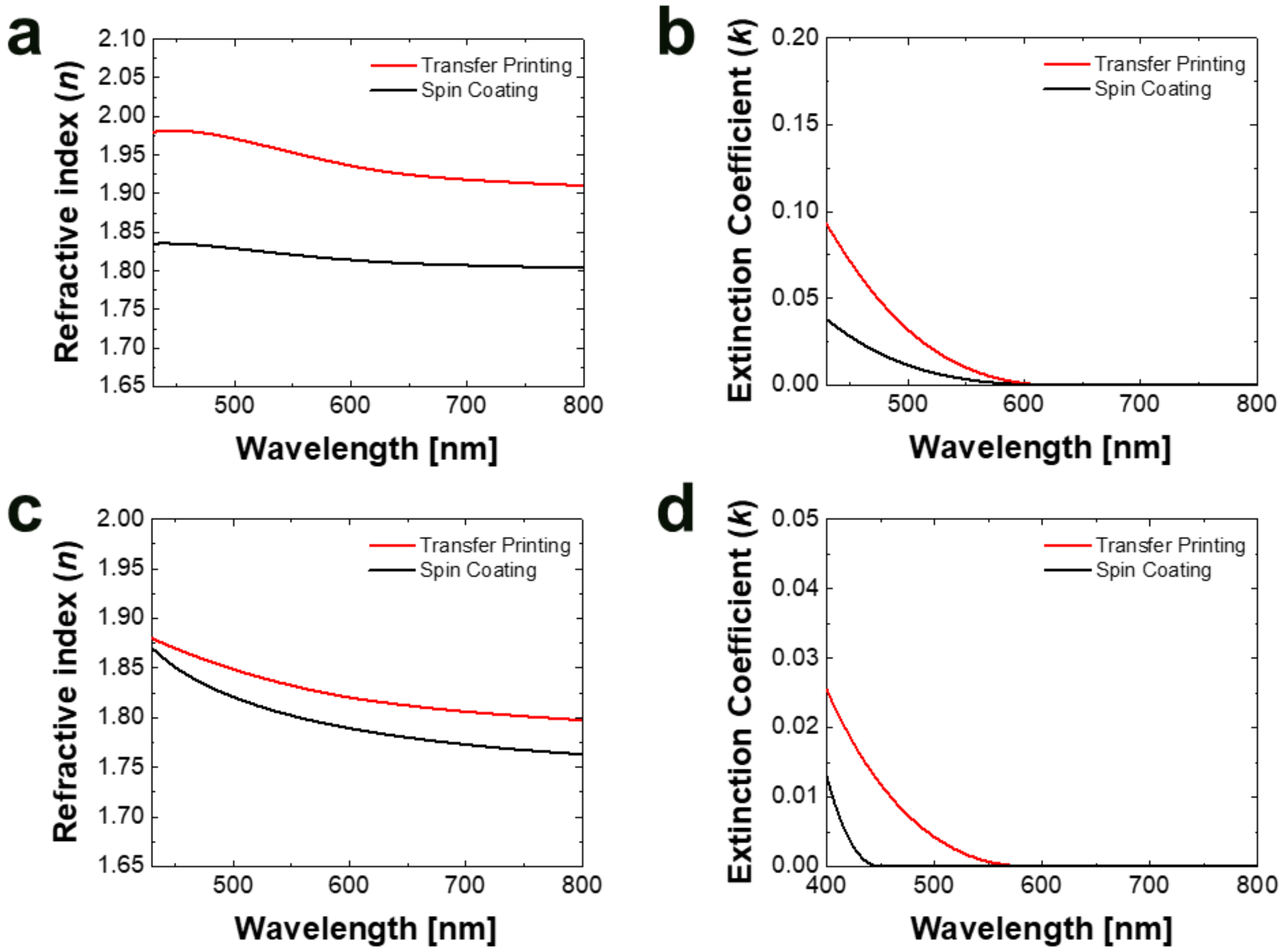

a,b, The refractive index (**a**) and extinction coefficient (**b**) of the ZnSeTe/ZnSe/ZnS QD films fabricated by spin coating and transfer printing. **c,d,** The refractive index (**c**) and extinction coefficient (**d**) of the CdSe/ZnS QD films fabricated by spin coating and transfer printing.

Supplementary Fig. 38: Analysis and characterisation of QD-LEDs fabricated via TP and SC.

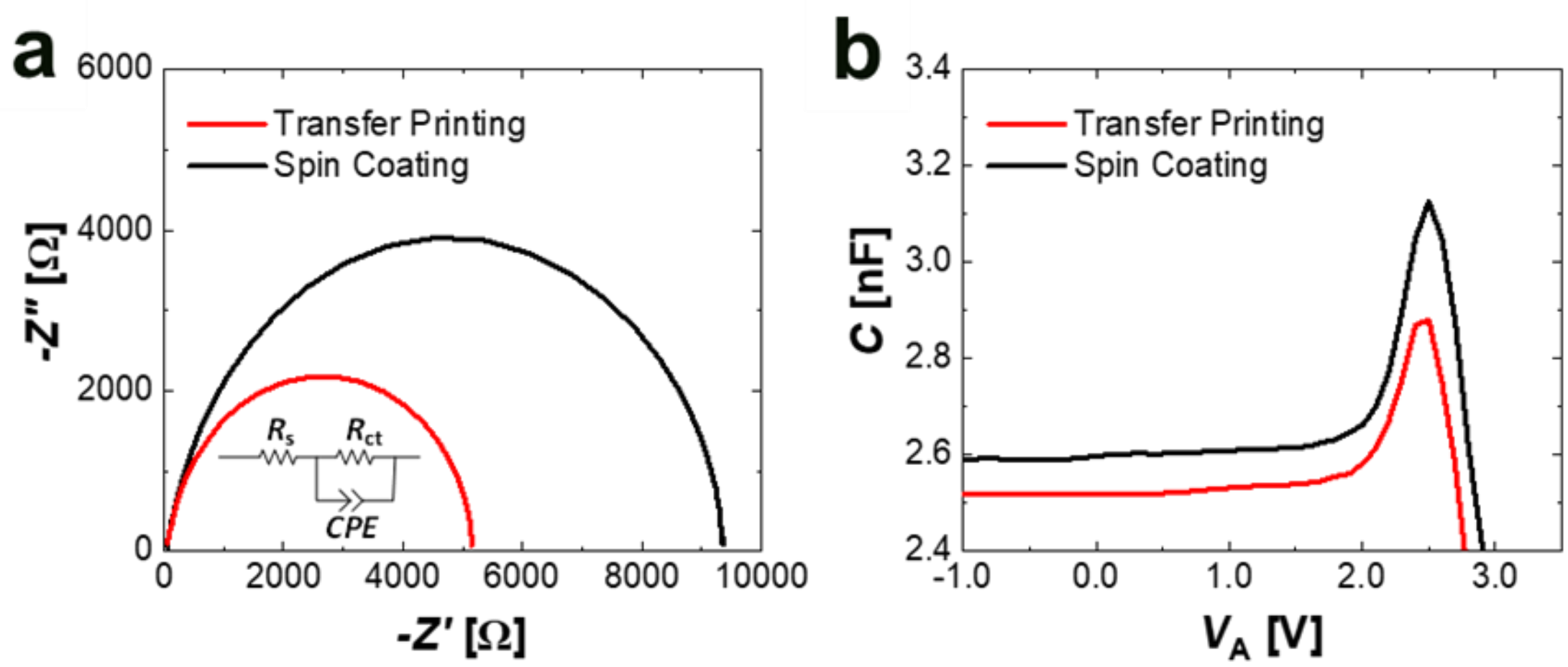

a, Nyquist plots of impedance spectra for QD-LEDs fabricated via spin coating and transfer printing (Inset: equivalent circuit model used for the analysis R_s: series resistance; R_{ct}: charge-transfer resistance; CPE: constant phase element). **b,** The capacitance-voltage characteristics of QD-LEDs fabricated via spin coating and transfer printing.

Supplementary Fig. 39: Analysis and characterisation of single carrier devices and EL spectra of QD-LED fabricated via TP and SC.

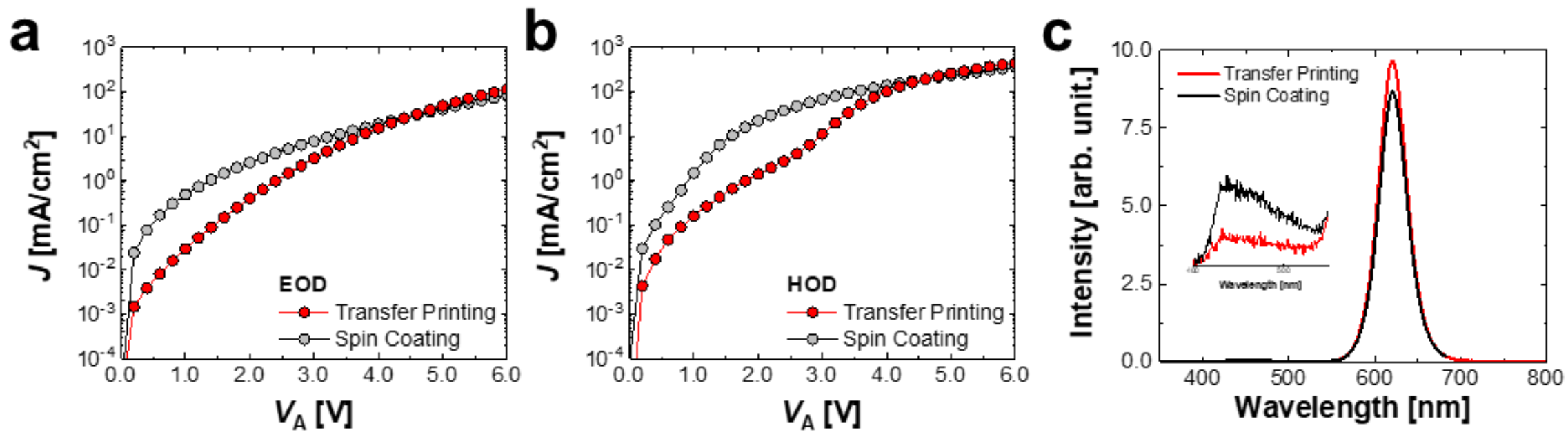

a-b, Current density–voltage (J–V) characteristics of the electron-only devices (EOD) consisting of ITO/ZnMgO/InP red QDs/ZnMgO/Al (**a**) and hole-only devices (HOD) ITO/PEDOT:PSS/TFB/InP red QDs/MoO_3/Al (**b**) fabricated using spin coating and transfer printing. **c,** EL spectra of QD-LEDs based on transfer printed and spin-coated InP red QD films at the same current density (~600 mA cm^{-2}) (Inset: zoomed-in views of EL spectra from the HTL).

Supplementary Fig. 40: Characterisation of pixel crosstalk between ZnMgO ETL layer.

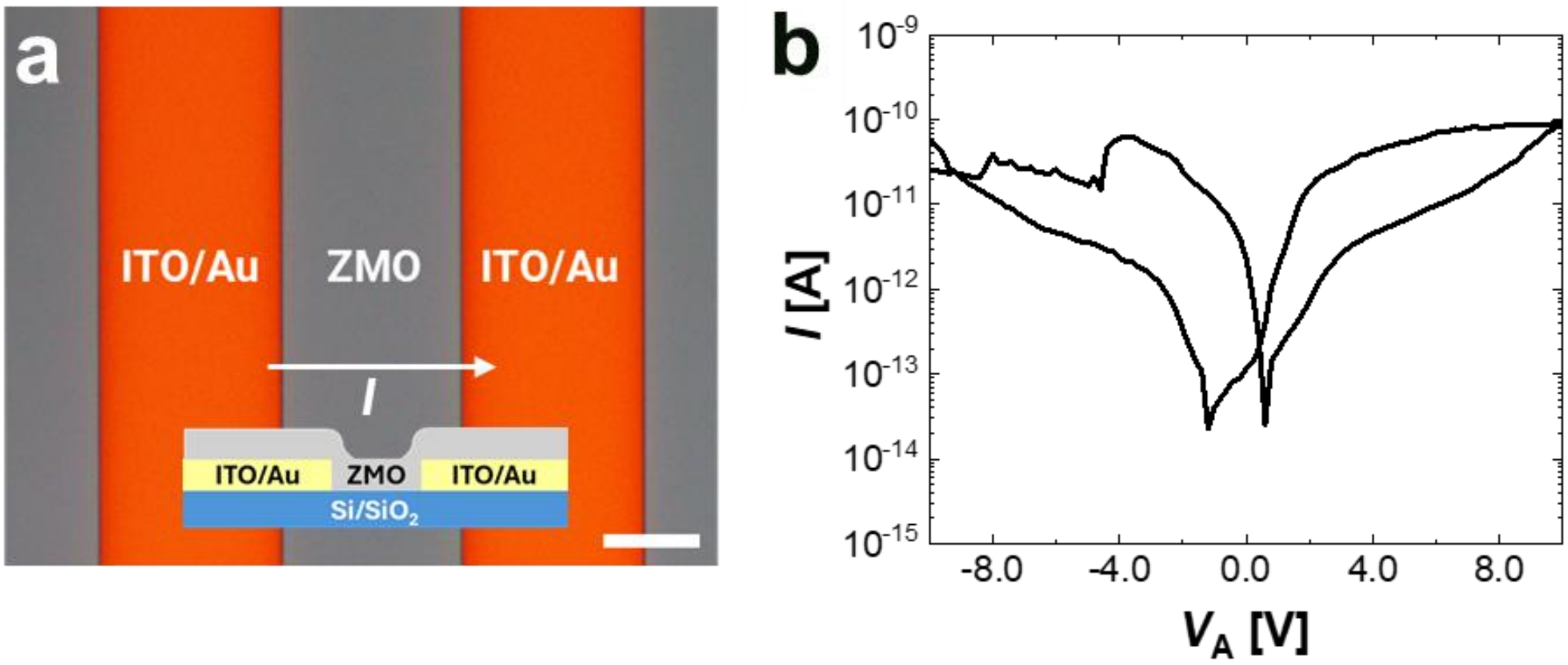

a, OM image of the test structure showing ITO/Au electrodes spaced 20 µm apart. The bottom schematic illustrates the corresponding cross-section. Scale bar: 10 µm. **b,** crosstalk less than 1 pA was observed in the I-V sweep from -10 V to 10V of the test structure.

Supplementary Fig. 41: Electroluminescence (EL) images of spin coated monochromatic AM QD-LED Displays.

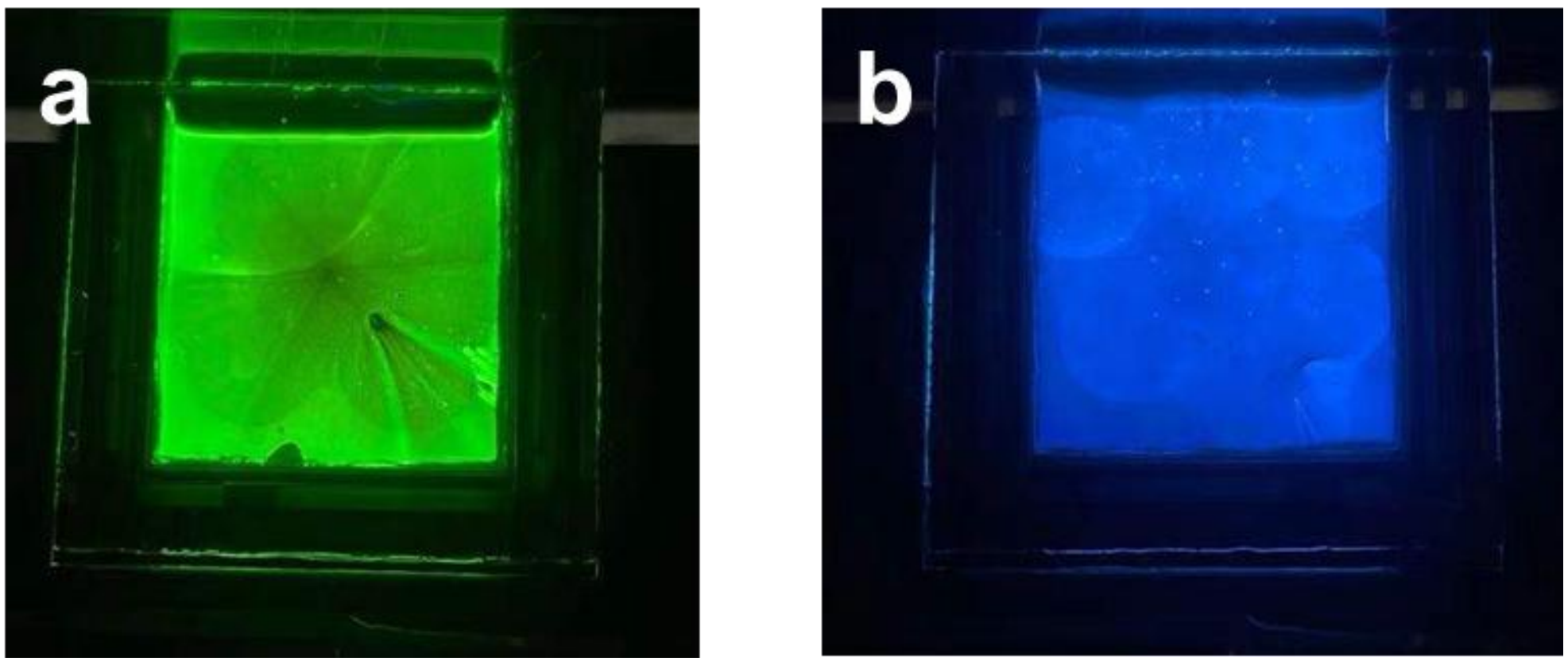

EL images of spin-coated monochromatic AM QD-LED displays. **a,** Cd-based green QD and **b,** Cd-based blue QD.

Supplementary Fig. 42: Material-level analysis on the spectral properties of Cd-free and Cd-based QDs to support system-level colour analysis in 1.41-inch full-colour AM EL QD display systems shown in Fig. 3e and f.

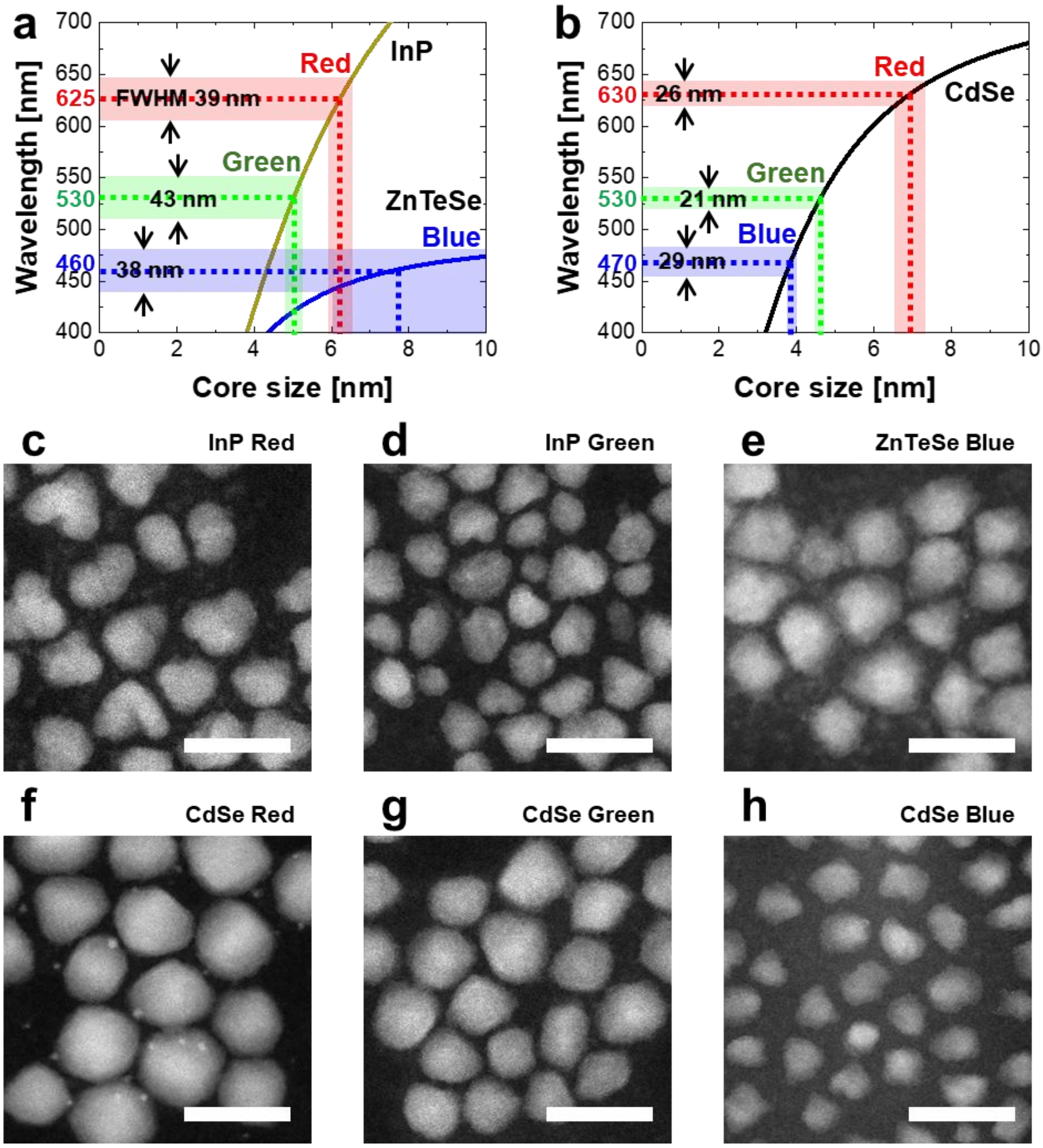

a,**b**, Core-size dependent emission wavelength for Cd-free (**a**) and Cd-based (**b**) QD materials, along with their experimental FWHMs. InP/ZnS red (625 nm) and green (530 nm), and ZnTeSe/ZnSe/ZnS blue (460 nm) QDs are utilised for Cd-free materials. CdSe/ZnS red (630 nm), green (530 nm), and blue (470 nm) QDs are utilised for Cd-based materials. Cd-free QDs exhibit wider FWHMs of 39 nm (InP red), 43 nm (InP green), and 38 nm (ZnSe) in contrast to narrower FWHMs of 26 nm (red), 21 nm (green), and 29 nm (blue) in Cd-based QDs. Cd-free (InP and ZnTeSe) QDs demonstrate a wider range in core size compared to Cd-based counterparts. **c**-**h**, Particle size distributions of the InP red, InP green, ZnTeSe blue, CdSe red, green, and blue QDs taken from a HAADF-STEM microscope for experimental validation of the core-size dependencies in Cd-free and Cd-base QD materials. Scale bars, 20 nm.

Supplementary Fig. 43: Effect of IZO sputtering power on the electrical and optical performance of Cd-based green QD-LEDs.

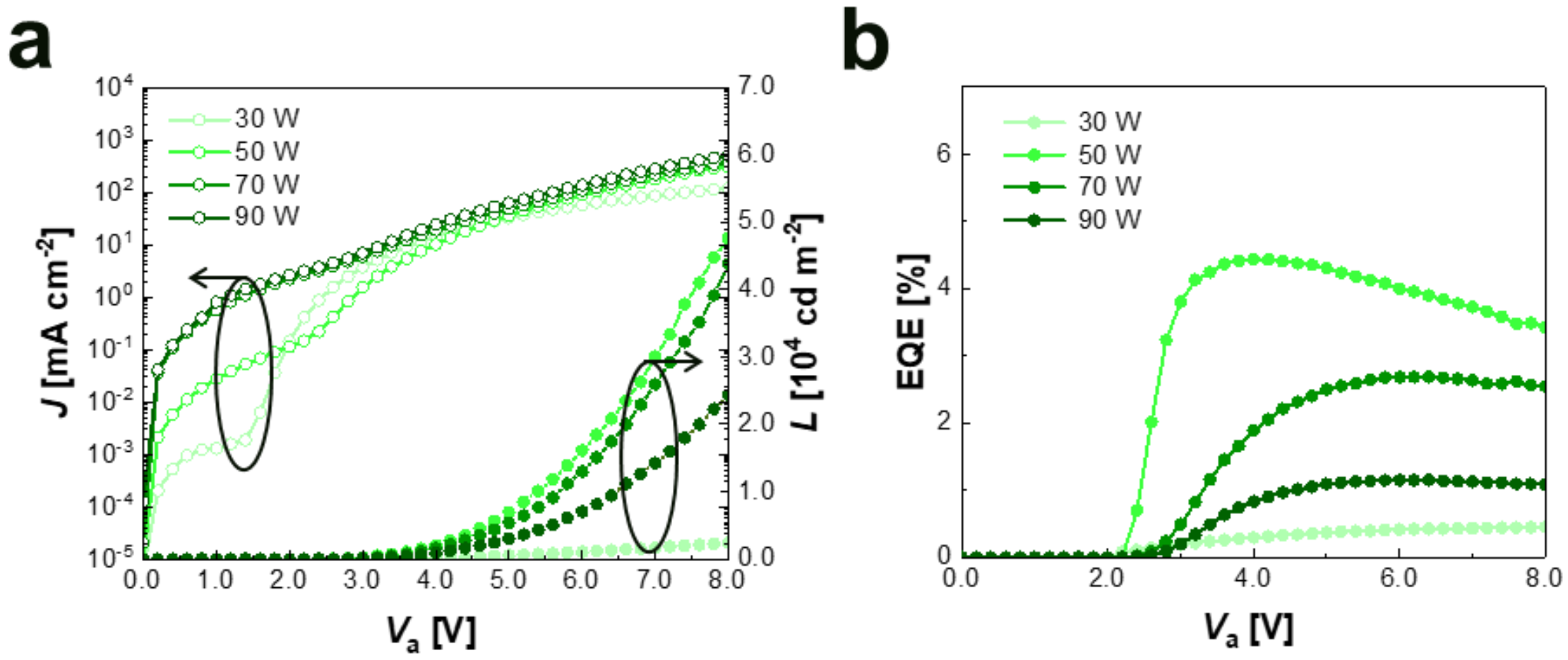

a, Current density *J* and luminance *L* as a function of the applied voltage V_a for Cd-based green QD-LEDs with IZO sputtered at 30 W, 50 W, 70 W, and 90 W. **b,** EQE as a function of V_a for the corresponding devices.

Supplementary Table 1. Comparison of research progress of patterned Cd-based QD-LEDs in terms of maximum luminance, peak EQE and minimum pixel size. Devices with different colours - red, green, blue, and white - are labelled as (R), (G), (B), and (W), respectively.

Year	Patterning method	Maximum luminance [cd m^{-2}]	EQE [%]	Lifetime [h]	Minimum pixel size [μm]	Ref
2011	Transfer printing	16380 (R)<br>6425 (G)<br>423 (B)	-	T50 at 1050 cd m^{-2} 76.5 h (R)	0.35	S5
2015	Transfer printing	14000 (G)<br>3940 (W)	2.35 (G)<br>1.6 (W)	T50 at 4554 cd m^{-2} 41.7 h (G)<br>T50 at 100 cd m^{-2} 12815 h (G), n=1.5	0.3	S10
2015	Transfer printing	2497 (R)<br>14102 (G)<br>265 (B)	1.93 (R)<br>2.33 (G)<br>0.32 (B)	-	2	S17
2015	Electrohydrodynamic jet printing	11250 (R)<br>36000 (G)	2.6 (R)<br>2.5 (G)	-	0.4	S18
2016	Transfer printing	14700 (R)<br>9400 (G)<br>28 (B)	2.7 (R)<br>1.68 (G)<br>0.03 (B)	T50 at 2037 cd m^{-2} 35 h (R)	60	S19
2020	Immersion transfer printing	10711 (G)	3.3 (G)	-	0.005	S12
2020	Inkjet printing	12300 (R)	16.6 (R)	T95 at 3462 cd m^{-2} 196 h (R)<br>T95 at 1000 cd m^{-2} 1833 h (R), n=1.8	50	S20
2021	Inkjet printing	104826 (R)<br>283996 (G)<br>2367 (B)	19.3 (R)<br>18 (G)<br>4.4 (B)	T95 at 9415 cd m^{-2} 40 h (R)<br>T95 at 1000 cd m^{-2} 2264 h (R), n=1.8<br>T50 at 11465 cd m^{-2} 312 h (R)<br>T50 at 24981 cd m^{-2} 63 h (G)<br>T50 at 103 cd m^{-2} 46 h (B)	150	S21
2021	Electrophoretic deposition	79489 (R)<br>67111 (G)	-	-	2	S15
2022	Langmuir–Blodgett Transfer printing	262400 (R)<br>411045 (G)	14.72 (R)<br>7.7 (G)	-	0.5	S13
2022	Transfer printing	19670 (R)<br>10370 (G)<br>8070 (C)<br>442 (B)<br>3258 (W)	4.3 (R)<br>0.6 (G)<br>1 (C)<br>0.3 (B)<br>1.4 (W)	T95 at 10640 cd m^{-2} 1.9 h (R)<br>T50 at 100 cd m^{-2} 39899 h (R), n=1.58	6	S22
2020	Photolithography	476500 (R)<br>89700 (G)<br>3150 (B)	14.6 (R)<br>5.9 (G)<br>2.9 (B)	T95 at 11000 cd m^{-2} 14.4 h (R)	2	S6
2022	Photolithography	11330 (R)	11.7 (R)	T95 at ? cd m^{-2} 191.6 h (R)<br>T95 at 1000 cd m^{-2} 4852 h (R), n=?	3	S23
2022	Photolithography	234600 (R)<br>264700 (G)	20 (R)<br>15 (G)	T95 at 30mA cm^{-2} 43.7 h (R)<br>T95 at 30mA cm^{-2} 14.4 h (G)	0.8	S7
2022	Microwetting	8700 (R)<br>213356 (G)<br>25730 (B)	-	-	0.8	S16
2024	Inkjet printing	31740 (R)<br>59420 (G)	23.08 (R)<br>22.43 (G)	T50 at 7220 cd m^{-2} 155 h (R)<br>T50 at 100 cd m^{-2} 343342 h (R), n=1.8<br>T50 at 18100 cd m^{-2} 129.5 h (G)<br>T50 at 100 cd m^{-2} 1500463 h (G), n=1.8	400	S24
2024	Transfer printing	11700 (R)<br>100471 (G)<br>1330 (B)	13 (R)<br>23.3 (G)<br>4.1 (B)	-	0.4	S14
This work	Transfer printing	75043 (R)<br>110800 (G)<br>17950 (B)	14.3 (R)<br>8.9 (G)<br>4.1 (B)	T95 at 10870 cd m^{-2} 55.1 h (R)<br>T95 at 1000 cd m^{-2} 4039.8 h (R), n=1.8<br>T95 at 100 cd m^{-2} 254897 h (R), n=1.8	0.6	

Supplementary References

S1. Okamoto, S. *et al.* 23.2: Invited Paper: Challenges for Realizing QD-LED. in *SID Symposium Digest of Technical Papers* vol. 53 265–268 (Wiley Online Library, 2022).

S2. Okamoto, S. *et al.* 61-1: Invited Paper: Development of Active-Matrix nanoLED Display with Cadmium Free QDs Patterned by Photolithography Process in the Atmosphere. in *SID Symposium Digest of Technical Papers* vol. 54 865–868 (Wiley Online Library, 2023).

S3. Park, M. *et al.* All inkjet-printed 6.95 ″217 ppi active matrix QD-LED display with RGB Cd-free QDs in the top-emission device structure. *J Soc Inf Disp* **30**, 433–440 (2022).

S4. Ha, J. *et al.* Dual ligand exchange of Cd-free quantum dots and optimal control of ink formulation for improving the performance of all-inkjet-printed quantum dot light-emitting diodes. *J Soc Inf Disp* (2024).

S5. Kim, T.-H. *et al.* Full-colour quantum dot displays fabricated by transfer printing. *Nat Photonics* **5**, 176–182 (2011).

S6. Yang, J. *et al.* High-resolution patterning of colloidal quantum dots via non-destructive, light-driven ligand crosslinking. *Nat Commun* **11**, 2874 (2020).

S7. Hahm, D. *et al.* Direct patterning of colloidal quantum dots with adaptable dual-ligand surface. *Nat Nanotechnol* **17**, 952–958 (2022).

S8. Yang, J. *et al.* Nondestructive Photopatterning of Heavy-Metal-Free Quantum Dots. *Advanced Materials* **34**, 2205504 (2022).

S9. Lee, J. *et al.* Direct Optical Lithography of Colloidal InP-Based Quantum Dots with Ligand Pair Treatment. *Cite This: ACS Energy Lett* **8**, 4210–4217 (2023).

S10. Choi, M. K. *et al.* Wearable red–green–blue quantum dot light-emitting diode array using high-resolution intaglio transfer printing. *Nat Commun* **6**, 7149 (2015).

S11. Keum, H. *et al.* Photoresist contact patterning of quantum dot films. *ACS Nano* **12**, 10024–10031 (2018).

S12. Nam, T. W. *et al.* Thermodynamic-driven polychromatic quantum dot patterning for light-emitting diodes beyond eye-limiting resolution. *Nat Commun* **11**, 3040 (2020).

S13. Meng, T. *et al.* Ultrahigh-resolution quantum-dot light-emitting diodes. *Nat Photonics* **16**, 297–303 (2022).

S14. Yoo, J. *et al.* Highly efficient printed quantum dot light-emitting diodes through ultrahigh-definition double-layer transfer printing. *Nat Photonics* 1–8 (2024).

S15. Zhao, J. *et al.* Large-area patterning of full-color quantum dot arrays beyond 1000 pixels per inch by selective electrophoretic deposition. *Nat Commun* **12**, 4603 (2021).

S16. Song, K. M. *et al.* Noninvasive and Direct Patterning of High-Resolution Full-Color Quantum Dot Arrays by Programmed Microwetting. *ACS Nano* **16**, 16598–16607 (2022).

S17. Cho, H. *et al.* Soft contact transplanted nanocrystal quantum dots for light-emitting diodes: effect of surface energy on device performance. *ACS Appl Mater Interfaces* **7**, 10828–10833 (2015).

S18. Kim, B. H. *et al.* High-resolution patterns of quantum dots formed by electrohydrodynamic jet printing for light-emitting diodes. *Nano Lett* **15**, 969–973 (2015).

S19. Kim, B. H. *et al.* Multilayer transfer printing for pixelated, multicolor quantum dot light-emitting diodes. *ACS Nano* **10**, 4920–4925 (2016).

S20. Xiang, C. *et al.* High efficiency and stability of ink-jet printed quantum dot light emitting diodes. *Nat Commun* **11**, 1–9 (2020).

S21. Jia, S. *et al.* High performance inkjet-printed quantum-dot light-emitting diodes with high operational stability. *Adv Opt Mater* **9**, 2101069 (2021).

S22. Samarakoon, C. *et al.* Optoelectronic system and device integration for quantum-dot light-emitting diode white lighting with computational design framework. *Nat Commun* **13**, 4189 (2022).

S23. Lu, S. *et al.* Beyond a linker: the role of photochemistry of crosslinkers in the direct optical patterning of colloidal nanocrystals. *Angewandte Chemie International Edition* **61**, e202202633 (2022).

S24. Wei, C. *et al.* Highly ordered inkjet-printed quantum-dot thin films enable efficient and stable QLEDs with EQE exceeding 23%. *eScience* **4**, 100227 (2024).

S25. Lee, S.-H. *et al.* Heterostructural tailoring of blue ZnSeTe quantum dots toward high-color purity and high-efficiency electroluminescence. *Chemical Engineering Journal* **429**, 132464 (2022).
